# Reciprocal coupling between EMT and BMP morphogen signalling drives collective cell invasion

**DOI:** 10.1038/s44318-026-00916-8

**Published:** 2026-10-07

**Authors:** Yuri Takahashi, Alexandra Neaverson, Lara Busby, Filip Twarowski, Carlos Camacho-Macorra, Guillermo Serrano Nájera, Benjamin Steventon

**Affiliations:** 1 Department of Genetics, University of Cambridge, Cambridge, CB2 3EH UK; 2 Present Address: University of California, Berkeley, CA USA

**Keywords:** Cell Adhesion, Polarity & Cytoskeleton, Development, Signal Transduction

## Abstract

During collective cell invasion, epithelial-to-mesenchymal transition (EMT) and morphogen signalling-mediated cell fate specification are traditionally viewed as a linear cascade: morphogens drive cell fates that activate EMT gene expression programs. Here, we uncover reciprocal coupling between EMT initiation and BMP signalling mediated by *SNAI2* and *SMAD1*. Using ex vivo chick tissue explants, we demonstrate that substrate-induced migration upregulates *SMAD1* expression, activating BMP signalling across germ layers. Single-cell RNA sequencing reveals *SNAI2* and *SMAD1* co-expression in EMT initiation regions, and *SNAI2* overexpression is sufficient to induce ectopic *SMAD1* expression in vivo. Ex vivo CRISPR-Cas9 experiments further demonstrate that *SNAI2* is required for *SMAD1* expression and BMP signalling. While BMP signalling is dispensable for EMT initiation, it regulates cell fate proportions, dispersal dynamics, precursor region depletion rates, and migration directionality. This coupling provides a mechanism for synchronising cell fate specification with invasion progression during axis elongation, positioning *SNAI2-SMAD1* interaction as mechanism to coordinate cell fate specification and EMT during collective cell invasion.

## Synopsis

Collective cell invasion is classically viewed as a morphogen-driven process comprising cell fate specification and subsequent activation of epithelial-to-mesenchymal transition (EMT) gene expression programmes. This work establishes EMT initiation as an upstream inducer of BMP pathway activation during vertebrate development, establishing reciprocal coupling of EMT and morphogen-induced cell fate changes as a basis for coordinated cell migration across germ layers.

- Ex vivo induction of cell migration in chick neuroectodermal and mesodermal tissue explants triggers BMP pathway activation and drives convergent cell fate shifts towards migratory cell states.
- *SNAI2*-dependent regulation of *SMAD1* expression couples EMT initiation to BMP signalling activity.
- BMP signalling is restricted to regions of EMT initiation during development.
- *SNAI2* is required for *SMAD1* expression and for priming of BMP activity ex vivo.
- BMP signalling is dispensable for EMT initiation but regulates cell fate proportions, dispersal dynamics and migration directionality.

## Abstract (web summary)

Epithelial-to-mesenchymal transition initiation activates morphogen-mediated cell fate specification to coordinate collective cell migration across germ layers.

## Graphical abstract

**Figure:**
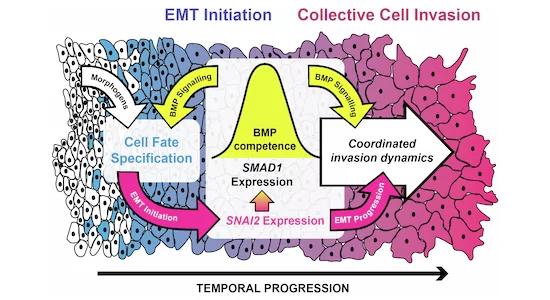

## Introduction

Collective cell invasion is the coordinated multi-step process where groups of cells penetrate tissue boundaries and move through complex environments. This process encompasses ingression, delamination, and migration, therefore requiring tight spatio-temporal coordination of cell behaviours. Two critical examples occur during early vertebrate development: mesoderm ingression through the primitive streak during gastrulation (reviewed in Chuai et al, 2012; Tam and Behringer, 1997; see also Voiculescu et al, 2014), which continues throughout axis elongation, and neural crest cell delamination during neurulation (reviewed in Theveneau and Mayor, 2012; see also Theveneau et al, 2010). In both contexts, populations of cells must simultaneously undergo epithelial-to-mesenchymal transition (EMT) while specifying appropriate migratory cell fates—a coordination challenge that demands precise integration of morphogen signalling with cellular state transitions.

Our current understanding of how collective cell invasion is regulated is based on a linear cascade model in which morphogens act upstream of cell fate specification and EMT (reviewed in Acloque et al, 2009; Thiery et al, 2009; Leathers and Rogers, 2022; Zhang et al, 2025). Morphogens such as Wnts, FGFs, and BMPs first specify cell fates that are primed to undergo migration as part of their differentiation programme. These fate-specified cells then activate transcriptional programmes (driven by key EMT drivers such as the SNAIL and ZEB family transcription factors) that repress epithelial gene programmes and activate mesenchymal ones that enable cells to lose epithelial polarity and remodel the surrounding extracellular matrix (reviewed in Debnath et al, 2022; see also Duong and Erickson, 2004; Brown et al, 2021). However, this linear cascade does not easily explain the tight population-level spatio-temporal coordination observed during collective cell invasion. Recent work has revealed that self-organised collective cell behaviours can emerge from reciprocal interactions between cellular state transitions and morphogen signalling, in which cellular behaviours both generate and respond to tissue-scale properties (Pfeifer et al, 2024). This raises a fundamental question: do reciprocal interactions exist between EMT initiation and morphogen signalling to drive collective cell invasion?

To investigate potential reciprocal coupling between EMT initiation and morphogen signalling, we employed an experimental system to induce cell migration independently of developmental context. We adapted a chick embryo explant assay (Sanders, 1980; Newgreen, 1984; Busby et al, 2024) where mesodermal tissues are dissected and plated on extracellular matrix (ECM)-coated glass to assess migratory behaviour. We used this assay to compare two gastrula-stage chick embryonic tissues of different endogenous EMT states: the anterior primitive streak (APS) and the prospective neural plate (PNP). In both populations, *SNAI2* expression increased together with BMP pathway activation and subsequent BMP-induced cell fate changes. In vivo and ex vivo functional studies demonstrated that *SNAI2* overexpression is sufficient and required for *SMAD1* expression and BMP signalling activity. We also find that BMP signalling was dispensable for EMT initiation in vivo, prompting the question: Does BMP signalling regulate the dynamics of collective cell invasion itself? To address this, we turned to in vivo grafting experiments that revealed that BMP signalling modulates cell dispersal dynamics, precursor region depletion rates, and migration directionality. Taken together, these findings reveal a reciprocal coupling mechanism in which SNAI2 initiation directly primes BMP pathway activity through *SMAD1* upregulation. BMP signalling, when activated, then coordinates cell fate proportions and invasion dynamics to drive collective cell invasion during vertebrate development.

## Results

### Ex vivo induction of cell migration in chick tissue explants

**Figure 1:**
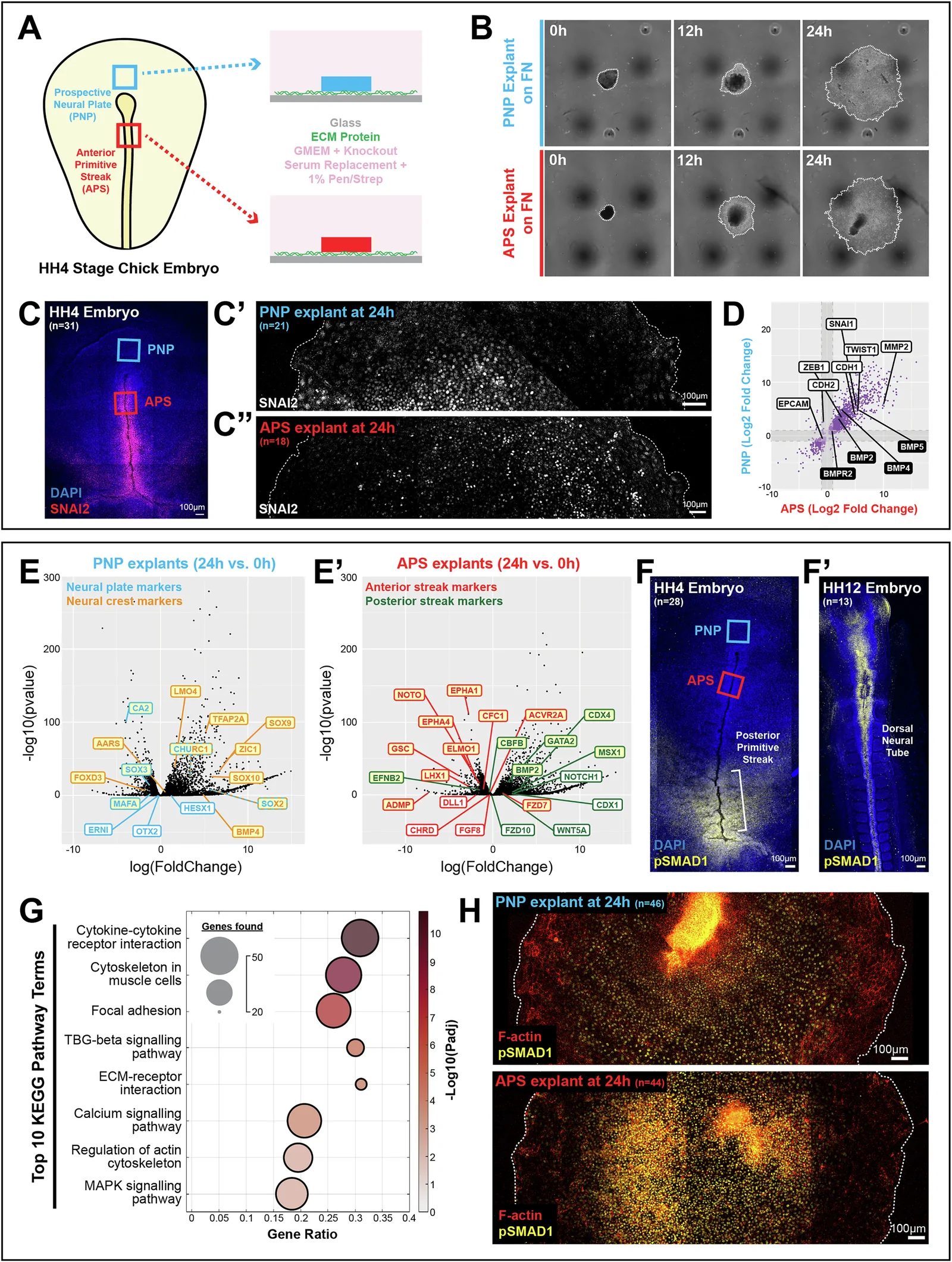
Ex vivo cell migration assay reveals BMP activation and BMP-induced cell fate changes. (**A**) Schematic of ex vivo EMT assay in chick explants. (**B**) Live imaging of migrating explants on fibronectin. (**C–C“**) Representative SNAI2 immunofluorescence in HH4 embryo (**C**), PNP explant after 24 h (**C’**), and APS explant after 24 h (**C“**). (**D**) Bulk RNA-seq fold change plot of EMT genes (white) and BMP signalling genes (black). Log2 of gene expression fold change in 24 h migrating explants compared to 0 h controls in APS tissue (x axis) and PNP tissue (y-axis). All *p* adj <0.01. Shaded: |fold change|<1. *n* = 16–27 explants, three biological replicates. (**E**–**E’**) Volcano plots of differentially expressed genes in PNP (E) and APS (**E’**) migrating explants at 24 h vs 0 h. Yellow highlight: *p* adj <0.01. (**F**–**F’**) Representative phospho-SMAD1 immunofluorescence in HH4 (**F**) and HH12 (**F’**) embryos. (**G**) Top eight terms enriched in gene ontology analysis of genes upregulated ≥2-fold in both PNP and APS explants upon migration. KEGG: Kyoto Encyclopaedia of Genes and Genomes. (**H**) Representative phospho-SMAD1 immunofluorescence and phalloidin staining in PNP (top) and APS (bottom) migrating explants after 24 h. Source data are available online for this figure.

We first asked whether plating tissue explants onto fibronectin-coated glass is sufficient to drive cell migration. To compare different initial EMT states, we compared two gastrula-stage (HH4) chick tissues. Firstly, the prospective neural plate (PNP) tissue, which is neuroectodermal and not fated to undergo EMT. Secondly, the anterior primitive streak (APS) tissue, which is mesodermal and already actively undergoing EMT in the HH4-stage embryo. Both PNP and APS explants were plated on human fibronectin-coated glass-bottomed dishes and incubated at 37 °C and 5.5% CO_2_ for 24 h (Fig. 1A). Live imaging revealed robust migration in both PNP and APS explants, with cells collectively dispersing radially from the explant edge and moving over the fibronectin substrate (Fig. 1B; Appendix Movie S1). This experimental system, therefore, provides a platform to determine the transcriptional events as cells initiate cell migration independently of other developmental cues.

**Figure EV1:**
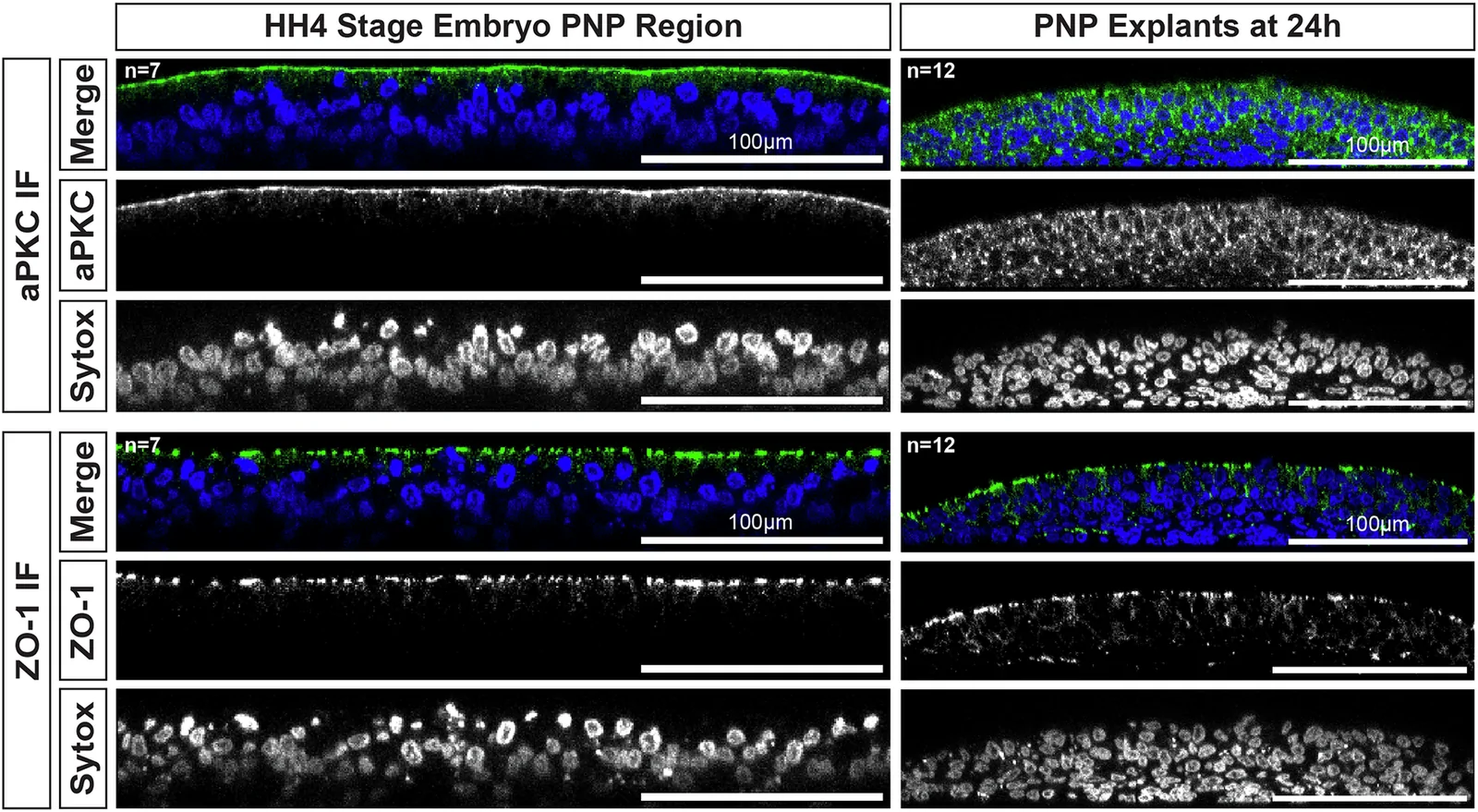
Loss of apical-basal polarity observed in PNP migrating explants. Reslices of representative confocal image of aPKC and ZO-1 immunofluorescence and SYTOX staining on the PNP embryonic region and PNP migrating explants on human fibronectin after 24 h of incubation.

In normal chick development, SNAI2, a key EMT driver known to be upstream of the EMT programme in both the chick mesoderm and neural crest (Nieto et al, 1994), is expressed in the primitive streak (Fig. 1C). Both PNP and APS migrating explants at 24 h of culture showed nuclear SNAI2 protein expression, indicating activation of the EMT transcriptional programme in PNP explants, in addition to maintained expression in APS explants (Fig. 1C’,C”). To further validate EMT induction, we examined epithelial polarity in PNP migrating explants. In vivo, the neuroepithelial PNP region displays classical apical-basal polarity with Zonula Occludens-1 (ZO-1) and atypical Protein Kinase C (aPKC) localised to the apical domains (Fig. EV1). After 24 h in culture, however, PNP migrating explants exhibited disorganised ZO-1 and aPKC localisation along the apical and basolateral domains (Fig. EV1), consistent with loss of epithelial apical-basal polarity during EMT transitions. Bulk RNA sequencing performed on PNP and APS explants at 0 and 24 h of culture (PNP-0 h, PNP-24 h, APS-0 h, APS-24 h) confirmed these morphological findings (Datasets EV1–3). Both PNP and APS explants at 24 h of culture showed upregulation of a common set of key EMT drivers (*SNAI1, ZEB1* and* TWIST1*) and a matrix metalloproteinase gene known to be implicated in EMT (Duong and Erickson, 2004), *MMP2* (Fig. 1D). These transcriptomic signatures confirmed the progression of EMT in both tissue types, demonstrating that this substrate-induced perturbation triggers a shared EMT programme across distinct germ layers.

### Ex vivo induction of cell migration drives convergent cell fate shifts toward migratory cell states

**Figure EV2:**
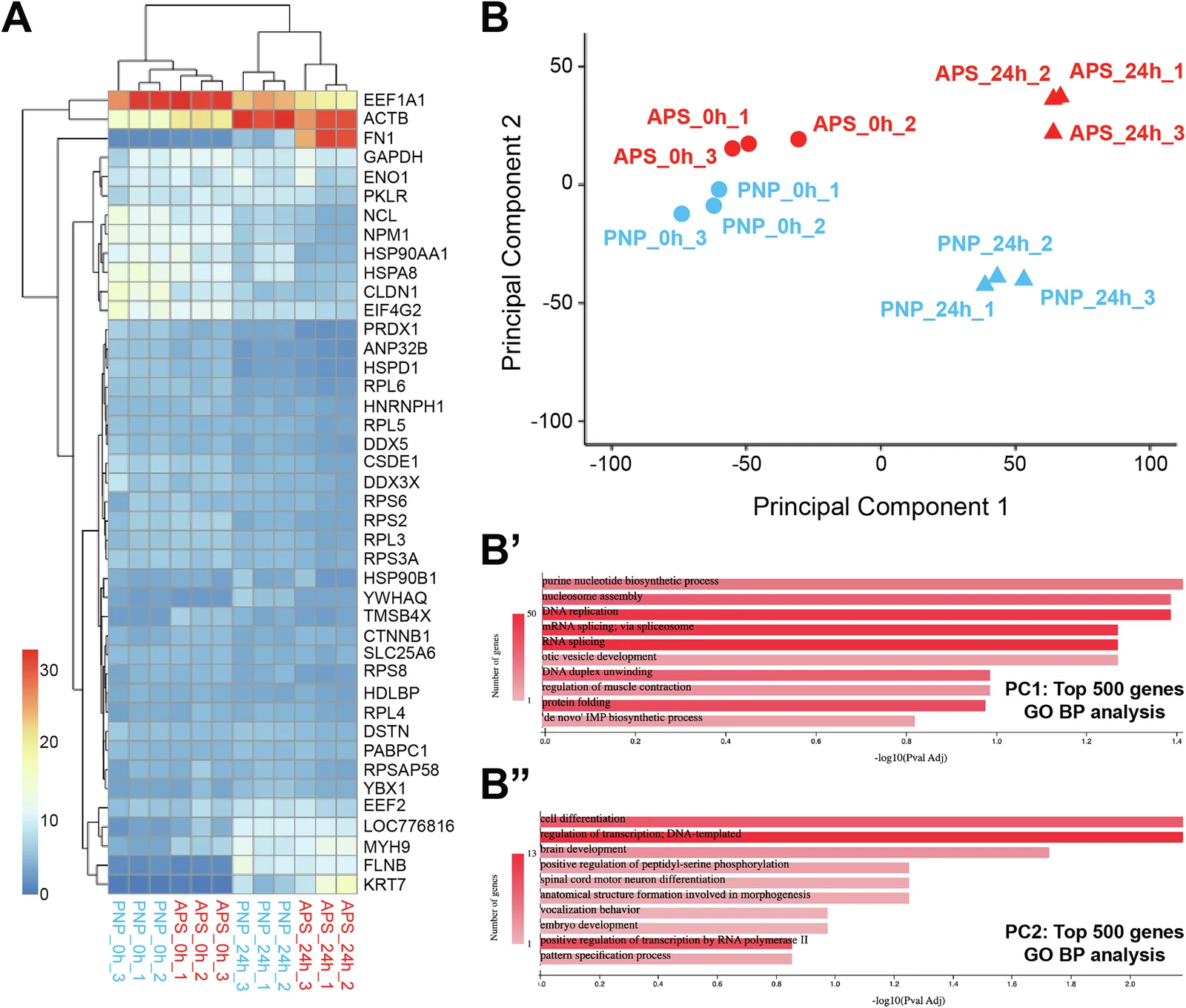
Bulk RNA sequencing reveals common genes upregulated or downregulated in migrating explants, regardless of tissue type. (**A**) Z-score heatmap showing the top differentially expressed genes across all samples, filtered by a summed z-score threshold (>50). Genes are hierarchically clustered, and samples are grouped by tissue type (PNP vs APS) and timepoint (24 vs 0 h). (**B**) Principal component analysis (PCA) of all bulk RNA sequencing samples reveals clear separation of samples at 24 vs 0 h along PC1 and PC2. PC1 accounts for variation primarily associated with DNA replication, transcription, and translation. PC2 corresponds to changes in cell differentiation status. (**B’**) Gene Ontology (GO) Biological Process (BP) enrichment of the top 500 genes contributing most to PC1, highlighting pathways associated with biosynthesis, translation, and cell cycle progression. (**B“**) GO BP enrichment of the top 500 genes contributing most to PC2, showing enrichment for terms related to differentiation and developmental processes.

Having established that our ex vivo system successfully induces EMT, we next explored the common transcriptional signatures associated with an induced migratory state. Global analysis of the transcriptomes revealed several key insights. A heatmap of the top differentially expressed genes across all four conditions showed consistent patterns, with the translation elongation gene *EEF1A1* expression enriched in 0-hour samples and the actin cytoskeletal gene *ACTB* expression in 24-hour samples, regardless of tissue type (Fig. EV2A). Notably, most genes showed coordinated changes regardless of tissue type; genes upregulated in APS migrating explants were also upregulated in PNP explants, and vice versa (Fig. 1D). This strong correlation suggested a shared migrating explant programme of gene expression changes. Principal component analysis confirmed this pattern, with the major axis of variation (PC1) segregating samples by timepoint (Fig. EV2B). Gene ontology analysis revealed that the top 500 genes contributing to PC1 were enriched for biosynthetic processes such as replication, transcription, and translation (Fig. EV2B’). Meanwhile, PC2 separated samples by tissue type with enrichment of differentiation-related terms (Fig. EV2B,B”). Notably, 24-h migrating samples showed greater divergence along PC2 than 0-hour samples (Fig. EV2B), suggesting that while induced cell migration drives a shared programme, distinct lineage identities continued to specify and diverge.

Critically, both tissues showed cell fate specification responses following EMT activation, with shifts toward more migratory fates within their respective germ layers. PNP explants downregulated neural plate markers and upregulated neural crest markers after 24 h in culture (Fig. 1E), suggesting a fate shift toward the migratory neural crest lineage. In parallel, APS explants showed downregulation of anterior primitive streak markers and upregulation of posterior streak markers (Fig. 1E’). Together, these findings indicate that substrate-induced cell migration triggers cell fate responses across germ layers.

### Ex vivo induction of cell migration triggers BMP pathway activation

We noticed that both neural crest and posterior primitive streak represent migratory cell fates within their respective germ layers, and both are known to be promoted by BMP signalling during normal development (Marchant et al, 1998; Mayor et al, 1995; Wilson et al, 1997; Neave et al, 1997; Nguyen et al, 1998; Glavic et al, 2004; Liem et al, 1995; Selleck et al, 1998; James and Schultheiss, 2005; Faure et al, 2002; Dale et al, 1992; Jones et al, 1992) (Fig. 1F,F’). To explore this further, we first examined how induced cell migration affected the expression of BMP pathway components. Comparison of shared patterns of gene expression changes in both PNP and APS explants after 24 h revealed upregulation of BMP ligand genes (*BMP2, BMP4, BMP5*) (Fig. 1D; Appendix Fig. S1B). Additionally, many ECM components and remodelling enzymes were upregulated (Appendix Fig. S1A), consistent with the migratory phenotype. Gene ontology analysis of genes upregulated at least 2-fold in both tissues confirmed enrichment for the TGFβ signalling pathway (Fig. 1G; Appendix Fig. S2), which includes the BMP signalling pathway. We focused specifically on BMP signalling due to the cell fate changes observed in both PNP and APS migrating explants (i.e. toward neural crest and posterior primitive streak fates, respectively) being consistent with known roles of BMP signalling rather than TGFβ signalling during chick development. We note that TGFβ signalling may also be active in this context, but its role in coordinating collective cell behaviours and fate specification falls outside the scope of the present study. To directly assess BMP signalling activity, we performed immunostaining for phosphorylated SMAD1 (pSMAD1), a well-established readout of active BMP signalling (Kretzschmar et al, 1997). Both PNP and APS migrating explants showed strong nuclear pSMAD1 signal (Fig. 1H), confirming pathway activation following substrate-induced cell migration. This BMP signalling activation in PNP and APS migrating explants was observed regardless of the ECM substrate used, occurring on both human fibronectin and rat-tail collagen I (Appendix Fig. S3).

**Figure EV3:**
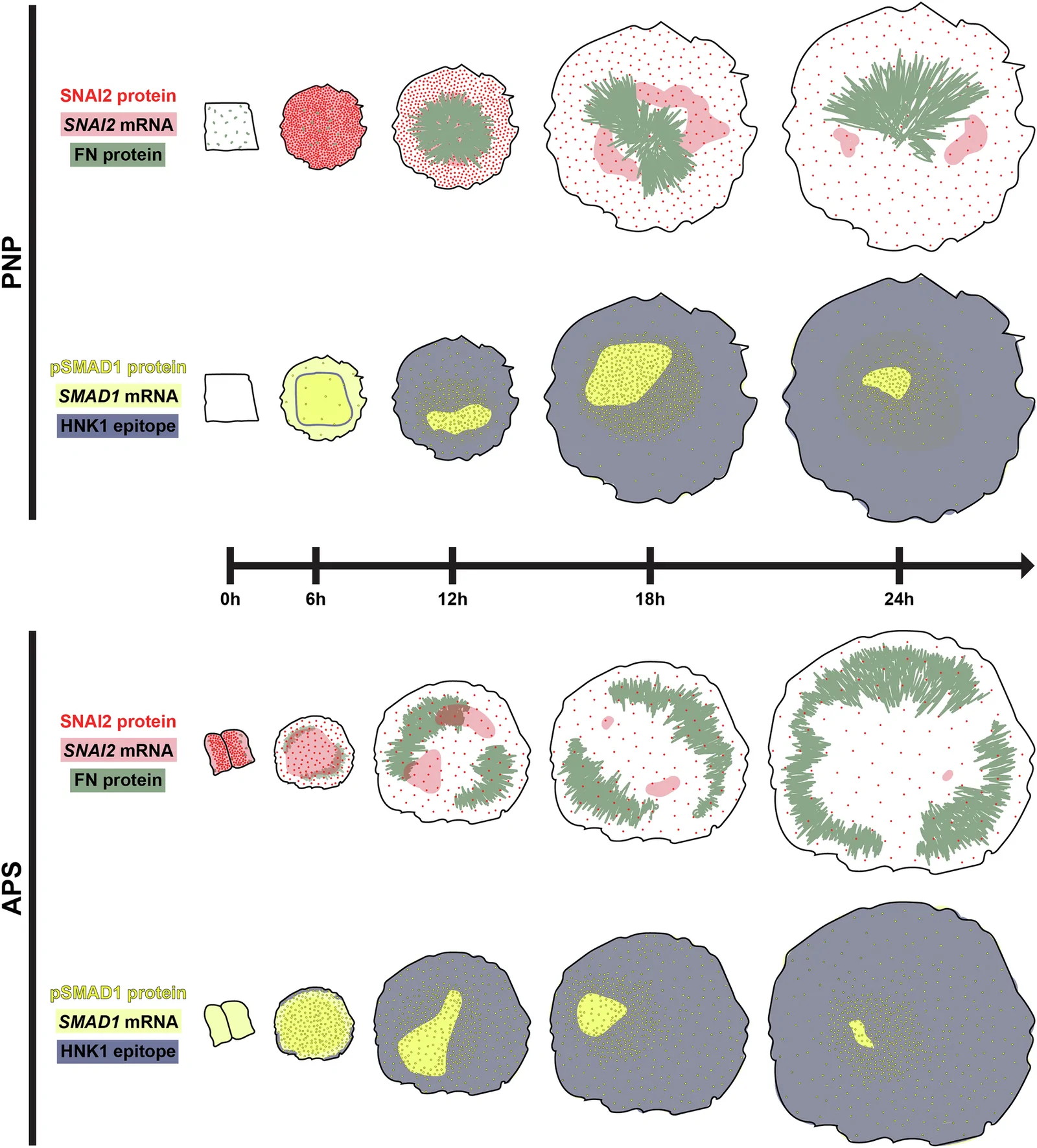
Summary of pseudo-time-course analysis of markers. Schematic of observed expression of SNAI2 protein, *SNAI2* mRNA, FN protein, pSMAD1 protein, *SMAD1* mRNA, and HNK1 epitope in PNP and APS migrating explants at multiple timepoints.

To directly characterise the temporal upregulation of SNAI2 and its relationship to BMP pathway activation in this system, we performed pseudo-time course analyses of PNP and APS explants fixed at 0, 6, 12, 18, and 24 h of culture on fibronectin and stained for *SNAI2* mRNA, SNAI2 protein, pSMAD1 protein, fibronectin (FN) protein, and the carbohydrate HNK1 epitope (summarised in Fig. EV3; see also Appendix Figs. S4–9). In PNP explants, both *SNAI2* mRNA and SNAI2 protein were absent at 0 h (Appendix Figs. S5 and S6), thus confirming the pre-EMT state of freshly dissected PNP tissue. *SNAI2* mRNA appeared broadly across the explant by 6 h of culture, before becoming progressively restricted to patches of expression as cells spread outward over subsequent timepoints (Appendix Fig. S6). SNAI2 protein also appeared robustly by 6 h of culture (Appendix Fig. S5). Together, this demonstrated the rapid induction of SNAI2 expression upon substrate contact. As for BMP signalling, pSMAD1 protein was absent at 0 h, sparse at 6 h, and enriched from 12 h onwards in central regions of PNP explants (Appendix Fig. S4). The mesenchymal markers FN protein and HNK1 epitope appeared from 6 h onwards and appeared particularly strong from 12 h onwards (Appendix Figs. S4 and S5). In APS explants, both *SNAI2* mRNA and SNAI2 protein were present at 0 h (Appendix Figs. S8 and S9), consistent with the endogenous EMT state of APS tissue. Similar to PNP explants, *SNAI2* mRNA expression became progressively restricted to patches with subsequent incubation in APS explants (Appendix Fig. S9). pSMAD1 protein was absent at 0 h, robust at 6 h, and enriched from 12 h onwards in central regions of APS explants (Appendix Fig. S7). Both FN protein and HNK1 epitope appeared from 6 h onwards (Appendix Figs. S7 and S8). A summary of the observed patterns of protein and mRNA expression in PNP and APS explants at different timepoints can be seen in Fig. EV3.

Together, these results demonstrate that substrate-induced cell migration triggers both BMP pathway component expression and signalling activity, consistent with the observed cell fate changes toward migratory lineages. Furthermore, the rapid upregulation of SNAI2 expression suggests a role for this transcription factor as an upstream activator of this switch.

### *SMAD1* upregulation couples EMT initiation to BMP signalling activity

**Figure 2:**
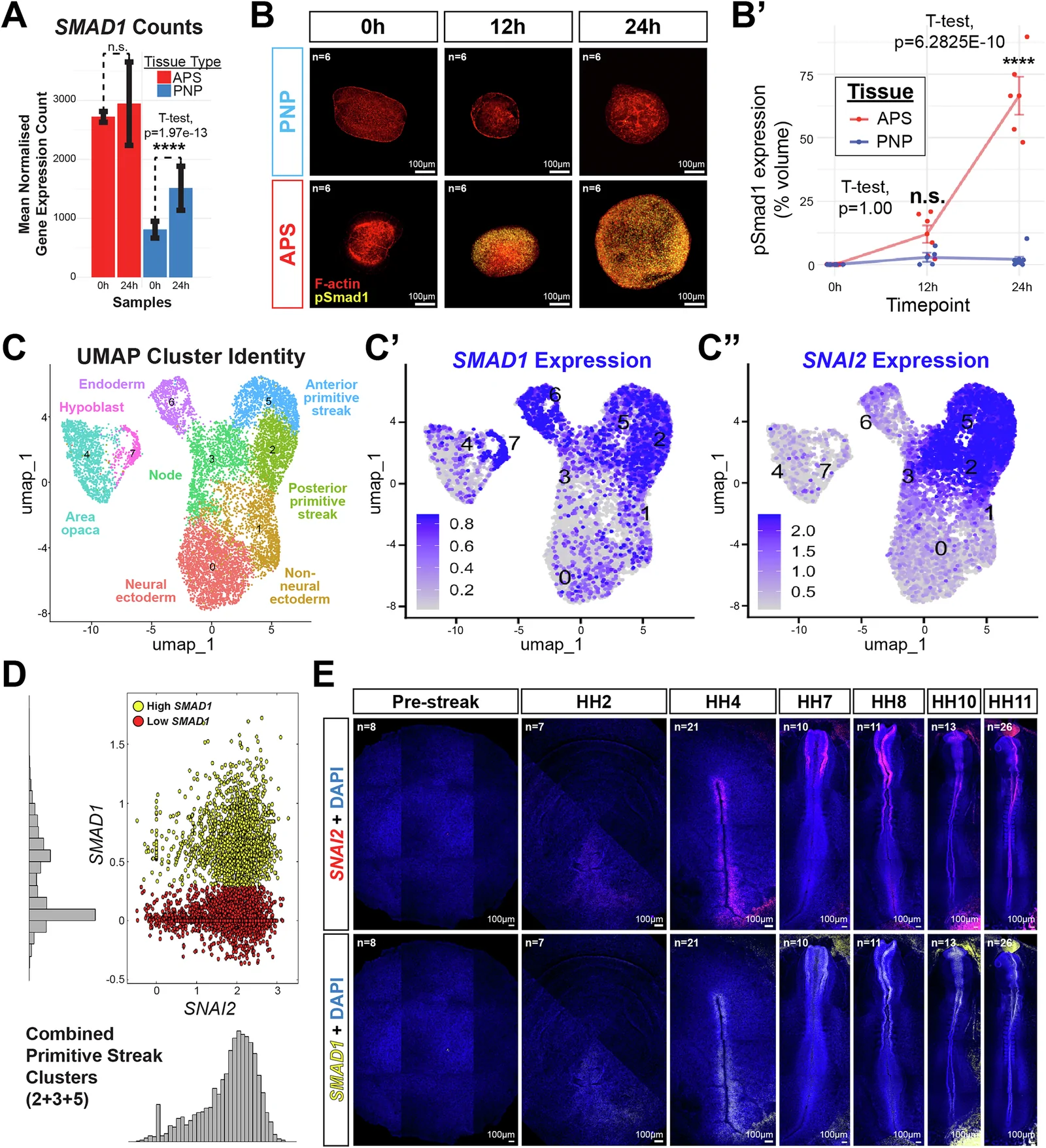
*SNAI2* and *SMAD1* expression correlate in vivo. (**A**) Bulk RNA-seq *SMAD1* expression in PNP and APS explants (0 vs 24 h). DESeq2 *p* value. (**B**) Representative pseudo-time course confocal images of phospho-SMAD1 immunofluorescence and F-actin stain in PNP and APS floating explants. (**B’**) Quantification of phospho-SMAD1 expression as percent volume of sytox signal in PNP and APS floating explants. (**C**–**C“**) scRNA-seq of gastrula-stage chick embryos. UMAP clusters (**C**), *SMAD1* expression (**C’**), *SNAI2* expression (**C“**). Sex and cell cycle regressed. Dim 1:12, res 0.30. (**D**) Distribution of *SMAD1* and *SNAI2* expression in a combined primitive streak cluster (clusters 2 + 3 + 5). Colour shows the bimodal distribution of *SMAD1* expression. Red: Cells with high *SNAI2* and low *SMAD1*. Yellow: Cells with high *SNAI2* and high *SMAD1*. (**E**) Representative *SNAI2* and *SMAD1* in situ HCR in whole embryos. Bleed-through was tested and controlled for by changing HCR hairpins. Source data are available online for this figure.

The observed BMP pathway activation following induced cell migration expression raised a key question: are these processes mechanistically linked? To investigate this, we searched our bulk RNA sequencing dataset for genes whose expression might control BMP signalling. We reasoned that genes linking EMT to BMP signalling should be upregulated only in PNP migrating explants, which undergo EMT induction, and not in APS explants, which maintain pre-existing EMT programmes. *SMAD1* mRNA was significantly upregulated in PNP explants after 24 h of migration compared to 0 h, consistent with BMP pathway activation following EMT induction (Fig. 2A). Pseudo-time course analysis of *SMAD1* mRNA expression in PNP explants further confirmed this: *SMAD1* mRNA was absent at 0 h in PNP explants and appeared from 6 h onwards, thus coinciding with the onset of *SNAI2* expression (Appendix Fig. S6). SMAD1 protein functions as an intracellular mediator of BMP signalling, without which cells cannot transduce BMP ligand signals to activate downstream target gene expression. The regulation of *SMAD1* expression, therefore, represents a mechanism for increasing cellular BMP activity during EMT, by conferring the capacity to transduce extracellular BMP ligand signals into nuclear transcriptional responses through intracellular effectors such as SMAD1.

Interestingly, *SMAD1* expression remained constitutively high in APS explants at both timepoints, suggesting that this tissue already possessed the molecular machinery for BMP signalling prior to explantation and migration (Fig. 2A). This was further confirmed using pseudo-time course analysis of *SMAD1* mRNA expression in APS explants (Appendix Fig. S9). As APS explants also upregulate BMP signalling in migrating explants, we asked whether this activation resulted from removing tissues from embryonic sources of BMP inhibition. During early chick development, BMP inhibitors such as CHRD are expressed and secreted from the Hensen’s node (Appendix Fig. S10) (Streit et al, 1998), raising the possibility that dissection alone might be sufficient to relieve BMP inhibition. To test this, we cultured explants in suspension for 24 h, thereby removing both ECM contact and the physical substrate required for migration while still isolating tissues from embryonic sources of BMP inhibitors. The viability of these floating explants was confirmed using active Caspase-3 immunostaining after 24 h of incubation (Appendix Fig. S11). Floating APS explants, which already express EMT factors in vivo, gained BMP signalling activity after 24 h (Fig. 2B,B’), consistent with relief from node-derived inhibition. In contrast, floating PNP explants lacked detectable pSMAD1 signal (Fig. 2B,B’), indicating that BMP activation in PNP explants requires more than the removal of exposure to endogenous BMP inhibitors. We reasoned that this is likely due to the induced *SMAD1* expression in PNP explants, suggesting that *SMAD1* transcription could act as a molecular link between EMT initiation and BMP signalling.

### Identification of *SNAI2* as a candidate regulator of *SMAD1* expression

To determine candidates for the control of *SMAD1* expression in vivo, we generated single-cell RNA sequencing data from gastrula-stage chick embryos. Pearson’s correlation analysis revealed the top genes positively correlated with *SMAD1* expression (Dataset EV4). Strikingly, among the highly correlated genes, *SNAI2* emerged as a compelling candidate, ranking 20th with a Pearson’s correlation coefficient of 0.26. Notably, *SNAI2* was the only well-established EMT transcription factor ranked highly on this list; other core EMT drivers showed substantially weaker correlations with *SMAD1* (*ZEB1*: rank 104, r = 0.14; *TWIST1*: rank 149, r = 0.11; *ZEB2*: rank 278, r = 0.07; *SNAI1*: rank 1261, r = 0.01). This correlation-based finding identified *SNAI2*, a well-characterised EMT driver in both chick mesoderm and neural crest (Nieto et al, 1994), as a data-driven candidate for upstream regulation of *SMAD1* expression during EMT initiation.

UMAP clustering identified distinct embryonic territories (Fig. 2C; Appendix Fig. S12; Datasets EV5–12), with overlapping expression of *SMAD1* and *SNAI2* transcripts specifically within the primitive streak clusters which correspond to precisely the embryonic regions of EMT initiation (Fig. 2C’,C”), defined here as embryonic territories where expression of key EMT transcriptional drivers have started to become enriched relative to surrounding tissues. Detailed correlation analysis within a combined primitive streak cluster (clusters 2 + 3 + 5) revealed two distinct cell populations: one expressing high *SNAI2* but low *SMAD1*, and another co-expressing both *SNAI2* and *SMAD1* at high levels (Fig. 2D). This bimodal distribution of *SMAD1* expression in *SNAI2*-expressing cells is consistent with a model in which *SNAI2* and *SMAD1* are co-expressed in cells undergoing EMT initiation, with a subset of *SNAI2*-expressing cells showing high *SMAD1* expression. A third cell population expressing low *SNAI2* and high *SMAD1* was observed in some other clusters (Appendix Fig. S13). To validate these findings, we performed multiplexed in situ hybridisation chain reaction (HCR) for *SNAI2* and *SMAD1* across chick embryonic development. These experiments confirmed spatio-temporal co-localisation of *SNAI2* and *SMAD1* transcripts in the primitive streak, neural plate border, neural folds, and dorsal neural tube regions (Fig. 2E) across development, consistent with the literature (Gont and Lough, 2000; Le Dréau et al, 2012; Acloque et al, 2011; Hardy et al, 2011; McLarren et al, 2003). To ensure that the observed co-localisation was not an artefact of HCR amplification, we also detected regions of differential expression at higher magnification (Appendix Fig. S14). This establishes that *SNAI2* and *SMAD1* co-expression is a consistent feature of embryonic regions of EMT initiation. Together, these findings demonstrate that *SNAI2* and *SMAD1* are co-expressed during development within regions of EMT initiation, consistent with *SNAI2* acting as a candidate upstream regulator of *SMAD1* expression.

### BMP signalling activity is restricted to regions of EMT initiation during development

**Figure 3:**
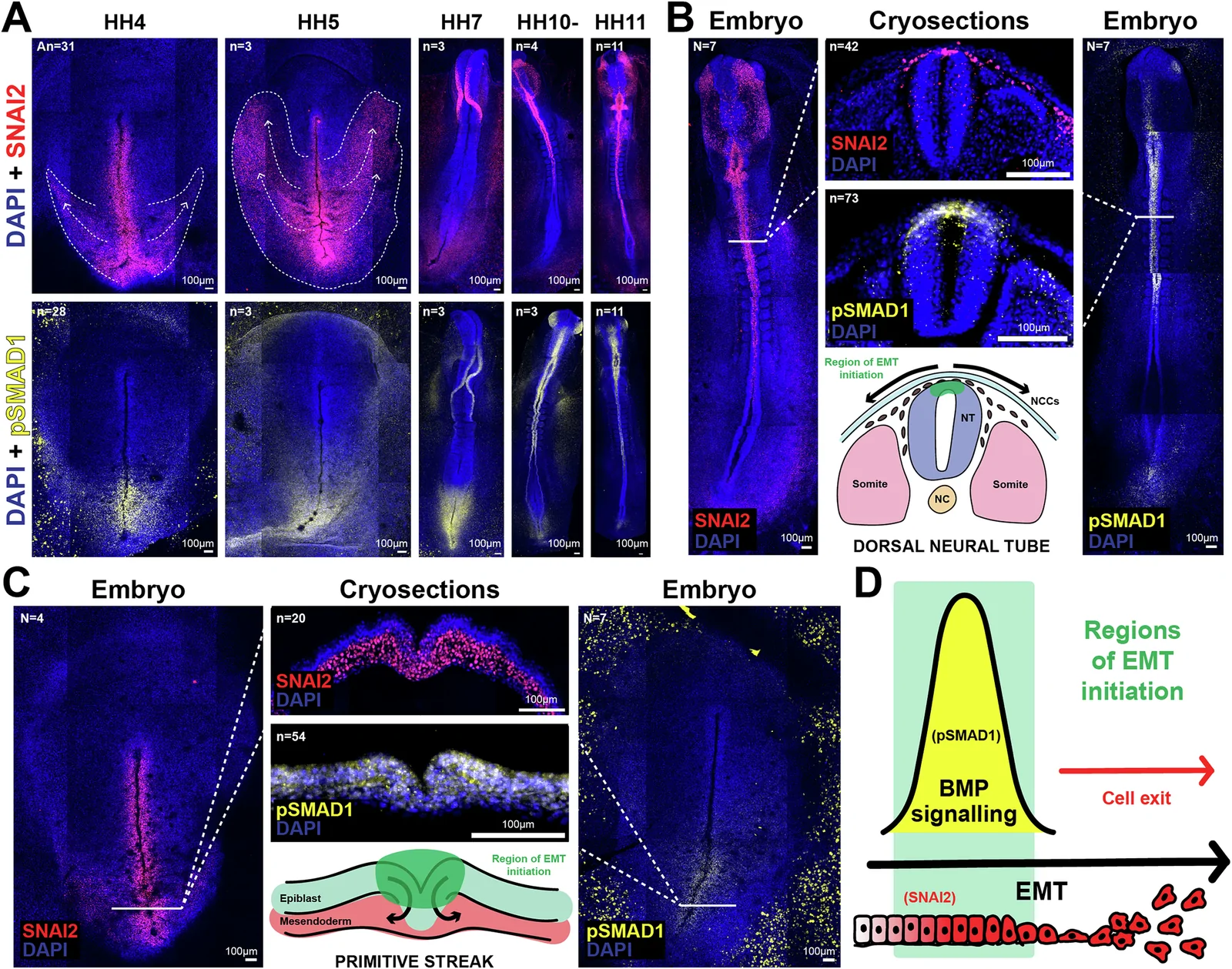
BMP signalling is restricted to regions of EMT initiation in vivo. (**A**) Representative SNAI2 and pSMAD1 immunofluorescence in whole embryos. Embryos were imaged from the epiblast side, but SNAI2 immunofluorescence signal from the migrating mesoderm layer can be seen, as indicated by the dashed white lines and arrows. HH4-stage SNAI2 immunofluorescence image is a re-display of the image from Fig. 1C. HH11 stage pSMAD1 immunofluorescence image is a re-display of the image from Fig. 1F’. (**B**) Representative SNAI2 and pSMAD1 immunofluorescence in HH10 embryos and cryosections during neural crest delamination. (**C**) Representative SNAI2 and pSMAD1 immunofluorescence in HH4 embryos and cryosections during mesoderm ingression. (**D**) Schematic of BMP signalling patterns coordinated with EMT progression. Source data are available online for this figure.

Having established that *SNAI2* and *SMAD1* transcripts co-localise at EMT initiation sites, we next asked whether BMP signalling activity itself shows similar spatial restriction. Interestingly, examination of pSMAD1 signal distribution in ex vivo PNP and APS migrating explants revealed that nuclear pSMAD1 signal was strongest in the centre of the explants and progressively reduced toward the periphery, where cells displayed more mesenchymal morphology (Appendix Fig. S15). This gradient pattern suggested that BMP signalling activity might be spatially restricted to regions of active EMT initiation rather than maintained throughout migration. To investigate this, we compared the spatial dynamics of SNAI2 and pSMAD1 expression across chick embryonic development from gastrulation through neural crest delamination. Immunostaining of SNAI2 in HH4 to HH11 chick embryos revealed nuclear signal in the primitive streak, migrating mesoderm, neural folds, dorsal neural tube, and migrating neural crest cells (Fig. 3A). In parallel, pSMAD1 immunostaining revealed nuclear signal in the posterior primitive streak, neural folds, and dorsal neural tube (Fig. 3A). Cryosectioning and confocal reslices revealed that SNAI2 signal initiates in the dorsal neural tube and persists in delaminated and migrating neural crest cells (Fig. 3B; Appendix Fig. S16). In contrast, the pSMAD1 signal was sharply restricted to the dorsal neural tube itself, with little to no signal detectable in the delaminated neural crest cells that had already begun migration (Fig. 3B; Appendix Fig. S16). Similarly, SNAI2 protein expression initiated in the primitive streak and persisted in migrating mesoderm cells (Fig. 3C), but pSMAD1 signal was restricted to the epiblast and primitive streak regions and was absent from migrating mesoderm cells (Fig. 3C). These observations confirm that EMT initiation and BMP signalling activity are tightly coupled in vivo, with BMP signalling restricted to regions of EMT initiation during normal development (Fig. 3D).

### *SNAI2* expression is sufficient to drive *SMAD1* expression in vivo

**Figure 4:**
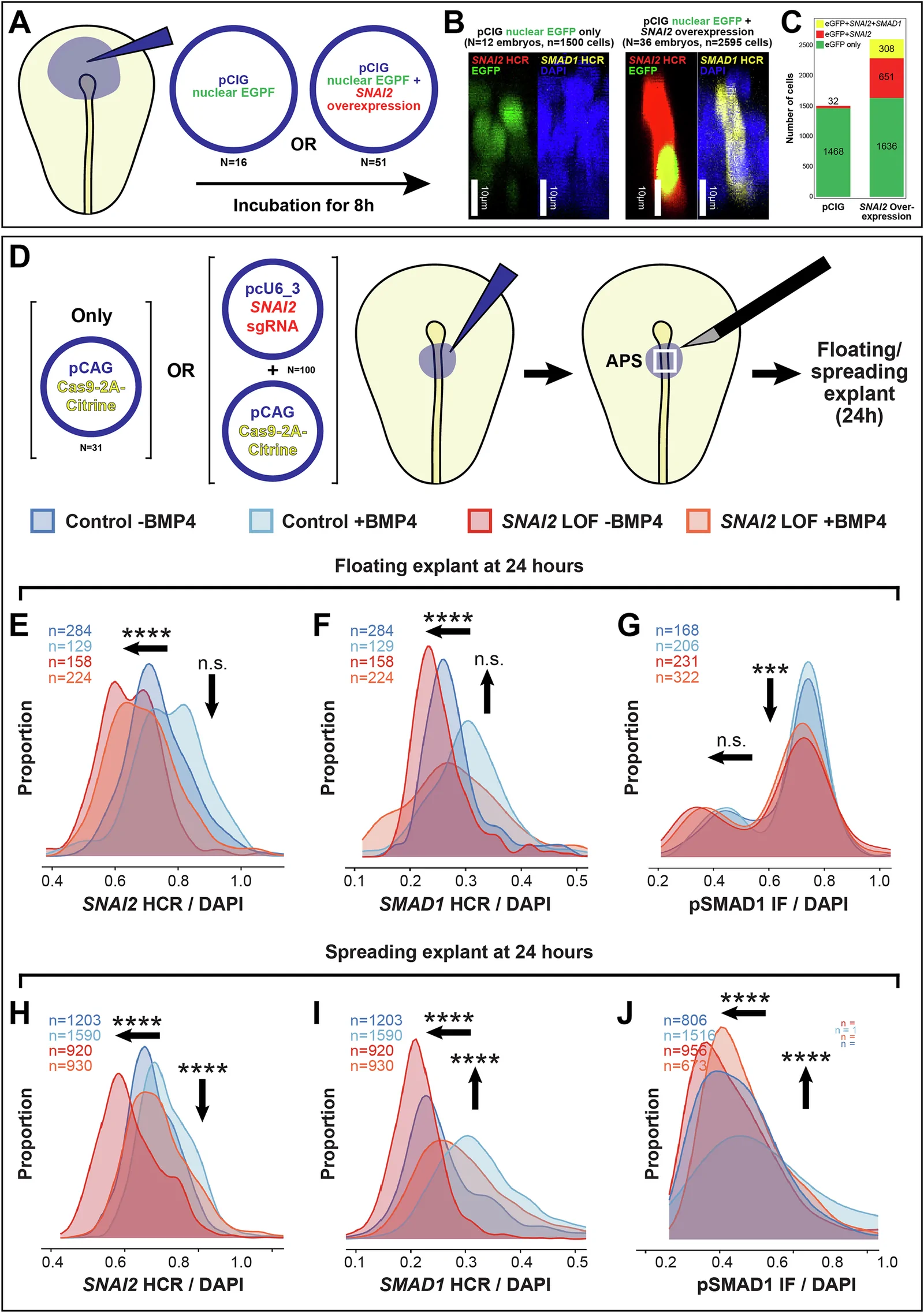
*SNAI2* overexpression is sufficient to drive *SMAD1* expression in vivo; *SNAI2* is required for *SMAD1* expression ex vivo. (**A**) Schematic of the *SNAI2* overexpression experiment in the HH4 prospective neural plate. Embryos were electroporated with pCIG (negative control) or pCIG-SNAI2 (*SNAI2* overexpression construct) and incubated for 8 h. (**B**) FIJI 3D view of representative *SNAI2* and *SMAD1* in situ HCR in pCIG vs pCIG-SNAI2 electroporated cells. (**C**) Cell counts. The *SNAI2*-positive cells in the pCIG controls are due to some node cells being electroporated. (**D**) Schematic of *SNAI2* CRISPR-KO experiment in free-floating and migrating explants. (**E**–**J**) Quantification of DAPI-normalised signal intensities in segmented mosaic crispant cells. Two independent features of each distribution were tested: horizontal shift (i.e., whether per-mask expression level differs between conditions, assessed by the Wilcoxon rank-sum test on the median) and vertical shift (i.e., whether the spread, or variance, of per-mask expression differs between conditions, assessed by Levene’s test). A significant vertical shift indicates a change in the heterogeneity of expression across the cell population. Horizontal shift: Wilcoxon, floating (pSMAD1 W = 25772, Z = 1.502, r = 0.072, *p* = 0.1333; *SNAI2* W = 20430, Z = 6.479, r = 0.345, *p* = 9.259e-11; *SMAD1* W = 21279, Z = 7.399, r = 0.394, *p* = 1.381e-13), spreading (pSMAD1 W = 775234, Z = 10.682, r = 0.222, *p* = 1.237e-26; *SNAI2* W = 4028541, Z = 35.668, r = 0.531, *p* = 1.244e-278; *SMAD1* W = 4051818, Z = 36.206, r = 0.539, *p* = 4.849e-287). Vertical shift: Levene’s test, floating (pSMAD1 F = 14.0630681, *p* = 0.0008027342; *SNAI2* F = 0.96920137, *p* = 0.9074221; *SMAD1* F = 1.0755198, *p* = 0.40055453), spreading (pSMAD1 F = 28.9718604, *p* = 1.616453e-07; *SNAI2* F = 17.239895, *p* = 6.710668e-05; *SMAD1* F = 311.49642, *p* = 7.317392e-67). The y-axis represents kernel density estimates normalised to a total area of 1 under the curve to allow comparison across groups of unequal size. Small n represents the number of Cirtine-positive masks analysed. The number of explants analysed were the following: floating control -BMP4 (*N* = 4 for *SNAI2/SMAD1* HCR, *N* = 4 for pSMAD1 immunofluorescence), floating control +BMP4 (*N* = 4 for *SNAI2/SMAD1* HCR, *N* = 4 for pSMAD1 immunofluorescence), floating *SNAI2* LOF -BMP4 (*N* = 3 for *SNAI2/SMAD1* HCR, *N* = 4 for pSMAD1 immunofluorescence), floating *SNAI2* LOF + BMP4 (*N* = 4 for *SNAI2/SMAD1* HCR, *N* = 4 for pSMAD1 immunofluorescence), spreading control -BMP4 (*N* = 4 for *SNAI2/SMAD1* HCR, *N* = 3 for pSMAD1 immunofluorescence), spreading control +BMP4 (*N* = 4 for *SNAI2/SMAD1* HCR, *N* = 4 for pSMAD1 immunofluorescence), spreading *SNAI2* LOF -BMP4 (*N* = 4 for *SNAI2/SMAD1* HCR, *N* = 4 for pSMAD1 immunofluorescence), spreading *SNAI2* LOF + BMP4 (*N* = 4 for *SNAI2/SMAD1* HCR, *N* = 3 for pSMAD1 immunofluorescence). Source data are available online for this figure.

**Figure EV4:**
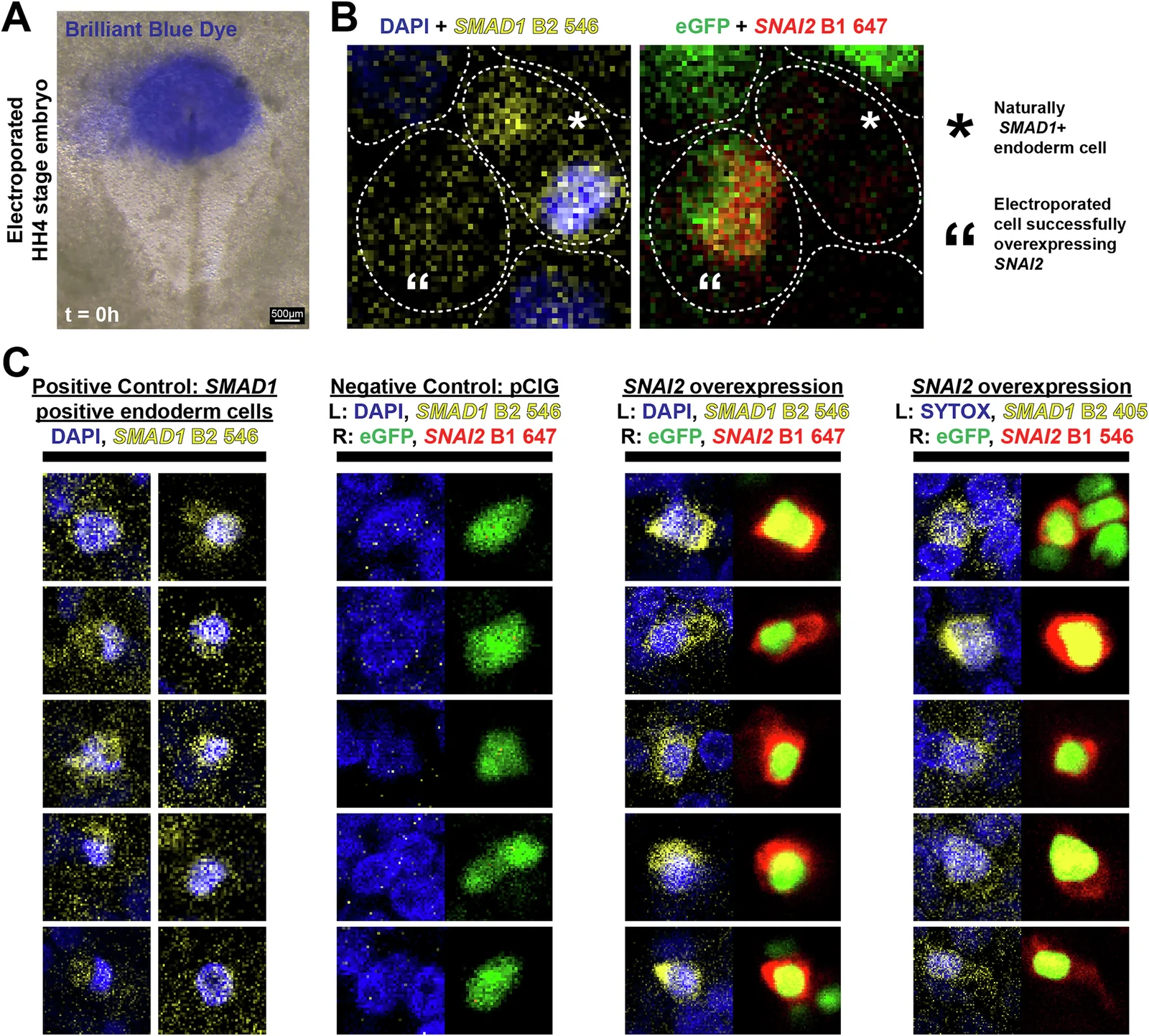
*SNAI2* overexpression is sufficient to drive *SMAD1* expression. (**A**) Representative brightfield image of an electroporated embryo at *t* = 0 h. (**B**) A representative confocal image showing a naturally *SMAD1*-positive endoderm cell next to an electroporated cell. (**C**) Representative confocal images of *SNAI2* and *SMAD1* in situ HCR staining of endoderm cells (left; positive control for *SMAD1* expression), pCIG-electroporated cells (middle; negative control for *SNAI2* expression), and cells electroporated with the *SNAI2*-overexpression construct (right two columns; experimental; different HCR hairpin combinations). Images were cropped from the original images for visualisation purposes.

We next asked whether SNAI2, as a transcription factor, might regulate *SMAD1* transcription. Although the Snail family of transcription factors canonically function as transcriptional repressors (reviewed in Nieto, 2002), there have been reports of these transcription factors acting as transcriptional activators (reviewed in de Herreros, 2024). To test whether *SNAI2* expression is sufficient to induce *SMAD1* expression, we performed targeted electroporation of an *SNAI2*-overexpression construct with nuclear EGFP reporter (pCIG-SNAI2) into the PNP region of HH4-stage chick embryos, a region that normally expresses neither *SNAI2* nor *SMAD1*. As a negative control, embryos were electroporated with the pCIG backbone alone, which expresses nuclear EGFP but lacks the *SNAI2* coding sequence (Fig. 4A). Constructs were co-injected with brilliant blue dye into the space between the epiblast and the vitelline membrane to visualise successful targeting (Fig. EV4A). After 8 h of incubation, we validated ectopic SNAI2 expression in EGFP-positive cells using SNAI2 immunofluorescence. While control embryos showed no detectable SNAI2 protein in the electroporated PNP region, embryos electroporated with pCIG-SNAI2 exhibited robust nuclear SNAI2 protein expression specifically in EGFP-expressing cells (Appendix Fig. S17A), confirming successful overexpression and nuclear localisation of the transcription factor. We further validated ectopic *SNAI2* mRNA expression using HCR staining (Appendix Fig. S17B). Imaging revealed that *SNAI2*-overexpressing cells remained within the prospective neural plate without undergoing ingression, consistent with previous reports (Moly et al, 2016). We then performed multiplexed in situ HCR for both *SNAI2* and *SMAD1* transcripts. As a positive control of *SMAD1* expression, endoderm cells (naturally *SNAI2*-negative and *SMAD1*-positive) were stained (Fig. EV4B,C). As a negative control, cells electroporated with the pCIG backbone were stained and showed no expression of either *SNAI2* or *SMAD1* in EGFP-positive cells within the electroporated PNP region (Figs. 4B,C and EV4C). In contrast, EGFP-positive cells electroporated with the pCIG-SNAI2 construct co-expressed both *SNAI2* and *SMAD1* (Figs. 4B,C and EV4C), demonstrating that ectopic *SNAI2* expression was sufficient to drive ectopic expression of *SMAD1*. Any potential fluorophore bleed-through was tested and controlled for by changing HCR hairpins (Fig. EV4C). Together, these results demonstrate that *SNAI2* overexpression is sufficient to drive *SMAD1* expression in vivo.

### *SNAI2* expression is required for *SMAD1* expression and BMP signalling activity ex vivo

To test whether *SNAI2* is required for *SMAD1* expression in vivo, we used CRISPR/Cas9-mediated gene editing to knock out *SNAI2* expression in regions where it is normally expressed. We cloned *SNAI2* single guide RNAs (sgRNA) into the pcU6-3 plasmid and co-electroporated these with the pCAG Cas9-2A-Citrine plasmid into the neural plate border region of HH4-stage embryos (Appendix Fig. S18C). The sgRNA was designed to target a DpnII restriction enzyme cut site within the *SNAI2* gene, such that successful CRISPR/Cas9 editing would disrupt these sites and prevent DpnII digestion (Appendix Fig. S18A). PCR amplification of the target sequence followed by DpnII digestion and gel electrophoresis confirmed effective gene editing (Appendix Fig. S18B). As a negative control, embryos were electroporated with the pCAG Cas9-2A-Citrine vector alone (Appendix Fig. S18C). Plasmids were co-electroporated with brilliant blue dye to ensure successful targeting (Appendix Fig. S18D), and embryos were incubated for 24 h to allow sufficient time for gene editing and subsequent transcriptional changes. Since only the pCAG Cas9-2A-Citrine vector contains a fluorescent reporter (Citrine), *SNAI2* knockout is necessarily mosaic even within Citrine-positive cells, as we cannot definitively identify which cells received both the pCAG Cas9-2A-Citrine plasmid and the pcU6-3 SNAI2-sgRNA plasmid. Despite this inherent variability, quantitative analysis using cell surface segmentation revealed measurable changes in the targeted cell populations. CRISPR-mutagenised embryos showed a significant decrease in SNAI2 protein levels in Citrine-positive cells compared to controls (Appendix Fig. S18E), confirming effective reduction of *SNAI2* expression. Similarly, *SNAI2* transcript levels were significantly reduced in the CRISPR-mutagenised embryos compared to controls (Appendix Fig. S18G), validating the gene editing approach at both protein and mRNA levels. However, despite this reduction in *SNAI2* expression, *SNAI2* knockout samples showed no significant changes in either pSMAD1 protein levels (Appendix Fig. S18F) or *SMAD1* transcript levels (Appendix Fig. S18H) compared to controls. We reasoned that this absence of effect may reflect the role of redundant mechanisms operating to regulate BMP activity in vivo. Indeed, pSMAD1 is active in a region of epiblast slightly extending that of SNAI2, suggesting that additional regulators of its expression operate in vivo (Fig. 3A).

To test this in a more controlled setting, we dissected APS explants from *SNAI2* CRISPR and control embryos and cultured these in either free-floating or fibronectin-plated conditions, treating each with or without exogenous BMP4 protein (Fig. 4D). The resulting explants were then either stained with multiplexed in situ HCR for *SNAI2* and *SMAD1* or immunostained for pSMAD1. This ex vivo approach allowed us to assess BMP response directly while controlling for the complexity of the intact embryonic environment. Here, like the in vivo experiment described above, Citrine-positive cells were segmented and used as a mask within which the HCR/immunofluorescence signals were quantified. Once we had the distribution of HCR/immunofluorescence signals, two shifts were analysed: (1) horizontal shift to ask if cells generally expressed more or less of the gene/protein of interest, and (2) vertical shift to ask if cells were more or less heterogeneous in the expression of the gene/protein of interest. In both free-floating and spreading conditions, *SNAI2* CRISPR explants showed a significant leftward (horizontal) shift in the distribution of *SNAI2* expression compared to controls, indicating a reduction in *SNAI2* expression and confirming CRISPR editing (Fig. 4E,H). *SNAI2* CRISPR cells also showed a similar leftward (horizontal) shift in the distribution of *SMAD1* expression in both free-floating (Fig. 4F) and spreading (Fig. 4I) conditions, indicating reduced *SMAD1* expression. In the spreading explants, this leftward (horizontal) shift was also accompanied by a significant upward (vertical) shift in the proportion of cells with lower *SMAD1* expression (Fig. 4I), indicating that cells had lower *SMAD1* expression and were less heterogeneous. At the level of BMP pathway activation, *SNAI2* CRISPR cells showed a significant leftward (horizontal) and upward (vertical) shift in the distribution of pSMAD1 expression (Fig. 4J), indicating lower BMP signalling pathway activation and lower heterogeny in crispant cells. In free-floating explants, *SNAI2* CRISPR cells showed a significant downward (vertical) shift in the distribution of pSMAD1 expression (Fig. 4G), indicating higher heterogeny. Notably, all of these results (i.e. reduction in *SNAI2, SMAD1* and pSMAD1 expression) in *SNAI2* CRISPR cells persisted even in the presence of exogenous BMP4 (Fig. 4E–J), indicating that *SNAI2* is required for *SMAD1* expression and BMP pathway activation regardless of BMP ligand availability. Together, these results demonstrate that *SNAI2* is required for *SMAD1* expression and BMP signalling activity ex vivo.

### BMP signalling is dispensable for EMT initiation but regulates cell fate proportions

**Figure 5:**
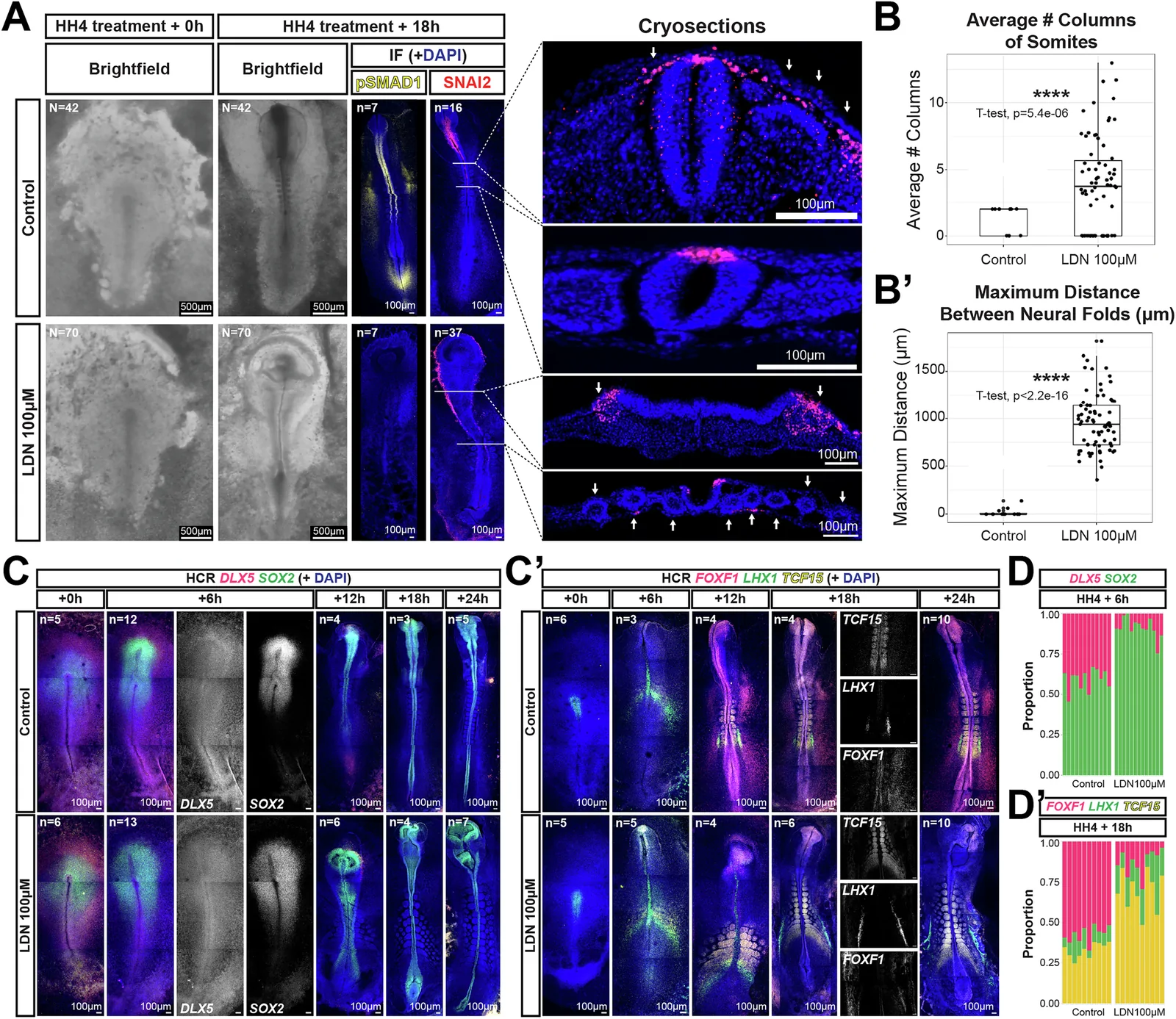
BMP signalling tunes cell fate proportions but not EMT initiation. (**A**) Representative brightfield and SNAI2/pSMAD1 immunofluorescence in control and LDN-treated embryos (left). Representative cryosections of SNAI2 immunofluorescence in control and LDN-treated embryos (right). In the top cryosection panel, arrows point to SNAI2-positive migrating neural crest cells. In the second-to-bottom cryosection panel, arrows point to SNAI2-positive cells migrating from the flat neural plate border region. In the bottom panel, arrows point to extra lateral somites. (**B**, **B’**) Quantification of somite columns (**B**) and neural fold distance (**B’**) in control vs LDN-treated embryos. For somite columns, WT should be 2 (1 column of somites on each side of the main body axis), unless development is delayed. For neural fold distance, WT should be 0 μm (successful neural tube closure), unless development is delayed. Box plots show median (centre line), 25th–75th percentiles (box bounds), and whiskers extending to the largest/smallest value within 1.5x the interquartile range; individual points beyond this range are plotted as outliers. (**C**,** C’**) Neural plate border marker *DLX5*+, neural plate marker *SOX2* (**C**) and lateral plate mesoderm marker *FOXF1* + intermediate mesoderm marker *LHX1* + paraxial mesoderm marker *TCF15* (**C’**) in situ HCR in control vs LDN-treated embryos at 0, 6, 12, 18, and 24 h of incubation. (**D**,** D’**) Quantification of proportional embryonic area positive for *DLX5* vs *SOX2* at 6 h (**D**) and *FOXF1* vs *LHX1* vs *TCF15* at 18 h (**D’**) in control vs LDN-treated embryos. Proportions were calculated relative to the total combined area of all measured domains (neural plate border + neural plate for (**D**); paraxial + intermediate + lateral plate mesoderm for **D’**). Source data are available online for this figure.

Having established that EMT activation induces BMP pathway competence and that *SNAI2* can drive *SMAD1* expression, we next investigated the functional consequences of this molecular coupling. We first treated whole HH4-stage chick embryos with the pan BMP type I receptor (ALK1/2/3/6) inhibitor LDN193189 (LDN), at 100 μM in EC culture agar-albumen and let the embryos grow overnight. As expected, BMP inhibition caused extensive neural tube closure defects, with embryos displaying persistently open neural plates (Fig. 5A). Quantification revealed two prominent LDN-induced phenotypes: a significant increase in lateral somite columns beyond the normal bilateral pair (Fig. 5B) and failure of neural fold convergence, with significantly increased distance between neural folds (Fig. 5B’). Cryosections confirmed the formation of additional somites lateral to the main body axis (Fig. 5A). These morphological defects demonstrate that BMP signalling is essential for coordinating the complex morphogenetic movements and patterning events that shape normal embryonic development.

**Figure EV5:**
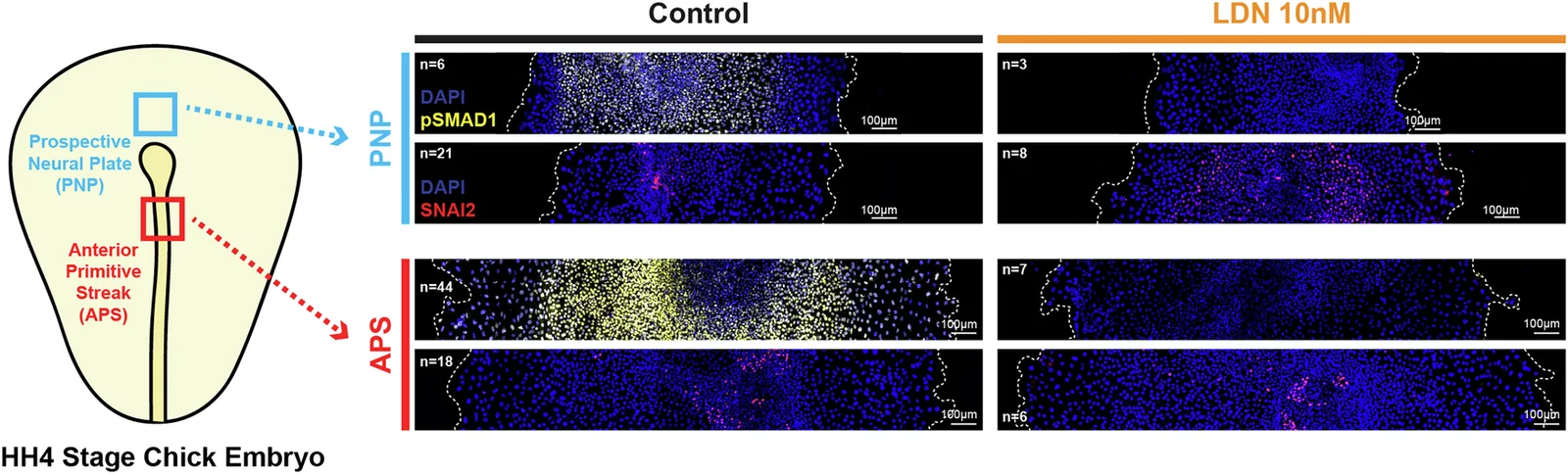
BMP signalling not needed for EMT initiation in ex vivo explants. Representative confocal images of pSMAD1 and SNAI2 immunofluorescence and SYTOX staining in control and LDN-treated PNP and APS explants on human fibronectin after 24 h of incubation.

Critically, LDN-treated embryos maintained robust SNAI2 protein expression in the neural plate border region despite complete loss of pSMAD1 signal (Fig. 5A), confirming that BMP signalling is dispensable for EMT initiation. Cryosections confirmed extensive migration of SNAI2-positive cells from the flattened neural plate border region (Fig. 5A), demonstrating that EMT can proceed even in the absence of BMP signalling and proper neural tube morphogenesis. Moreover, multiplexed in situ HCR revealed that LDN-treated embryos expressed *SNAI2, SMAD1*, dorsal neural tube marker *BMP4*, pre-migratory neural crest marker *FOXD3*, and migratory neural crest marker *SOX10*, all within the neural plate border region (Appendix Fig. S19A). This expression pattern persisted even when LDN treatment was performed earlier on HH3/3+ stage embryos (Appendix Fig. S19B), demonstrating that the EMT transcriptional programme can be activated independently of BMP signalling across multiple developmental timepoints. These findings are mirrored ex vivo, as 10 nM LDN treatment did not prevent the initiation of EMT in either PNP or APS tissue (Fig. EV5). This suggests that BMP signalling is not required for the onset of migration, but may instead function to regulate other aspects of cellular behaviour during cell invasion.

However, while EMT initiation proceeded normally, BMP inhibition significantly altered cell fate proportions in the embryo. By 6 h post-treatment, LDN-treated embryos showed reduced expression of the neural plate border marker *DLX5* and expanded expression of the neural plate marker *SOX2* (Fig. 5C,D), indicating a shift toward neural plate identity at the expense of neural plate border fate. Similarly, analysis of mesodermal markers revealed that BMP inhibition reduced the expression of lateral plate mesoderm marker *FOXF1* while expanding the expression of intermediate mesoderm marker *LHX1* and paraxial mesoderm marker *TCF15* by 18 h post-treatment (Fig. 5C’,D’). These cell fate shifts are consistent with known roles of BMP signalling in promoting lateral plate mesoderm and neural plate border specification during normal development. Together, these results demonstrate that BMP signalling is dispensable for EMT initiation but essential for regulating cell fate proportions, specifically promoting migratory lateral identities (neural plate border and lateral plate mesoderm) over more medial identities (neural plate and paraxial mesoderm).

### BMP signalling modulates cell invasion dynamics

**Figure 6:**
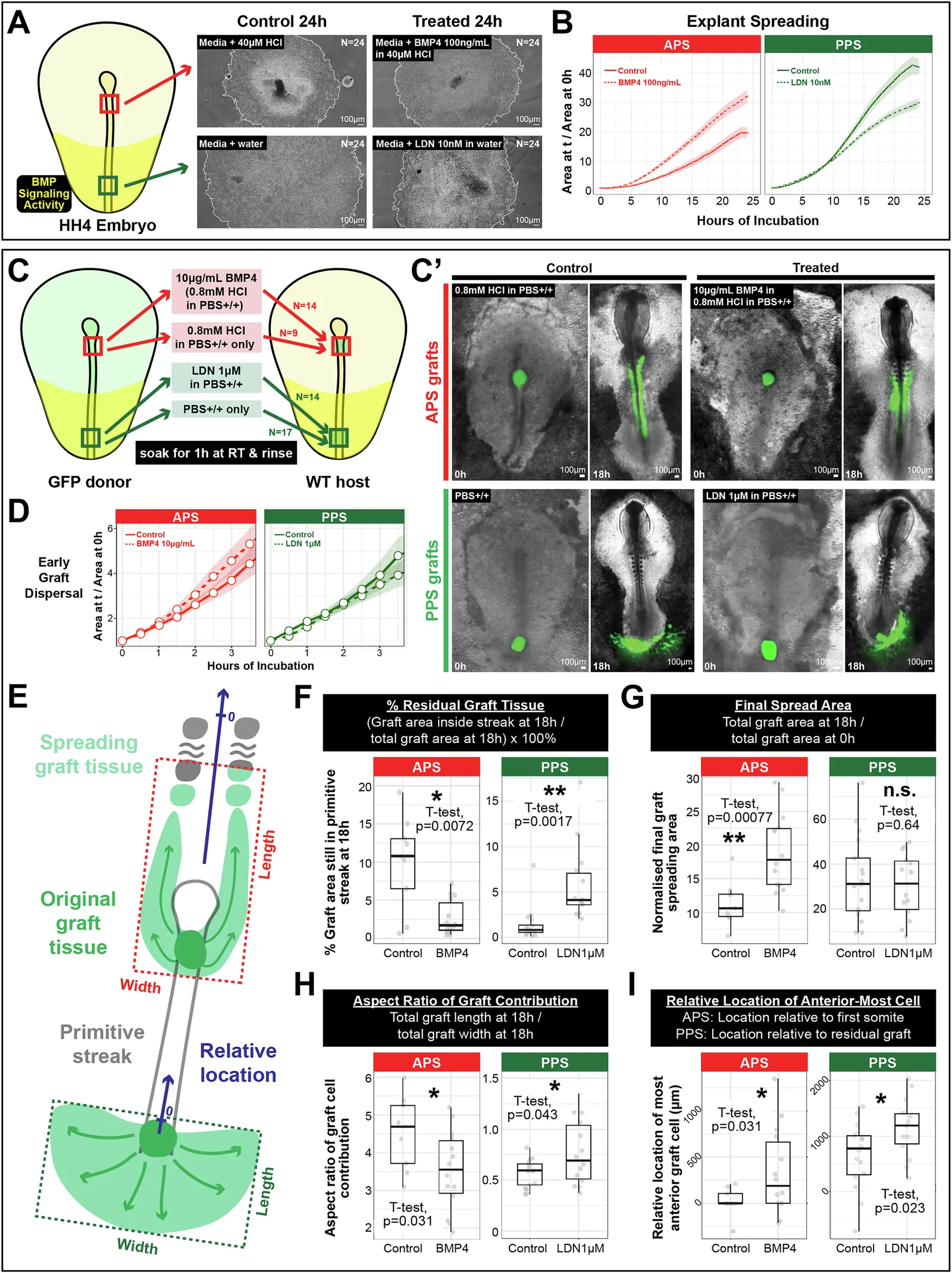
BMP signalling coordinates invasion dynamics. (**A**) Explant experiment schematic, representative images of control and treated APS and PPS explants. (**B**) Area quantification over time. (**C**,** C’**) Grafting experiment schematic (**C**) and representative snapshots of grafted embryos at 0 h and 18 h of incubation (**C’**). (**D**) Area quantification of early graft dispersal. (**E**) Schematic of different types of quantification performed on APS and PPS grafts. (**F**–**I**) Quantification of the percent of graft tissue remaining inside the primitive streak at end timepoint (**F**), the final graft spreading area normalised by the area at 0 h (**G**), the graft aspect ratio (length divided by width) at the end timepoint (**H**), and the degree of graft anterior migration, relative to the location of the first somite for APS grafts and to the location of the original graft location for PPS grafts (**I**). Box plots show median (centre line), 25th–75th percentiles (box bounds), and whiskers extending to the largest/smallest value within 1.5x the interquartile range; individual points beyond this range are plotted as outliers. Source data are available online for this figure.

Having established that BMP signalling is dispensable for EMT initiation, we next asked whether BMP signalling regulates collective cell invasion dynamics downstream of EMT. By dynamics, we specifically mean the directionality of migration, the rate at which tissues spread, and the rate at which precursor regions are depleted. To this end, we compared tissues already undergoing EMT with different endogenous BMP signalling states. Specifically, we compared APS tissue, which lacks BMP signalling, with the PPS tissue, which is BMP-positive. We first treated BMP-negative APS explants with recombinant mouse BMP4 protein (100 ng/ml) (Fig. 6A). Live imaging revealed that BMP4 treatment significantly enhanced APS explant spreading, with treated explants displaying faster initial dispersal, greater final controls (Fig. 6B; Appendix Fig. S20; Appendix Movie S2). Conversely, LDN treatment of BMP-positive PPS explants resulted in reduced spreading, with inhibited explants showing slower dispersal dynamics and less complete spreading (Fig. 6B; Appendix Fig. S20; Appendix Movie S2). These results demonstrate that BMP signalling directly modulates cell dispersal behaviour in tissues already undergoing EMT, supporting a downstream rather than initiating role for BMP signalling.

To validate these findings in an embryonic context, we performed in vivo grafting experiments. APS and PPS tissue were dissected from GFP donor embryos and soaked in either BMP4 (10 μg/mL) or LDN (1 μM) respectively for 1 h at RT, then grafted into WT host embryos and monitored by live imaging (Fig. 6C,C’). Consistent with our ex vivo results, BMP4-treated APS grafts showed accelerated dispersal during the first few hours of incubation (Fig. 6D). Furthermore, analysis of graft tissue retention within the primitive streak revealed that BMP signalling modulates the rate of precursor region depletion. BMP4-treated APS grafts showed significantly reduced retention of graft tissue within the primitive streak after 18 h of incubation (Fig. 6E,F), indicating faster depletion of the precursor region. However, the final spread area was similar between BMP4-treated and control APS grafts after 18 h of incubation (Fig. 6E,G). Analysis of graft contribution patterns revealed that BMP signalling also influences migration directionality. BMP4-treated APS grafts showed decreased aspect ratio (length/width) compared to controls (Fig. 6E,H), with significantly increased anterior migration (Appendix Fig. S21) and increased lateral migration (Appendix Fig. S21). This enhanced dispersal in both anterior and lateral directions is consistent with the specification of lateral mesoderm fates as well as faster exit from the primitive streak.

Conversely, LDN-treated PPS grafts showed initially slower cell dispersal during the first few hours of incubation (Fig. 6D). Analysis of PPS graft retention revealed that LDN treatment led to increased tissue retention in the primitive streak compared to controls (Fig. 6E,F), indicating slower precursor region depletion. The final spread area was similar between LDN-treated and control PPS grafts after 18 h of incubation (Fig. 6E,G). LDN-treated PPS grafts showed decreased aspect ratio compared to controls (Fig. 6E,H), with decreased anterior migration (Fig. 6E,I), consistent with the shift toward more embryonic mesodermal fates that contribute along a narrower anterior-posterior axis. Together, these in vivo grafting experiments demonstrate that BMP signalling modulates multiple aspects of collective cell invasion in tissues already undergoing EMT: it enhances cell dispersal rates, regulates precursor region depletion rates, and influences migration directionality.

Together, our findings reveal a reciprocal coupling mechanism where SNAI2 expression directly primes BMP pathway activity through *SMAD1* upregulation. When activated, BMP signalling in turn regulates cell fate proportions and invasion dynamics—including cell dispersal dynamics, precursor region depletion rates, and migration directionality. However, our findings are also consistent with known roles for BMP signalling in regulating cell fate specification, typically understood to be upstream of EMT initiation. By coupling the EMT gene-regulatory network (via *SNAI2* function) to BMP signalling (via *SMAD1* upregulation), we present a molecular mechanism for synchronising fate specification with EMT progression to coordinate tissue-level behaviours during collective cell invasion.

## Discussion

### EMT initiation triggers BMP pathway activation to coordinate collective cell invasion

**Figure 7:**
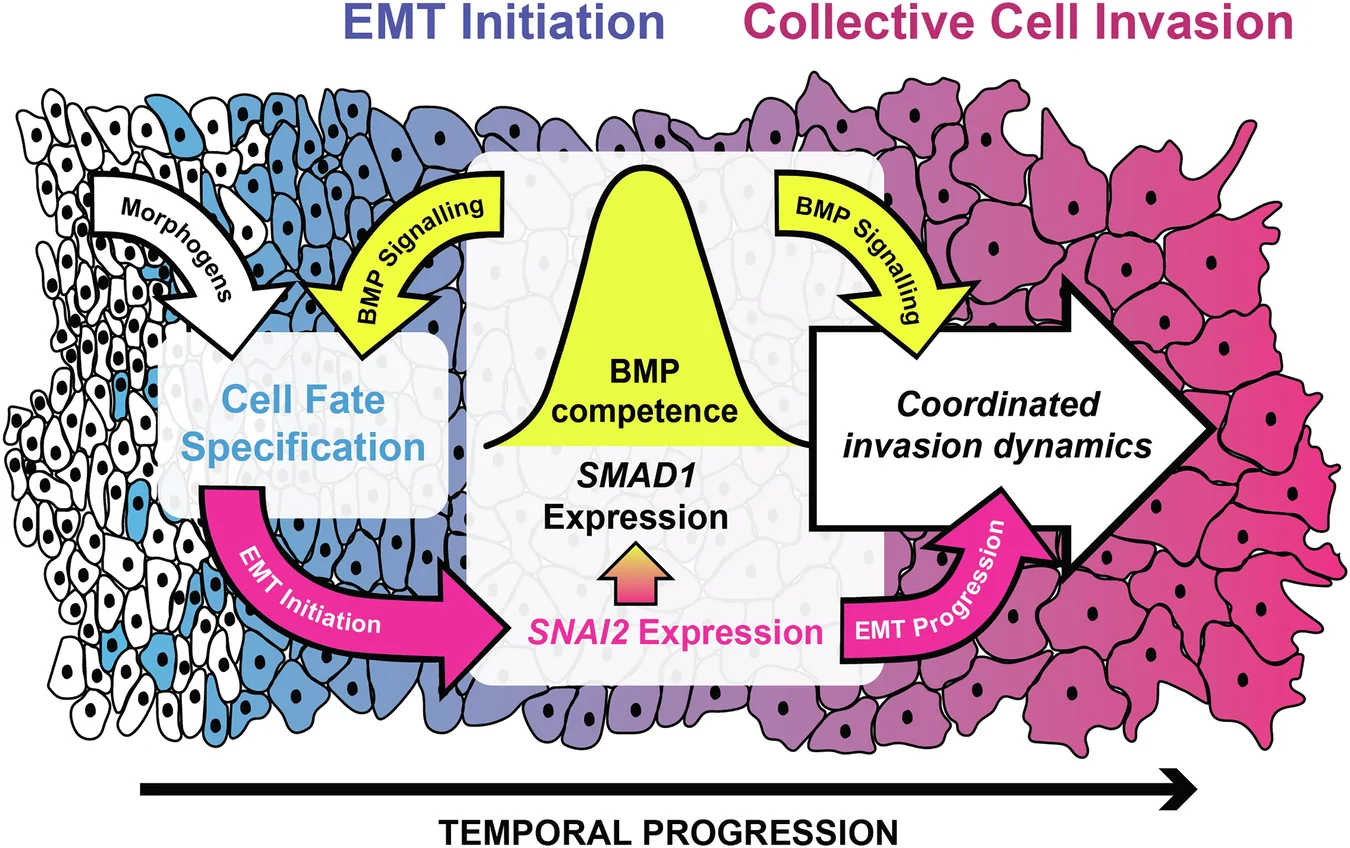
Summary of our working model for reciprocal interactions during collective cell invasion. Using explants of the primitive streak and posterior neural plate, we find that substrate-induced cell migration activates BMP signalling to induce migratory cell states, a key component of which is the transcriptional activation of SMAD1 expression by SNAI2. In vivo, this results in the spatio-temporal restriction of BMP activity to the sites of EMT initiation, where it acts to direct both cell fate and cell migration to drive collective cell invasion.

The traditional view of EMT positions morphogen signalling upstream of a linear cascade: morphogens first specify cell fates, which then trigger EMT regulators such as SNAI2, leading to cell invasion (reviewed in Acloque et al, 2009; Thiery et al, 2009; Leathers and Rogers, 2022; Zhang et al, 2025). However, to support the rapid and collective cell invasion that is observed in tissues such as the posterior primitive streak and cranial neural crest, cells must directly couple the specification of cells to these fates with the activation of the EMT regulators such as SNAI2. Our results provide a mechanism that is centred on the activation of SMAD1 expression by *SNAI2*, thereby activating BMP signalling levels at the site of EMT initiation (Fig. 7, orange arrow). BMP regulates cell fate specification upstream of *SNAI2* expression (Fig. 7, left yellow arrow), while driving a faster dispersion and migration of cells in vivo (Fig. 7, right yellow arrow). Subsequently, BMP activity is reduced as cells migrate, meaning that BMP-independent mechanisms continue to drive EMT progression as cells deposit and remodel their ECM during migration. Interestingly, BMP inhibition does not block induced cell migration in vitro, together suggesting that it is playing a role in setting the initial state of cells to prime them for faster dispersal within the endogenous environment. Determining the mechanisms downstream of BMP signalling in this process will be an important direction for future research.

By forcing cell migration ex vivo, we demonstrate that EMT initiation (marked by SNAI2 expression, loss of apical-basal polarity, upregulation of canonical mesenchymal markers, and transcriptional upregulation of core EMT drivers) triggers BMP pathway activation. This coupling operates in both neuroectodermal and mesodermal tissues, with substrate-induced EMT driving convergent cell fate changes toward migratory lineages in each germ layer: neural crest from prospective neural plate and posterior primitive streak from anterior primitive streak. The observation that these fate changes occur in both tissues despite their distinct developmental origins strongly suggests that EMT initiation provides a general mechanism for controlling BMP signalling. Critically, our floating explant experiments demonstrate that this priming is functionally meaningful: PNP tissue requires active EMT induction to activate BMP signalling, whereas APS tissue (which already expresses EMT factors) activates BMP signalling upon simple removal from embryonic BMP inhibitors. This demonstrates that tissue-specific BMP activity, established in-part through differential EMT states, determines whether cells can respond to changes in the morphogen environment. While APS and PNP explants share a common substrate-induced EMT programme, our pseudo-time course analysis reveals the progressive maturation of EMT states across both tissues, allowing us to begin to disentangle whether differences in collective invasion dynamics reflect EMT state, fate specification, or tissue geometry. These questions will be important to resolve fully in future work, particularly given the extensive literature demonstrating that partial and context-specific EMT states can produce distinct migratory behaviours (reviewed in Campbell and Casanova, 2016).

Single-cell RNA sequencing reveals co-localisation of *SNAI2* and *SMAD1* expression in regions of EMT initiation, with a bimodal distribution in which a subset of *SNAI2*-expressing cells co-express high levels of *SMAD1*. Our gain-of-function experiments establish that ectopic *SNAI2* expression is sufficient to drive *SMAD1* transcription in naive tissue, directly linking the key EMT driver to the expression of core BMP pathway components. Ex vivo loss-of-function experiments further demonstrate that *SNAI2* is required for *SMAD1* expression and BMP signalling, with *SNAI2* CRISPR cells showing significantly reduced *SMAD1* transcript levels and a dampened pSMAD1 response to exogenous BMP4 in both free-floating and fibronectin-plated conditions. We note that in vivo CRISPR experiments did not resolve this necessity relationship, likely reflecting the role of additional mechanisms impacting BMP activity in vivo.

A striking feature of this coupling is the sharp spatial and temporal restriction of BMP signalling activity to regions of EMT initiation. Both in vivo during neural crest delamination and mesoderm ingression and ex vivo in our migrating explants, pSMAD1 signal is highest in cells within the EMT initiation zone and progressively decreases in cells that have become mesenchymal. This spatial pattern reveals that BMP signalling functions as a transient coordinator during the transition from epithelial to migratory states, rather than as a sustained signal throughout migration. The restriction of BMP activity to EMT initiation zones may serve to temporally gate cell fate decisions to a specific developmental window, ensuring that fate specification occurs synchronously with EMT progression rather than persisting into later migratory phases.

Critically, BMP signalling proves dispensable for EMT initiation itself. LDN-mediated BMP inhibition does not prevent SNAI2 expression or the initiation of cell invasion in either ex vivo explants or in vivo embryos, establishing that EMT can proceed independently of BMP pathway activity. However, BMP signalling coordinates two critical processes: cell fate proportions and invasion dynamics. BMP inhibition shifts cell fates away from migratory lateral identities (neural plate border & lateral plate mesoderm) toward more medial identities (neural plate and paraxial mesoderm), consistent with the well-established role of BMP signalling in dorsoventral and mediolateral patterning. Additionally, BMP signalling modulates the tempo of cell dispersal, precursor region depletion, and migration directionality in tissues already undergoing EMT. This functional architecture, where *SNAI2* primes BMP signalling through *SMAD1* upregulation and activated BMP signalling coordinates fate specification and invasion dynamics, provides a mechanism for synchronising cellular state transitions with morphogen-driven patterning during collective cell invasion. While it remains unclear whether BMP signalling acts directly on migratory behaviour or indirectly through fate allocation (or both simultaneously), we suggest that attempting to resolve these as separable sequential events may itself be misleading. In the context of collective invasion, cell fate decision-making, EMT, and migration appear to be deeply coupled processes that together drive a coordinated tissue-level response, rather than components of a linear causal chain.

### Coordinating precursor region dynamics during chick axis elongation

The coupling between EMT and BMP signalling provides a molecular mechanism for one of the fundamental challenges of vertebrate axis elongation: how to simultaneously maintain precursor populations while generating the differentiated, migratory cells needed to extend the body axis. During chick development, posterior regions must continuously contribute cells to the elongating body while maintaining sufficient precursor populations for continued growth. The posterior primitive streak exemplifies this challenge, functioning as a precursor region that must balance maintenance with the production of extraembryonic and lateral mesodermal derivatives. Our findings suggest that BMP signalling modulates this balance by tuning the rate at which precursor cells transition from maintenance to differentiation and dispersal.

Our grafting experiments directly demonstrate this regulatory function. BMP4 treatment of anterior primitive streak tissue (which lacks endogenous BMP signalling and normally contributes to paraxial mesodermal derivatives) accelerates both cell dispersal and precursor region depletion. Treated grafts show reduced retention in the primitive streak after 18 h and enhanced contribution to anterior and lateral embryonic regions, effectively converting anterior streak behaviour to resemble posterior streak dynamics. Conversely, LDN treatment of posterior primitive streak tissue (which is naturally BMP-positive) slows initial dispersal and increases tissue retention in the primitive streak. This bidirectional modulation reveals that BMP signalling actively tunes the tempo of precursor region exit rather than simply permitting it to occur, functioning as a temporal regulator that sets the pace of tissue remodelling during axis elongation.

Notably, the APS undergoes extensive EMT to contribute to paraxial mesoderm despite lacking endogenous BMP signalling, raising the question of what drives its collective behaviour in this context. Interestingly, APS cells at this stage are beginning to slow their ingression rates, consistent with previously described dynamics of precursor region maintenance in the anterior streak (Iimura and Pourquié, 2006; Guillot et al, 2021; Busby et al, 2024). Alternative signalling pathways, including FGF and Wnt, which are known to be active in the primitive streak at this stage (reviewed in Chuai and Weijer, 2009), are strong candidates for coordinating collective cell behaviour in the absence of BMP signalling activity, and represent an interesting avenue for future investigation.

The role of BMP signalling in specifying cell fates along the neural plate border (reviewed in Thawani and Groves, 2020; Groves and LaBonne, 2014; Pla and Monsoro-Burq, 2018; Prasad et al, 2019) and within mesodermal tissues (reviewed in Kishigami and Mishina, 2005) is well established, but our findings reveal how this pathway is integrated with EMT dynamics to coordinate collective cell behaviours at the tissue level. Consistent with known roles in morphogenesis and patterning (Castranio and Mishina, 2009; Eom et al, 2011; Ybot-Gonzalez et al, 2007; Tonegawa and Takahashi, 1998; Dias et al, 2014), BMP inhibition with LDN leads to neural tube closure defects and ectopic lateral somite formation. Importantly, the persistence of SNAI2 expression and cell invasion from the neural plate border region in LDN-treated embryos demonstrates that individual cells retain the capacity to undergo EMT in the absence of BMP activity. However, without BMP-mediated coordination of fate specification and invasion dynamics, these EMT events become decoupled from tissue-scale organisation: the neural plate remains open with cells dispersing from flattened folds, and mesodermal precursors produce excess paraxial mesoderm at the expense of lateral plate derivatives. These phenotypes illustrate that BMP signalling does not control whether cells undergo EMT, but rather ensures that the timing, directionality, and fate outcomes of migrating cells are coordinated with ongoing tissue morphogenesis.

### Implications for EMT-morphogen coordination in evolution and disease

The reciprocal coupling between EMT initiation and BMP signalling that we describe establishes a previously unrecognised dimension of EMT function. Beyond its well-characterised roles in breaking down epithelial architecture (e.g. loss of apical-basal polarity, ECM remodelling, cytoskeletal reorganisation, and altered cell-cell adhesion), EMT now emerges as an active regulator of morphogen signalling competence. By tuning cellular responsiveness to patterning signals, this additional EMT function provides a mechanism for coordinating migration with fate specification in ways that would be difficult to achieve if these processes were regulated independently.

From an evolutionary perspective, coupling EMT to signalling may represent an important vertebrate innovation for coordinating individual cell behaviours with collective tissue movements. While both EMT machinery and morphogen signalling pathways are ancient and broadly conserved across metazoans (Shook and Keller, 2003; Pérez-Pomares and Muñoz-Chápuli, 2002; Mosby et al, 2024), direct coupling between these processes could have enabled fine-tuning of invasion timing and coordination, ensuring that precursor region exit and fate specification occurred in concert with tissue-scale movements. The conservation of this coupling across germ layers in the chick embryo suggests it may represent a general developmental principle, though comparative studies across vertebrate species will be needed to assess its evolutionary origins and diversification.

Beyond development, reciprocal interactions between EMT and signalling may be relevant for understanding pathological contexts where EMT drives disease progression, particularly cancer metastasis and fibrosis. If similar coupling mechanisms operate in these contexts, therapeutic strategies targeting the reciprocal interactions between EMT state and morphogen responsiveness (rather than either process in isolation) might offer more effective approaches for disrupting pathological invasion. Understanding whether analogous EMT-morphogen couplings exist in other developmental and disease contexts will be important for assessing the generality of this coordination mechanism and its potential as a therapeutic target.

## Methods

**Table:** Reagents and tools table

Reagent/resource	Reference or source	Identifier or catalogue number
**Experimental models**		
Standard shaver brown fertilised chicken eggs		Medeggs Ltd., Norfolk
Transgenic chicken eggs		Roslin Institute, Edinburgh
**Recombinant DNA**		
pCAG Cas9-2A-Citrine	Williams et al, 2018	Addgene #92358
pcU6-3 sgRNA	Williams et al, 2018	Addgene #92359
**Antibodies**		
Anti-slug (C19G7) monoclonal antibody		#9585 (Cell Signalling Technology)
Anti-Smad1 (phospho S463 + S465) antibody		EPR20662-20 (Abcam)
Anti-chicken HNK1 monoclonal antibody		3H5 (DSHB)
Anti-ZO-1 monoclonal antibody		ZO-1-1A12 (Thermo Fisher Scientific)
Anti-PKC zeta monoclonal antibody		sc-17781 (Santa Cruz Biotechnology)
Purified Rabbit Anti-Active		570524 (BD Pharmingen™)
Caspase-3		B3/D6 (DSHB)
**Oligonucleotides and other sequence-based reagents**		
**Chemicals, enzymes and other reagents**		
LDN193189 hydrochloride		SML0559 (Sigma-Aldrich)
Recombinant Mouse BMP4 Protein		5020-BP-010/CF (RD Systems)
PBS + /+		Sigma-Aldrich
KnockOutTM Serum Replacement		Thermo Fisher Scientific
Penicillin-Streptomycin		Thermo Fisher Scientific
GMEM		Gibco
Native Human Fibronectin protein		AB80021 (Abcam)
Corning® Collagen I rat-tail high protein		354249 (Corning)
BactoAgar		Sigma-Aldrich
PFA		Sigma-Aldrich
TRIzolTM		Invitrogen
GlycoBlue		Thermo Fisher Scientific
GelRed® Nucleic Acid Gel Stain		Biotium
QuBit RNA High Sensitivity kit		Thermo Fisher Scientific
NEBNext Poly(A) mRNA Magnetic Isolation Module		New England Biolabs
NEBNext Ultra II Directional RNA Library Prep Kit for Illumina		New England Biolabs
SPRIselect magnetic beads		Beckman Coulter
High Sensitivity DNA Chips and Reagents		Agilent Technologies
trypsin		Thermo Fisher Scientific
BSA		Sigma-Aldrich
DMSO		Sigma-Aldrich
Triton X-100		Thermo Fisher Scientific
normal goat serum		Sigma-Aldrich
Tween 20		Sigma-Aldrich
Proteinase K		Thermo Fisher Scientific
5×SSCT		Thermo Fisher Scientific
probe hybridisation buffer		Molecular Instruments
HCR wash buffer		Molecular Instruments
amplification buffer		Molecular Instruments
VECTASHIELD® Antifade Mounting Medium		Vector Laboratories
sucrose		Thermo Fisher Scientific
EprediaTM Neg-50TM Frozen Section Medium		Thermo Fisher Scientific
PureLinkTM Genomic DNA Mini Kit		InvitrogenTM
**Software**		
CHOPCHOP	Labun et al, 2019	
Galaxy web platform	Afgan et al, 2016	
FastQC	Simon Andrews et al, 2010	
Trim Galore!	Krueger et al, 2023	
HISAT 2	Kim et al, 2015	
SAMtools markdup	Li et al, 2009	
StringTie	Pertea et al, 2015	
DESeq2	Love et al, 2014	
GeneCodis 4	Garcia-Moreno et al, 2022	
Seurat	Satija et al, 2015	
**Other**		

### Methods and protocols

#### Experimental embryology

##### Fertilised chicken eggs

Standard shaver brown fertilised chicken eggs (Medeggs Ltd., Norfolk) and all transgenic chicken eggs (Roslin Institute, Edinburgh) were stored at 18 °C or room temperature (RT) until use and incubated at 37.5 °C in a humidified incubator (Thermo Fisher Scientific). Depending on the target embryonic stage (Hamburger and Hamilton, 1951), incubation times were specified.

##### Dissecting embryos

Egg shells were removed using blunt forceps, the vitelline membranes just surrounding the embryos were cut using micro-dissecting scissors, and embryos were removed from the egg and transferred into a petri dish with PBS+/+ (Sigma-Aldrich) or saline using a spoon. Under a dissecting microscope (Olympus SZX12), excess yolk on top of the embryos was washed off using glass pasteur pipettes and vitelline membranes were separated from the embryos using sharp forceps. Embryos were visually staged (Hamburger and Hamilton, 1951).

##### Migrating explants

Migrating explants were prepared as follows (based on methods in Busby et al, 2024). Glass-bottomed dish (MatTek; 35 mm Petri dish with 10 mm microwell; No. 1.5 coverglass 0.16–0.19 mm) microwells were coated with ECM protein of choice (see table below) in PBS+/+ for at least 1 h at 37.5 °C. After removing the ECM protein in PBS+/+ with a pipette, the glass-bottom dishes were left to thoroughly dry at 37.5 °C. All ECM-coated dishes were briefly washed with PBS+/+ twice prior to use. When the embryos were finished being dissected, the periphery of the glass-bottom dish wells were covered with silicone grease. About 200 μL of warm chick culture media, composed of 10% KnockOutTM Serum Replacement (Thermo Fisher Scientific) + 1% Penicillin-Streptomycin (Thermo Fisher Scientific) + GMEM (Gibco), was added directly to the wells.

**Table:** 

ECM Protein	Coating amount	Identifier
Native Human Fibronectin protein	2.5 μg	AB80021 (Abcam)
Corning® Collagen I rat-tail high protein	2.5 μg	354249 (Corning)

Embryonic regions of interest were dissected using a microlance needle (BD) and transferred to the wells using a pipette. Explanted chick tissues were oriented ventral side down and gently flattened using an eyelash knife. Glass coverslips were placed on top to cover the well. Dishes were flooded with PBS+/+ or chick culture media and wrapped with parafilm to prevent dehydration. Explants were either incubated in a tissue culture incubator or live imaged every hour using a widefield microscope (Nikon Eclipse Ti) at 37.5 °C and 5.5% CO_2_ for up to 24 h.

##### Free-floating explants

Free-floating explants were prepared as follows (based on methods in Streit and Stern, 1999). Embryonic regions of interest were dissected using a microlance needle (BD) and transferred into a 24-well plate Millicell® Cell Culture Inserts (Merck) with chick culture media. Plates were incubated in a tissue culture incubator at 37.5 °C and 5.5% CO_2_ for up to 24 h.

##### Embryo culture

Embryos were cultured using a modified protocol, based on EC culture methods (Chapman et al, 2001). EC culture plates were prepared by dissolving 0.3 g BactoAgar (Sigma-Aldrich) in 50 mL of autoclaved 145 mM NaCl by heating to boiling, followed by the addition of 50 mL prewarmed thin albumen and penicillin/streptomycin (1:100). The solution was stirred until homogeneous, and 1 mL was dispensed into each sterile 35 mm Petri dish. Plates were cooled at RT and stored at 4 °C until use. Embryos were transferred to autoclaved filter paper rings and mounted on EC culture. Cultures were placed in a humidified chamber at 37.5 °C for incubation.

##### Small molecular inhibitor treatment on embryos and explants

For whole embryos, inhibitors were added directly to the EC culture agar-albumen. For explants, inhibitors were added directly to the chick culture medium. Inhibitors were used according to the concentration in the table below.

**Table:** 

Inhibitor	Working concentration	Identifier
LDN193189 hydrochloride	10 nM explants, 100 μM embryos	SML0559 (Sigma-Aldrich)

##### Electroporation

Ex ovo electroporation was performed on chick embryos using an EC culture-based protocol. Embryos were prepared in EC culture and gently cleaned with saline using a mini-pastette (Alpha Laboratories) to remove excess yolk. A plasmid solution was prepared by diluting plasmid DNA (working concentration 1–3 μg/μL) in saline with 0.1% Brilliant Blue dye. Electroporation was conducted using an Intracel TSS20 Ovodyne Electroporator. Embryos were transferred to a custom electroporation chamber filled with sterile saline and positioned over the electrode. The plasmid solution was microinjected into the space between the epiblast and the vitelline membrane using an aspirator tube (Sigma-Aldrich) and pulled 100 mm borosilicate capillary tubes with filament (Harvard apparatus). Electroporation parameters for early chick embryos were set to 9.4 V, 3 pulses, 50 ms duration, and 490 ms intervals. Following electroporation, embryos were returned to their EC culture plates and incubated at 37.5 °C. Plasmid construct expression was typically detectable within 5–6 h by fluorescence microscopy or 2–3 h by confocal microscopy.

##### Tissue grafting

Grafting experiments were performed using HH4-stage cytosolic GFP donor embryos and wild-type host embryos. Desired donor tissue were dissected in PBS+/+ and soaked in either recombinant protein or inhibitor solution for 1 h at RT (see table below). Grafts were briefly washed in PBS+/+ after inhibitor or protein treatment prior to grafting.

**Table:** 

Inhibitor/protein	Working concentration	Identifier
LDN193189 hydrochloride	1 μM	SML0559 (Sigma-Aldrich)
Recombinant Mouse BMP4 Protein	10 μg/mL	5020-BP-010/CF (RD Systems)

Host embryos were cultured in EC culture and prepared for grafting by cleaning with saline and surgically removing a small region of tissue using an eyelash knife. A plastic ring (cut from an Eppendorf tube) was placed around each host embryo, and PBS+/+ was added inside. Treated grafts were transferred using a pipette and carefully placed over the exposed host region using a fluorescent dissecting microscope (Leica). Treated grafts were positioned into the host embryo using an eyelash knife, and excess PBS+/+ was slowly removed while continually monitoring graft position.

##### Tissue fixation

Whole embryos and tissue explants were fixed in 4% PFA (Sigma-Aldrich) in PBS−/− at RT for 2–3 h or at 4 °C overnight.

#### Molecular biology

##### Bulk RNA sequencing: Sample collection

Embryonic and explant tissues (*N*, biological replicate = 3) from 3–4 dozen embryos each were collected in ice-cold PBS+/+.

##### Bulk RNA sequencing: Extraction and integrity

Total RNA was extracted using TRIzol^TM^ (Invitrogen) following phase separation and isopropanol precipitation. Samples stored in 1 mL TRIzol^TM^ at −80 °C were thawed on ice and homogenised using a cell pestle. Chloroform (200 μL per 1 mL TRIzol^TM^) was added, and samples were mixed and incubated at RT for 3 min. Following centrifugation at 12,000×*g* for 15 min at 4 °C, the clear aqueous phase was carefully collected, avoiding the interphase, and transferred to a fresh tube. To precipitate RNA, 0.5 μL GlycoBlue (Thermo Fisher Scientific) and 500 μL isopropanol were added to each sample. After 10 min at RT, samples were centrifuged at 12,000×*g* for 10 min at 4 °C. The resulting RNA pellet was washed with 1 mL of 75% ethanol, vortexed briefly, and centrifuged at 7500×*g* for 5 min. Ethanol was removed, and pellets were air-dried for 10 min before being resuspended in 20 μL RNase-free water and incubated at 55 °C for 10 min. RNA integrity was assessed by agarose gel electrophoresis. Approximately 300–500 ng of each sample was mixed with loading dye and resolved on a 1% agarose gel in 1× TAE containing GelRed® Nucleic Acid Gel Stain (Biotium). Electrophoresis was carried out at 100 V for 1 h. Integrity was verified by the presence of distinct 28S and 18S rRNA bands with minimal smearing. Total RNA concentration was measured using the QuBit RNA High Sensitivity kit (Thermo Fisher Scientific), and samples were stored at −80 °C until further processing.

##### Bulk RNA sequencing: Library prep & sequencing

Polyadenylated mRNA was enriched from 1 μg of total RNA using the NEBNext Poly(A) mRNA Magnetic Isolation Module (New England Biolabs), following the manufacturer’s guidelines. Libraries were constructed using the NEBNext Ultra II Directional RNA Library Prep Kit for Illumina (New England Biolabs). Dual indexing was performed with NEBNext Multiplex Oligos for Illumina Sets 1 and 2 (New England Biolabs). Cleanup steps throughout library preparation were carried out using SPRIselect magnetic beads (Beckman Coulter). Library quality and fragment size distribution were assessed using an Agilent 2100 Bioanalyzer with High Sensitivity DNA Chips and Reagents (Agilent Technologies). Sequencing was conducted on an Illumina NextSeq 2000 platform (P3 flow cell, single-end 50 bp reads, roughly 90 million reads per sample) by Cambridge Genomic Services.

##### Single-cell RNA sequencing

HH3/3+ embryos were first placed in New culture (New, 1955). Control embryos used in this study were collected as part of a larger node ablation experiment. Embryos were incubated for 0.5, 2.5, or 5 h following manipulation. Only unablated control embryos were used here; these were detached from the vitelline membrane using an eyelash knife and reattached, to match handling conditions with ablated samples. Embryos were dissected in cold PBS+/+, and approximately half of the area opaca was trimmed to enrich for embryonic tissue. Each embryo was transferred to a 1.5 mL Eppendorf tube, washed 3× with PBS+/+, and incubated in 1 mL 0.05% trypsin (Thermo Fisher Scientific) at 37 °C for 15 min with gentle trituration every 5 min. Trypsin was quenched with 100 μL FBS, and cells were pelleted (450×*g*, 5 min, 4 °C) and resuspended in PBS + 0.05% BSA (Sigma-Aldrich). Cell viability (85–95%) was assessed via a haemocytometer. Single-cell suspensions were submitted to the CRUK CI Genomics Core Facility for 10X Genomics library preparation and Illumina sequencing.

##### Immunofluorescence

Chick embryos and tissue explants were stained following a standard whole-mount immunofluorescence protocol. All washes were performed in PBDT, composed of 1% DMSO (Sigma-Aldrich) + 1% BSA + 1% Triton X-100 (Thermo Fisher Scientific) in PBS−/−, unless stated otherwise. Embryos were blocked for at least 1 h at RT in blocking buffer, composed of 2% normal goat serum (Sigma-Aldrich) in PBDT, on a shaker. Primary antibodies were diluted in blocking buffer (see table below), and samples were incubated at 4 °C overnight with gentle agitation. The next day, samples were washed six times for 20 min at RT, then incubated overnight at 4 °C in secondary antibody (1:1000) and stains (e.g. DAPI, phalloidin, sytox, all 1:1000) diluted in blocking buffer. Unbound antibody and stains were removed by six 20 min washes at RT. Samples were stored in PBDT at 4 °C in the dark until mounting for imaging.

**Table:** 

Antibody	Dilution	Identifier
Anti-slug (C19G7) monoclonal antibody	1:1000	#9585 (Cell Signalling Technology)
Anti-Smad1 (phospho S463 + S465) antibody	1:1000	EPR20662-20 (Abcam)
Anti-chicken HNK1 monoclonal antibody	1:50	3H5 (DSHB)
Anti-ZO-1 monoclonal antibody	1:200	ZO-1-1A12 (Thermo Fisher Scientific)
Anti-PKC zeta monoclonal antibody	1:200	sc-17781 (Santa Cruz Biotechnology)
Purified Rabbit Anti-Active Caspase-3	1:500	570524 (BD Pharmingen™)
Mouse anti-avian fibronectin monoclonal antibody	1:20	B3/D6 (DSHB)

##### In situ hybridisation chain reaction

Fixed samples were washed using PBST, composed of 0.1% Tween 20 (Sigma-Aldrich) in PBS+/+. For long-term storage, samples were dehydrated by a graded series of 20 min methanol washes (PBST twice, 25% methanol in PBST, 50% methanol in PBST, 75% methanol in PBST, 100% methanol) and stored at −20 °C. Dehydrated samples were rehydrated in the reverse stepwise manner. In Situ detection of mRNA was performed using third-generation hybridisation chain reaction (HCR v3.0; Choi et al, 2018). For whole embryos, Proteinase K digestion was carried out by incubating samples in 10 μg/mL Proteinase K (Thermo Fisher Scientific) in PBST for either 3 min (stages HH8 and younger) or 5 min (stages HH8 and older) at RT. Samples were then post-fixed in 4% PFA in PBS−/− for 20 min at RT. Proteinase K digestion and post-fixation were omitted for explant samples. All samples were washed with PBST for 5 min twice, followed by a 5 min wash in 5×SSCT (Thermo Fisher Scientific; diluted in ultrapure water) at RT. Samples were then incubated at 37 °C for 30 min in probe hybridisation buffer (Molecular Instruments) for pre-hybridisation. To prepare the probe solution, 2 μL of the 1 μM stock of each probe set was added per 500 μL of hybridisation buffer, and samples were incubated at 37 °C overnight. On the following day, samples were washed four times in prewarmed HCR wash buffer (Molecular Instruments) at 37 °C for 15 min, then washed twice in 5×SSCT at RT for 5 min. To equilibrate, samples were incubated in amplification buffer (Molecular Instruments) at RT for at least 5 min. Hairpins were snap-cooled individually by heating 30 pmol of each (from a 3 μM stock) to 95 °C for 90 s in a thermocycler and allowing them to cool to RT in the dark. Equal volumes of snap-cooled hairpins H1 and H2 were combined in amplification buffer, and samples were incubated at RT overnight, protected from light. On the last day, samples were washed thrice in 5×SSCT for 15 min at RT. Nuclear staining was performed by incubating samples in 5×SSCT containing DAPI (1:1000) for 30 min at RT, followed by three 10 min washes in 5×SSCT. Samples were stored in 5×SSCT at 4 °C in the dark until mounting for imaging.

##### Mounting

Stained samples were mounted between two coverslips with VECTASHIELD® Antifade Mounting Medium (Vector Laboratories).

##### Gelatin-embedding stained embryos

IF- and HCR-stained embryos were embedded in gelatin for cryosectioning. Stained embryos were protected from light at all steps. First, stained embryos were washed with PBS−/−. Stained embryos were then cryoprotected by incubation in 5% sucrose (Thermo Fisher Scientific) in PBS−/− at RT for 10–30 min, followed by overnight incubation in 15% sucrose in PBS−/− at 4 °C until fully sunk. The next day, embryos were equilibrated in molten gelatin (7.5% gelatin + 15% sucrose + PBS−/−) at 37 °C for 3–5 h, then embedded in cryomolds (Agar Scientific). Embryos were positioned near the bottom of the mould using forceps, and blocks were solidified at 4 °C for at least 5 min. Once set, moulds were flash-frozen by brief sequential submersion in liquid nitrogen and stored at −80 °C until sectioning.

##### Cryosectioning

Gelatin-embedded embryos were cryosectioned using a Microtome Leica Cryostat 3050S. Samples were equilibrated inside the cryostat chamber (chamber temperature −20 °C; object temperature −14 °C) for at least 10 min prior to sectioning. Sample blocks were mounted onto the specimen stage using EprediaTM Neg-50TM Frozen Section Medium (Thermo Fisher Scientific) or OCT compound (Agar Scientific), and trimmed at 50 μm before sectioning at 25 μm. Sections were collected onto EprediaTM SuperFrost PlusTM Adhesion slides (Thermo Fisher Scientific) and air-dried overnight at RT in the dark. Slides were stored at 4 °C, protected from light, until imaging.

##### SNAI2 overexpression

The coding sequence of *SNAI2*, spanning from the ATG start codon to the stop codon, was amplified from cDNA by PCR with flanking XhoI restriction sites. The amplified fragment was first cloned into the pSC-A plasmid (Strataclone) for sequence verification. Subsequently, the verified insert was subcloned into the pCIG expression vector using XhoI, positioned upstream of the IRES2.

##### SNAI2 CRISPR-Cas9

CRISPR/Cas9-mediated gene editing was performed as previously described (Williams et al, 2018). The Cas9-expressing plasmid pCAG Cas9-2A-Citrine (Addgene #92358) and the chick-specific sgRNA vector pcU6-3 sgRNA (Addgene #92359) were used for electroporation. The *SNAI2*-sgRNA target sequence (5’ ACCTCCTCCAAAGATCACAG 3’) was selected using CHOPCHOP (Labun et al, 2019) based on low off-target potential and the presence of a diagnostic restriction enzyme site for genotyping. The sgRNA was cloned into the pcU6-3 vector using annealed oligonucleotides and Golden Gate assembly with BsmBI, followed by transformation, colony PCR, and sequencing to confirm correct insertion. Genomic DNA was extracted from dissected tissues using either crude extraction of alkaline lysis (50 mM NaOH, 95 °C) followed by neutralisation (1 M Tris-HCl, pH 8.0) or PureLinkTM Genomic DNA Mini Kit (InvitrogenTM). Genotyping was performed by PCR amplification of the target region, restriction digest, and agarose gel electrophoresis to distinguish wild-type from successfully CRISPR-edited mosaic tissues.

#### Data analysis

##### Bulk RNA sequencing data analysis

Raw reads were processed using the Galaxy web platform (Afgan et al, 2016). Quality control was performed using FastQC (Simon Andrews et al, 2010), and adaptor trimming was performed with Trim Galore! (Krueger et al, 2023), alignment to the galGal5 reference genome from the Genome Reference Consortium was carried out using HISAT 2 (Kim et al, 2015), PCR duplicates were removed using SAMtools markdup (Li et al, 2009), transcript assembly was performed with StringTie (Pertea et al, 2015), and differential expression analysis was carried out using DESeq2 (Love et al, 2014).

##### Gene ontology

Gene ontology enrichment analyses were conducted using GeneCodis 4 (Garcia-Moreno et al, 2022).

##### Single-cell RNA sequencing data analysis

CellRanger (10X Genomics) filtered feature matrices were passed into Seurat (Satija et al, 2015) in R v4.2.3. Reads were mapped to the galGal6 reference genome from the Genome Reference Consortium. The following quality control steps were taken: checking the % of mitochondrial genes, filtering out cells with too low or high number of different genes expressed, normalising the data, and regressing for both sex effect and cell cycle. Principal Component Analysis (PCA) was then performed for dimensionality reduction, followed by Uniform Manifold Approximation and Projection (UMAP) using the first 12 principal components (dims = 1:12) and a clustering resolution of 0.30 for visualisation of cellular transcriptomic profiles. Feature plots were generated to display the expression patterns of genes of interest across the UMAP space. Correlation analyses were conducted between the expression levels of genes of interest across individual cells to assess co-expression dynamics.

##### Imaging & image analysis

Fixed and HCR- or immuno-stained samples were imaged on the Zeiss LSM700 confocal microscope. Images were analysed using FIJI, to create either maximum projection or resliced images. Snapshots of live embryos on EC culture plates were imaged on a fluorescent dissecting scope (Leica) in brightfield and GFP channels. Images were overlaid using FIJI. Live imaging of embryos on EC culture plates and explants were performed using a Nikon Eclipse Ti widefield microscope. Further quantification of HCR- or immuno-stained samples were performed using IMARIS.

##### Statistical analysis

Statistical tests were performed according to the structure of each dataset and are detailed below. Exact test statistics, sample sizes, and *p* values for each comparison are reported in the corresponding figure legends. Significance is denoted as n.s. (not significant), *p < 0.05, **p < 0.05, ***p < 0.005, ****p < 0.0005. All statistical analyses were performed in R, using the base stats package (t.test, wilcox.test) and the car package (leveneTest).

## Supplementary information

Appendix

Peer Review File

Movie EV1

Movie EV2

Datasets

Source data Fig. 1

Source data Fig. 2

Source data Fig. 3

Source data Fig. 4

Source data Fig. 5

Source data Fig. 6

Expanded View Figures

## Data Availability

The Bulk RNA sequencing data generated in this publication have been deposited in NCBI’s Gene Expression Omnibus (Edgar et al, 2002) and are publicly available as of the date of publication through GEO Series accession number GSE312350. https://www.ncbi.nlm.nih.gov/geo/query/acc.cgi?acc=GSE312350. The single-cell RNA sequencing data generated in this publication have been deposited in NCBI’s Gene Expression Omnibus (Edgar et al, 2002) and are publicly available as of the date of publication through GEO Series accession number GSE314634. https://www.ncbi.nlm.nih.gov/geo/query/acc.cgi?acc=GSE314634. The source data of this paper are collected in the following database record: biostudies:S-SCDT-10_1038-S44318-026-00916-8.
