# Supplementary material for: Reciprocal coupling between EMT and BMP morphogen signalling drives collective cell invasion
Source: EMBO J. 2026 Oct 7;45(21):7347–73. doi: 10.1038/s44318-026-00916-8 (PMC13728173; doi:10.1038/s44318-026-00916-8)
Supplement: Supplementary file 1 [file 44318_2026_916_MOESM12_ESM.pdf]

Expanded View Figures

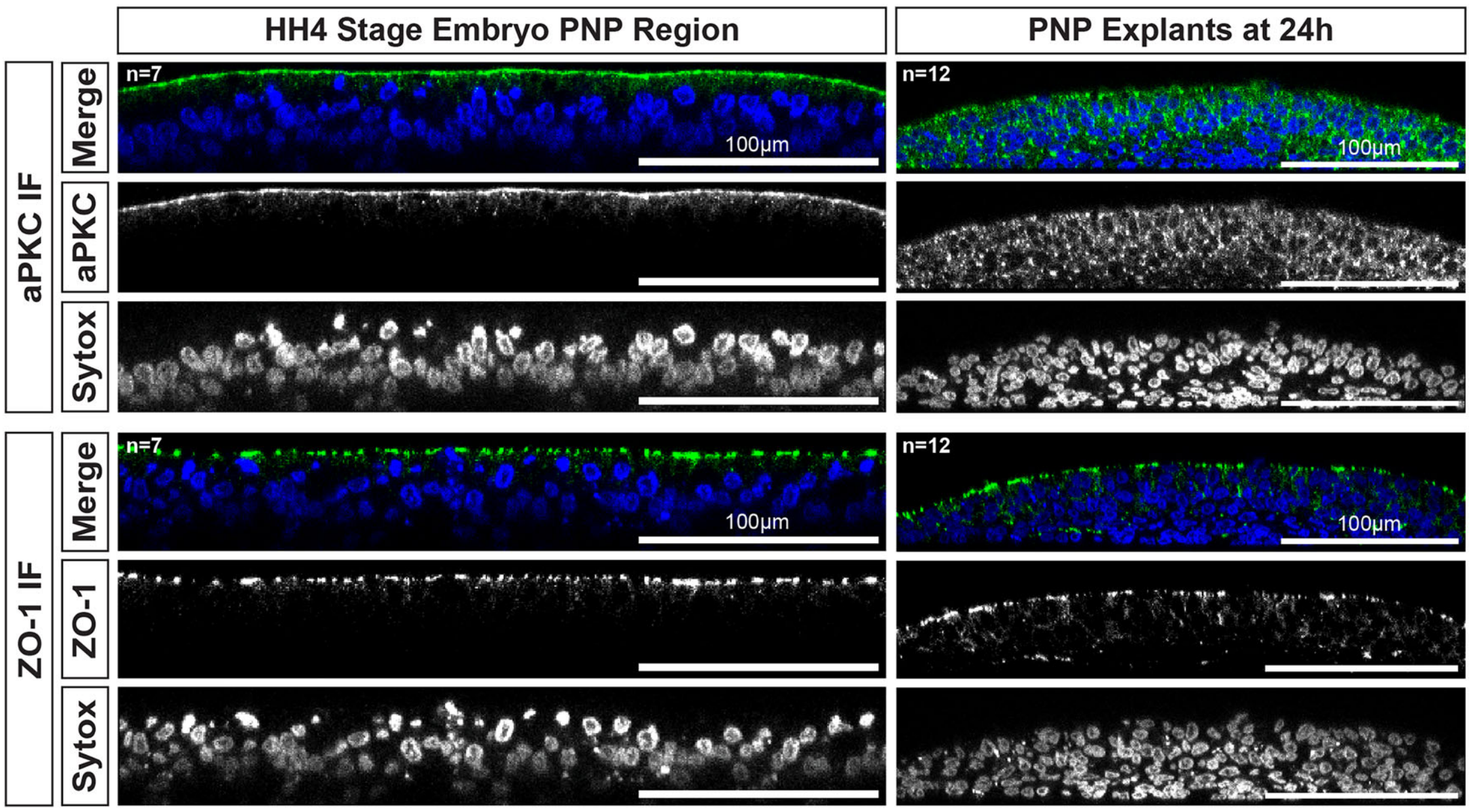

Figure EV1. Loss of apical-basal polarity observed in PNP migrating explants.

Reslices of representative confocal image of aPKC and ZO-1 immunofluorescence and SYTOX staining on the PNP embryonic region and PNP migrating explants on human fibronectin after 24 h of incubation.

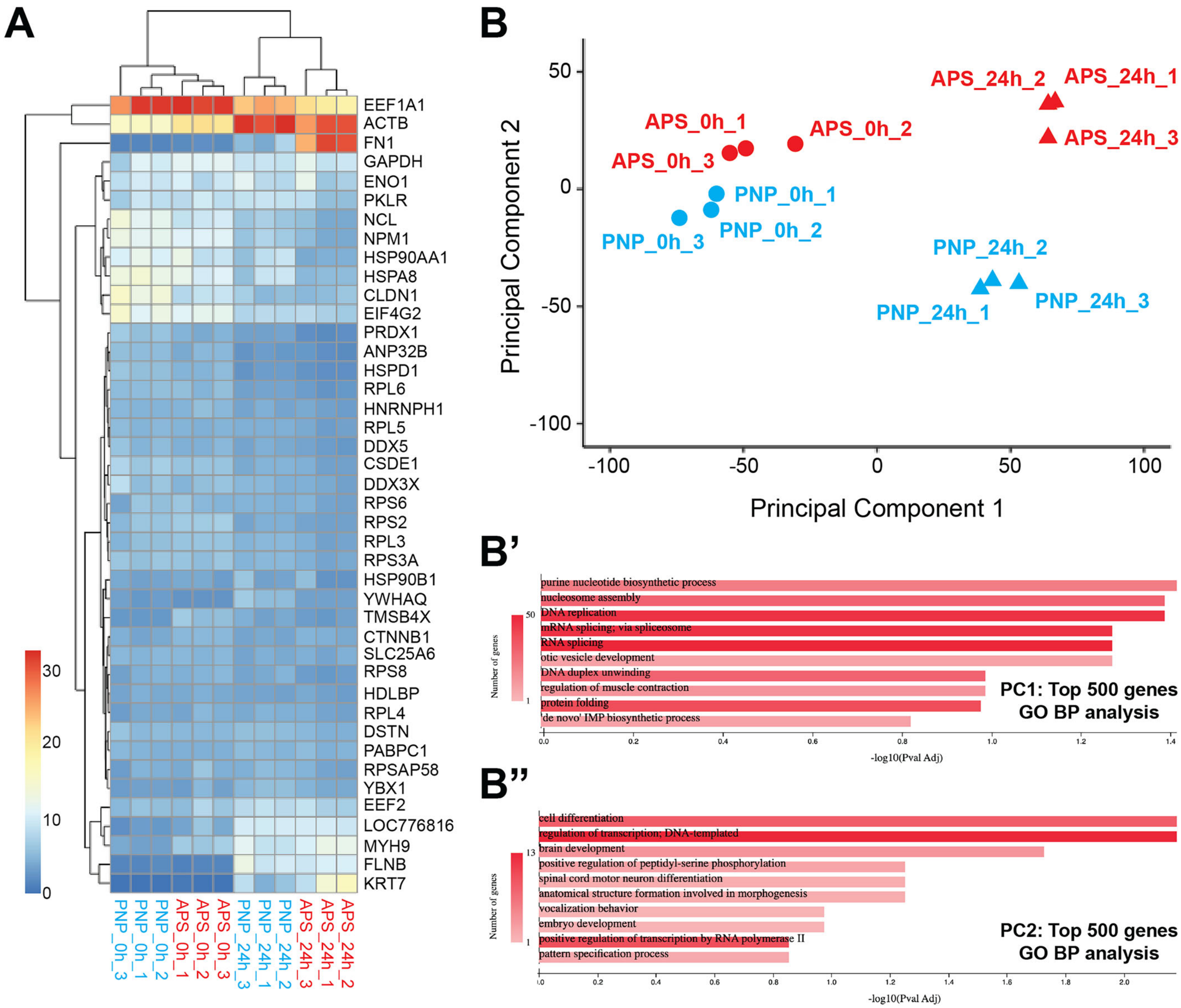

Figure EV2. Bulk RNA sequencing reveals common genes upregulated or downregulated in migrating explants, regardless of tissue type.

(**A**) Z-score heatmap showing the top differentially expressed genes across all samples, filtered by a summed z-score threshold (>50). Genes are hierarchically clustered, and samples are grouped by tissue type (PNP vs APS) and timepoint (24 vs 0 h). (**B**) Principal component analysis (PCA) of all bulk RNA sequencing samples reveals clear separation of samples at 24 vs 0 h along PC1 and PC2. PC1 accounts for variation primarily associated with DNA replication, transcription, and translation. PC2 corresponds to changes in cell differentiation status. (**B'**) Gene Ontology (GO) Biological Process (BP) enrichment of the top 500 genes contributing most to PC1, highlighting pathways associated with biosynthesis, translation, and cell cycle progression. (**B''**) GO BP enrichment of the top 500 genes contributing most to PC2, showing enrichment for terms related to differentiation and developmental processes.

 © The Author(s)

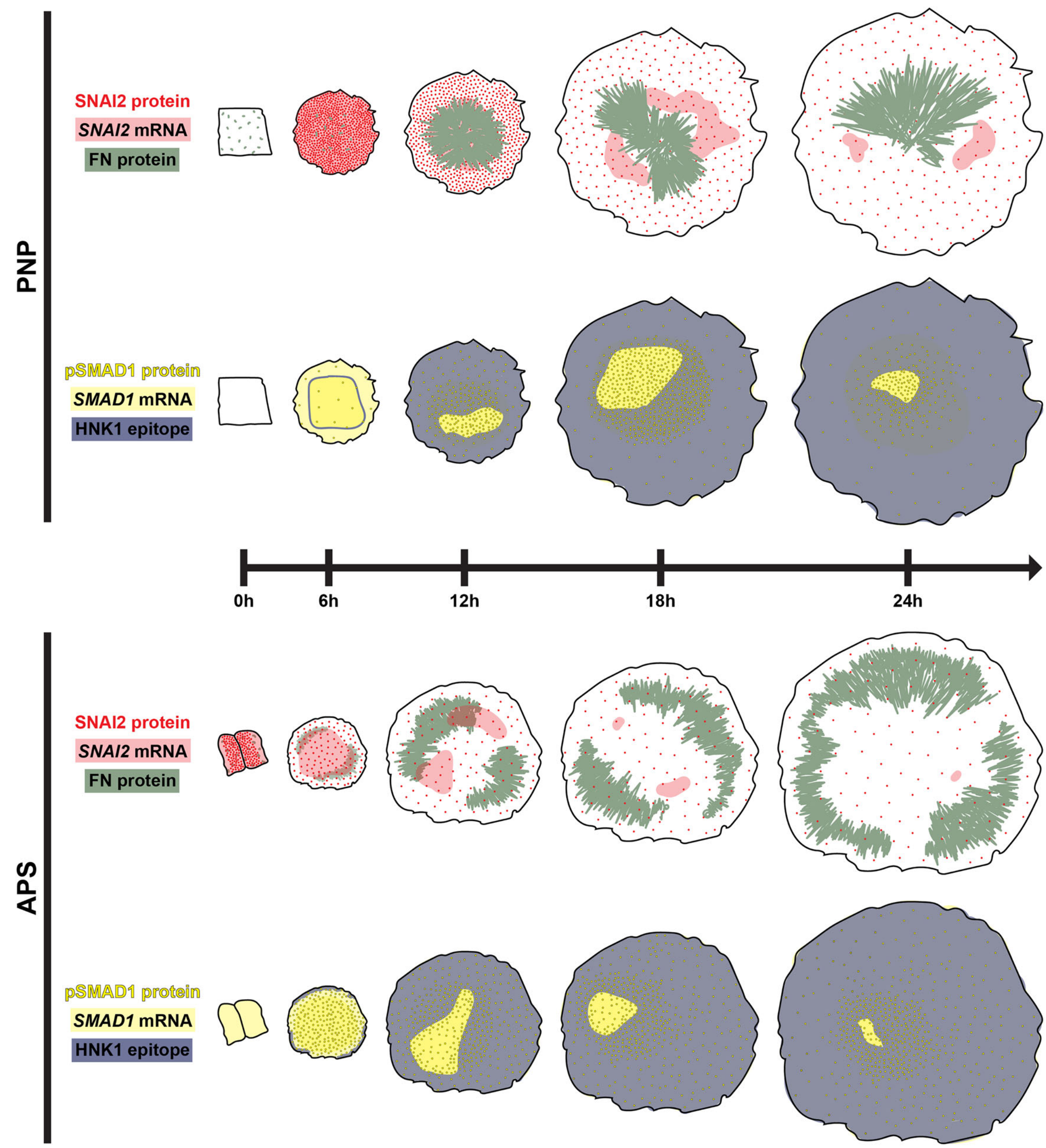

Figure EV3. Summary of pseudo-time-course analysis of markers.

Schematic of observed expression of SNAI2 protein, *SNAI2* mRNA, FN protein, pSMAD1 protein, *SMAD1* mRNA, and HNK1 epitope in PNP and APS migrating explants at multiple timepoints.

© The Author(s)

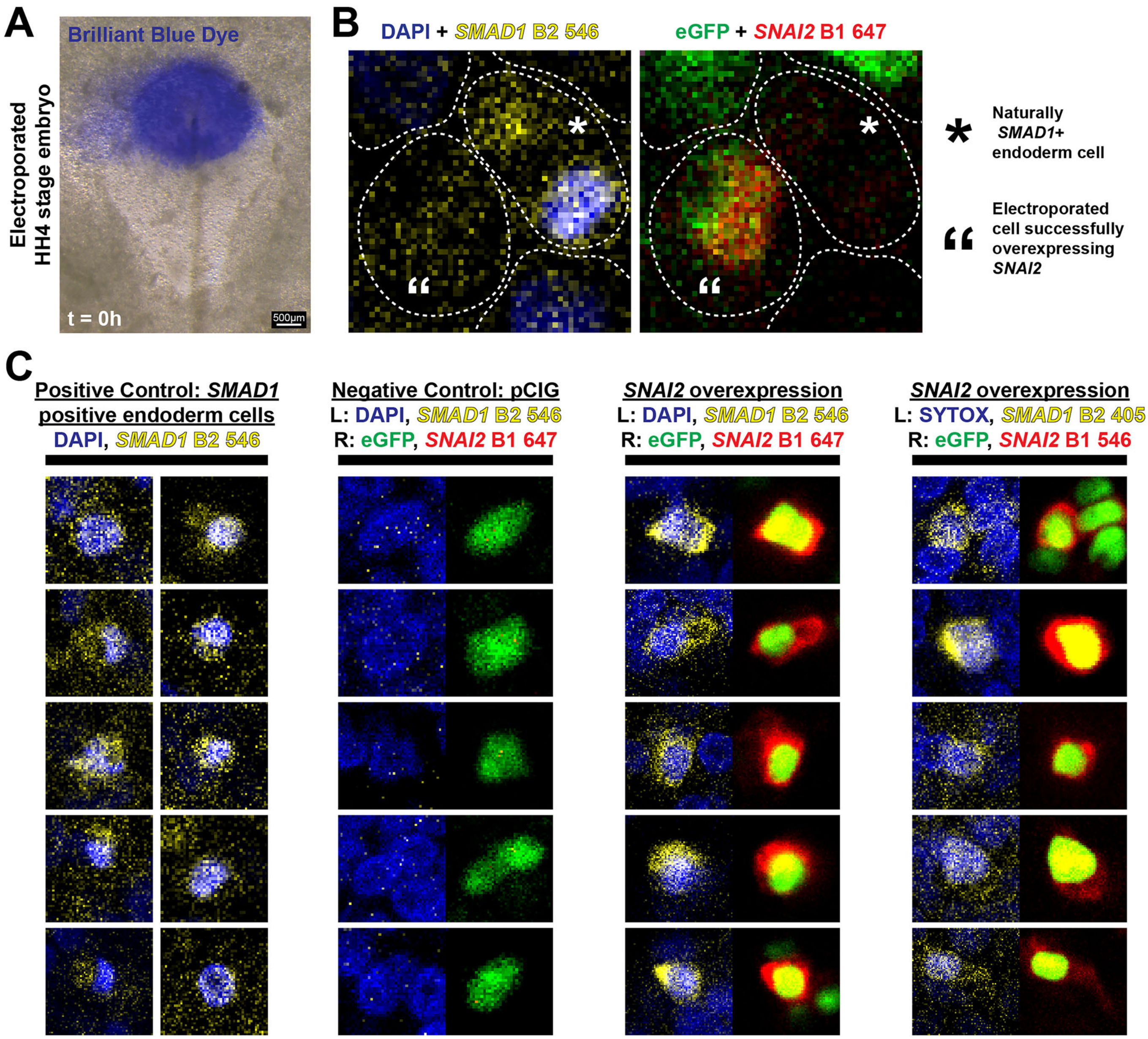

Figure EV4. *SNAI2* overexpression is sufficient to drive *SMAD1* expression.

(**A**) Representative brightfield image of an electroporated embryo at $t = 0$ h. (**B**) A representative confocal image showing a naturally *SMAD1*-positive endoderm cell next to an electroporated cell. (**C**) Representative confocal images of *SNAI2* and *SMAD1* in situ HCR staining of endoderm cells (left; positive control for *SMAD1* expression), pCIG-electroporated cells (middle; negative control for *SNAI2* expression), and cells electroporated with the *SNAI2*-overexpression construct (right two columns; experimental; different HCR hairpin combinations). Images were cropped from the original images for visualisation purposes.

 © The Author(s)

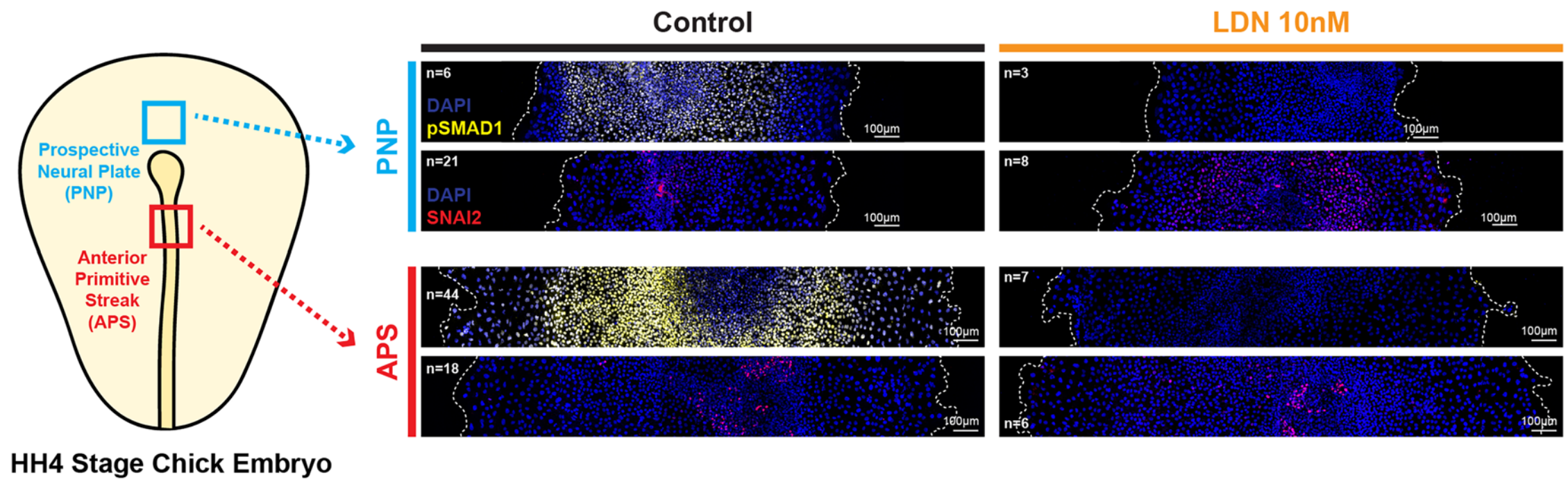

Figure EV5. BMP signalling not needed for EMT initiation in ex vivo explants.

Representative confocal images of pSMAD1 and SNAI2 immunofluorescence and SYTOX staining in control and LDN-treated PNP and APS explants on human fibronectin after 24 h of incubation.

© The Author(s)
