# Supplementary material for: Reciprocal coupling between EMT and BMP morphogen signalling drives collective cell invasion
Source: EMBO J. 2026 Oct 7;45(21):7347–73. doi: 10.1038/s44318-026-00916-8 (PMC13728173; doi:10.1038/s44318-026-00916-8)
Supplement: Supplementary file 2 [file 44318_2026_916_MOESM1_ESM.pdf]

Appendix

Table of Contents

Appendix Figures... 2 - 23

Appendix Figures

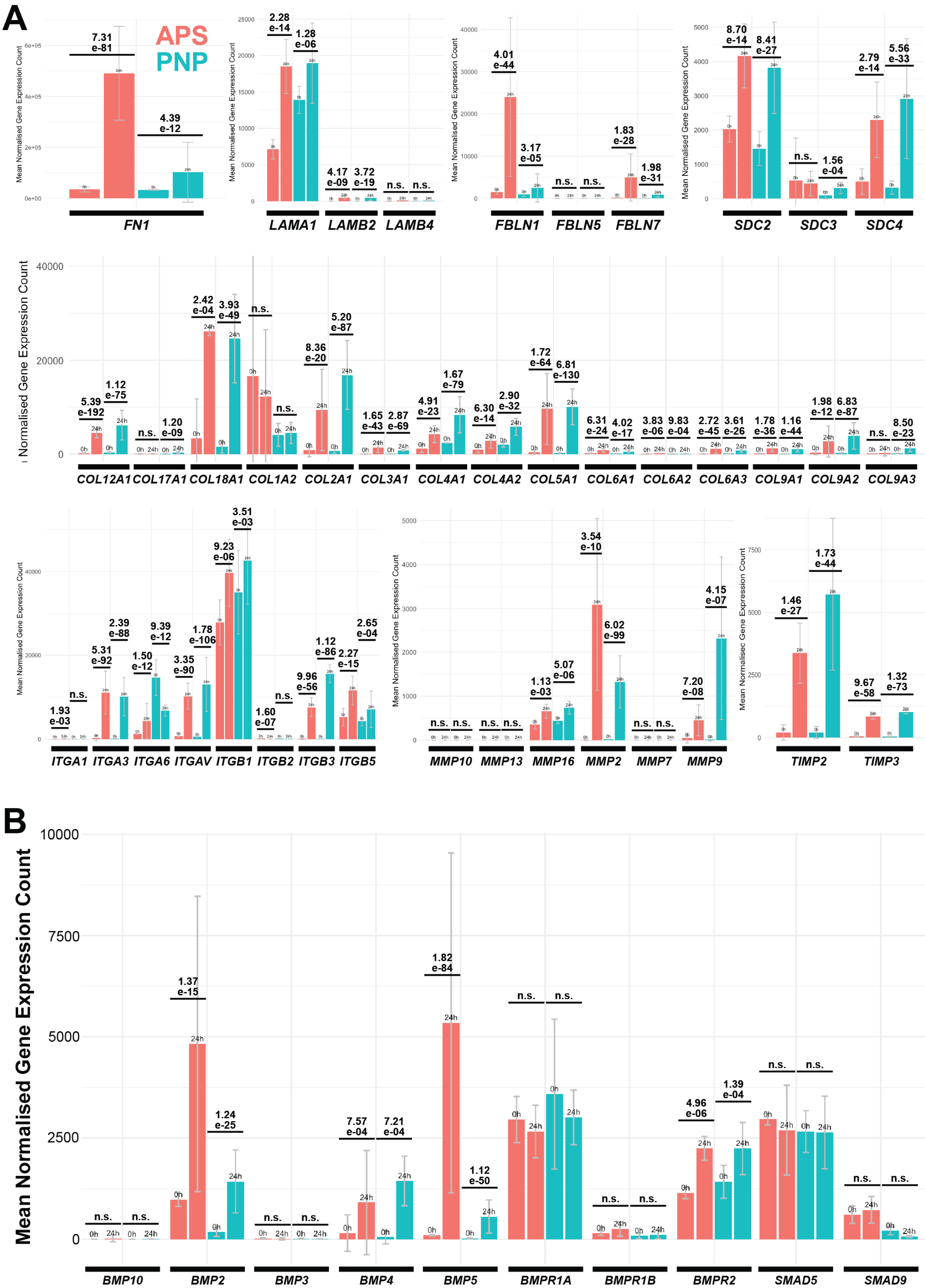

Appendix Figure S1. Bulk RNA sequencing reveals upregulation of many ECM components, ECM remodelling enzymes, and BMP signalling pathway components. A) Bulk RNA sequencing mean normalised gene expression counts for ECM component and ECM remodelling genes of interest in PNP and APS migrating explants at 0h vs 24h of incubation. FN: Fibronectin. LAM: Laminin. FBLN: Fibulin. SDC: Syndecan. COL: Collagen. ITG: Integrin. MMP: Matrix metalloproteases. TIMP: Tissue inhibitor of metalloproteinases. B) Bulk RNA sequencing mean normalised gene expression counts for genes encoding BMP ligands and receptors in PNP and APS migrating explants at 0h vs 24h of incubation. DESeq2 p-values.

Biological Pathway

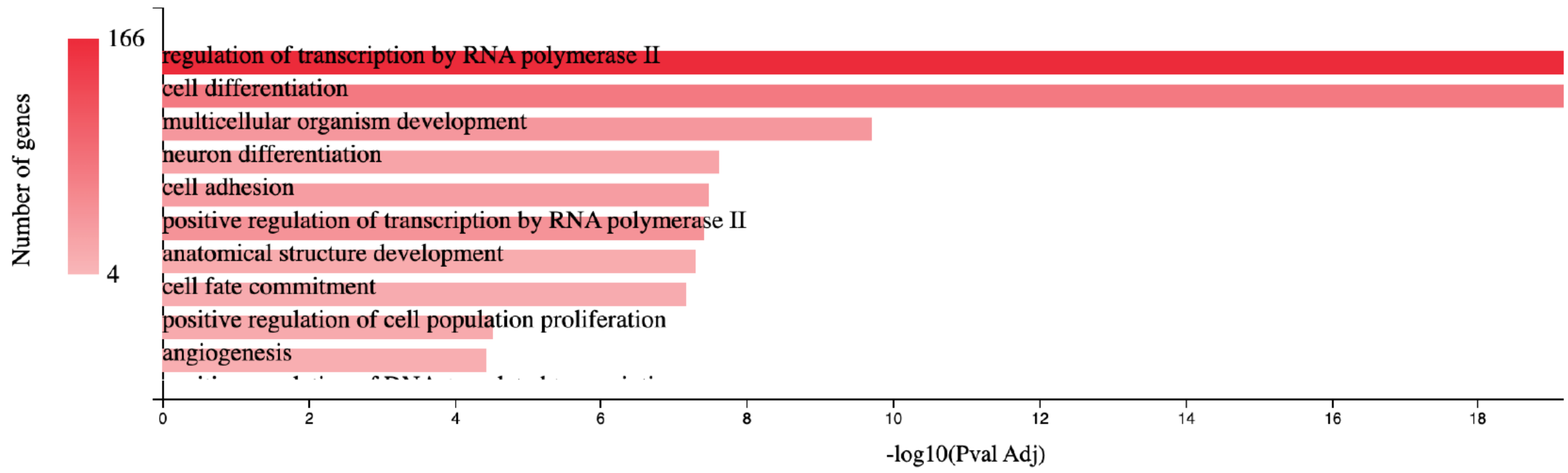

Cellular Component

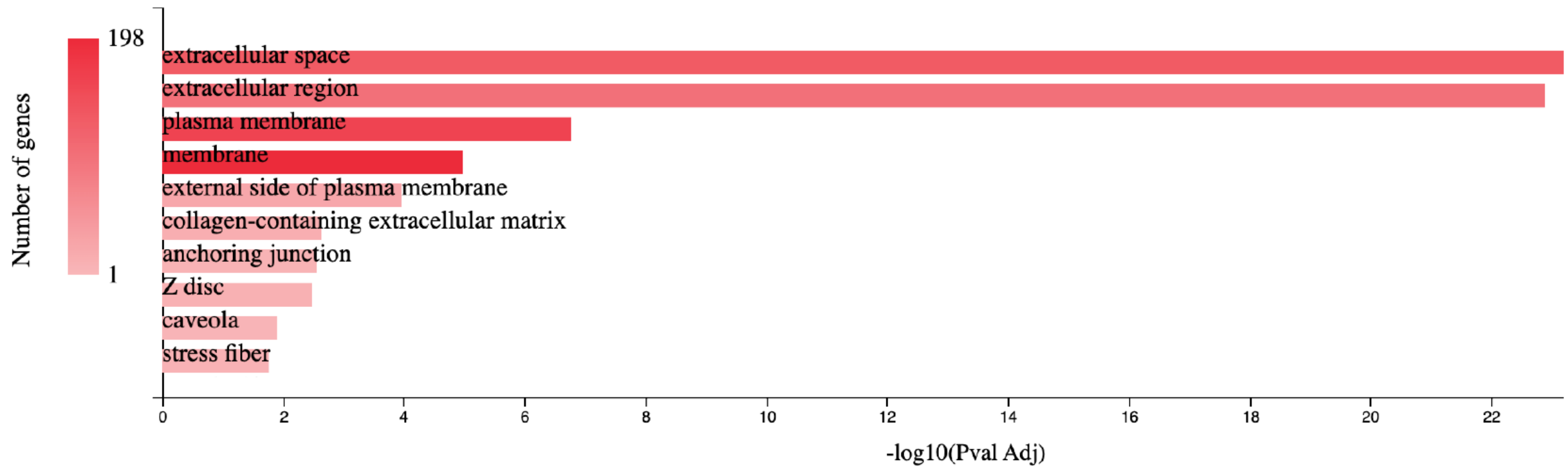

Molecular Function

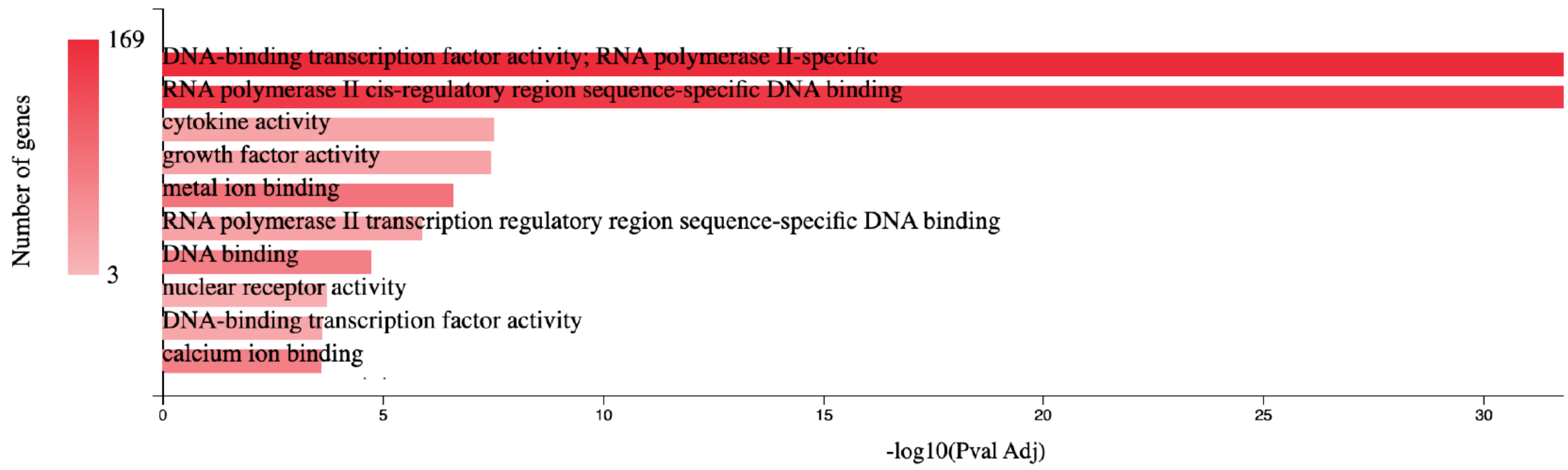

KEGG Pathway

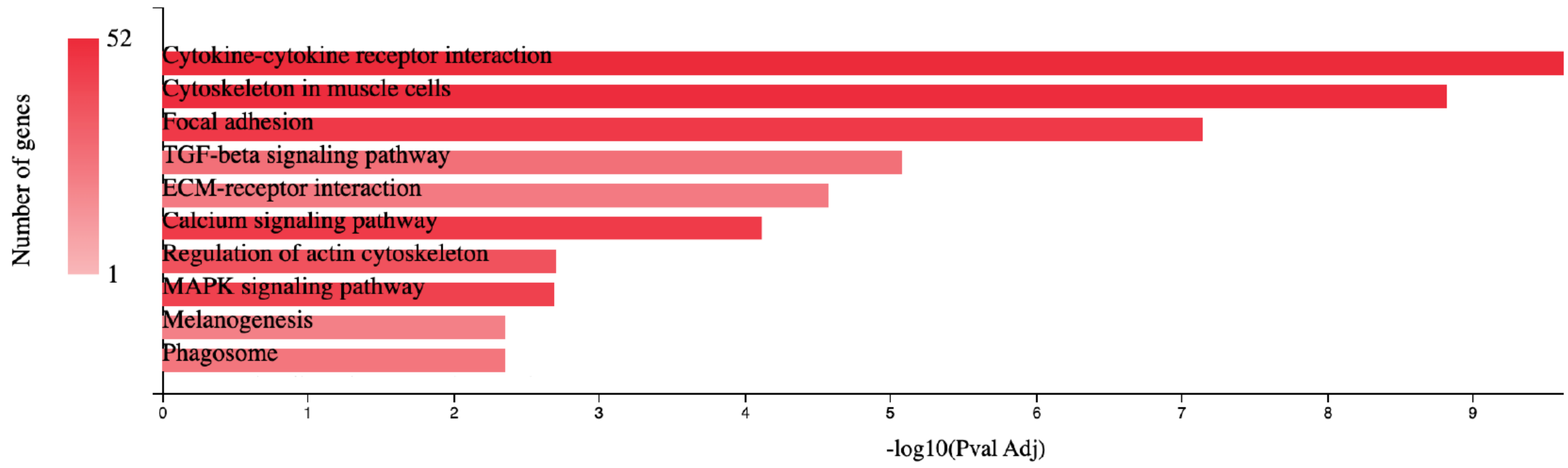

Appendix Figure S2. Gene ontology of genes upregulated at least 2-fold in both PNP and APS tissue upon migration. Top 10 terms shown.

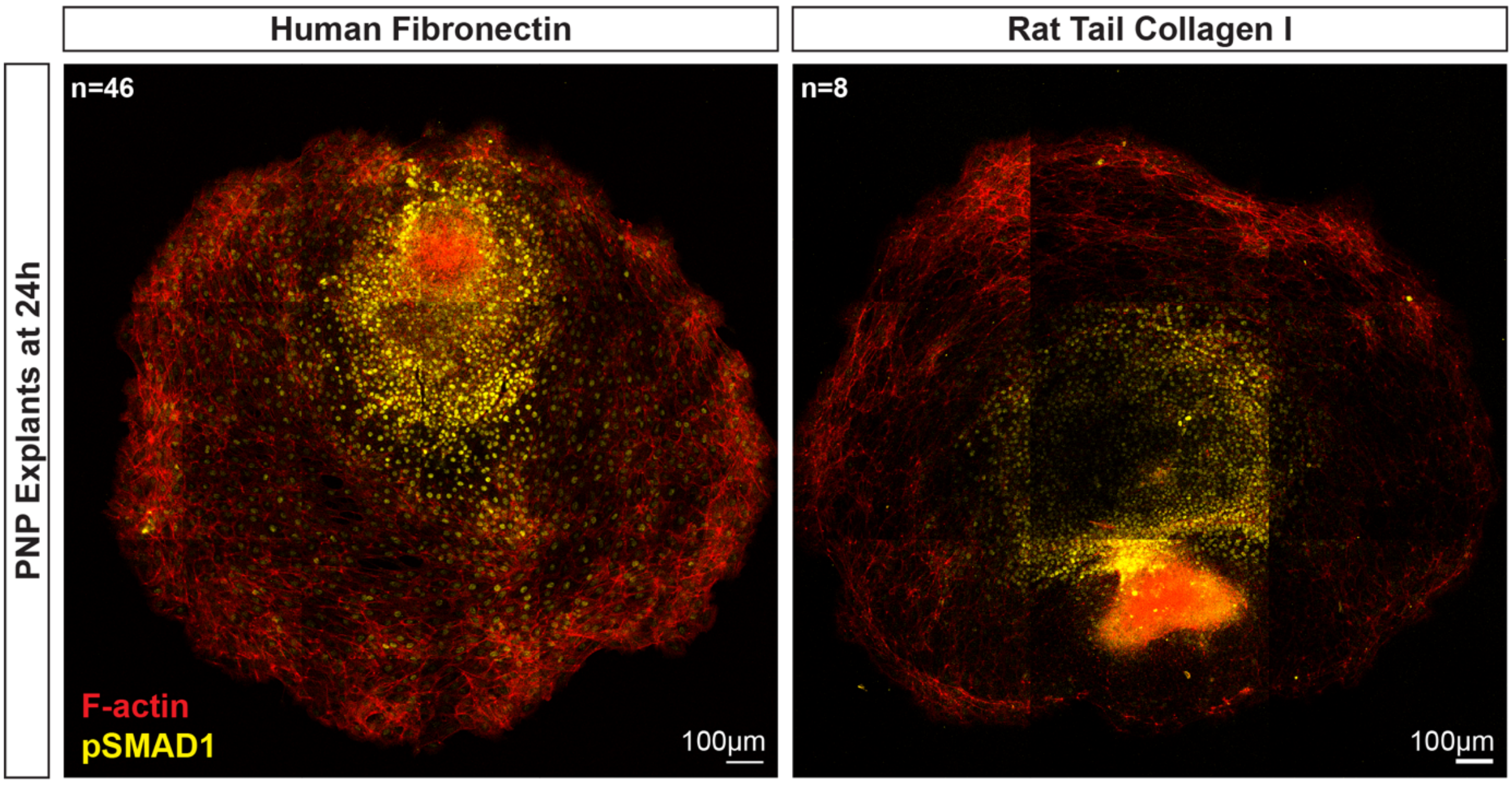

Appendix Figure S3. BMP signalling activity induced in PNP *ex vivo* migrating explants on fibronectin and collagen. Representative confocal image of phospho-SMAD1 immunofluorescence and F-actin staining on an PNP migrating explant on human fibronectin vs. rat tail collagen I after 24h of incubation.

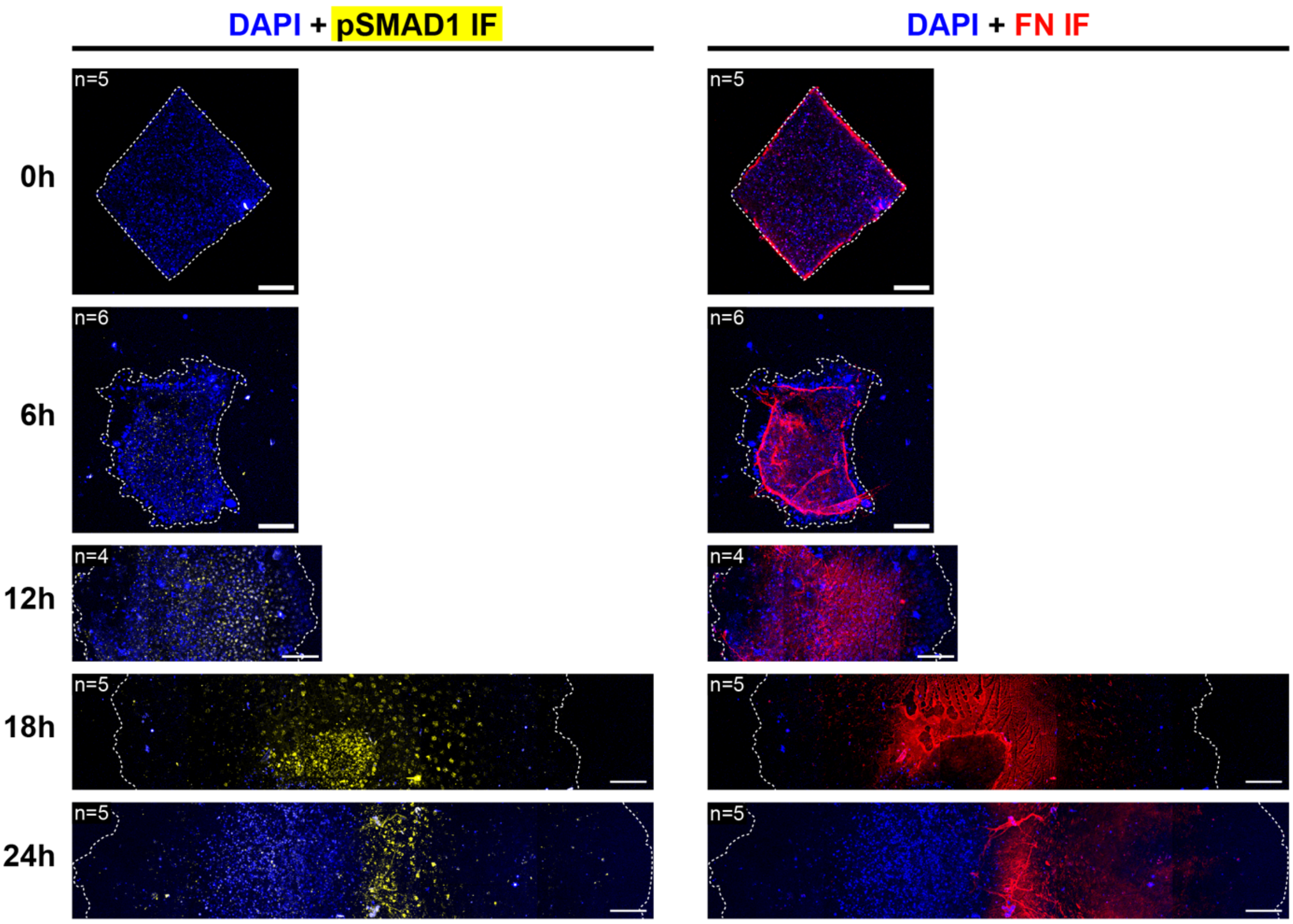

Appendix Figure S4. Pseudo time-course analysis of FN and pSMAD1 expression in PNP migrating explants. Representative confocal images of FN and pSMAD1 immunofluorescence and DAPI staining in PNP migrating explants on human fibronectin at 0, 6, 12, 18, and 24 hours of culture.

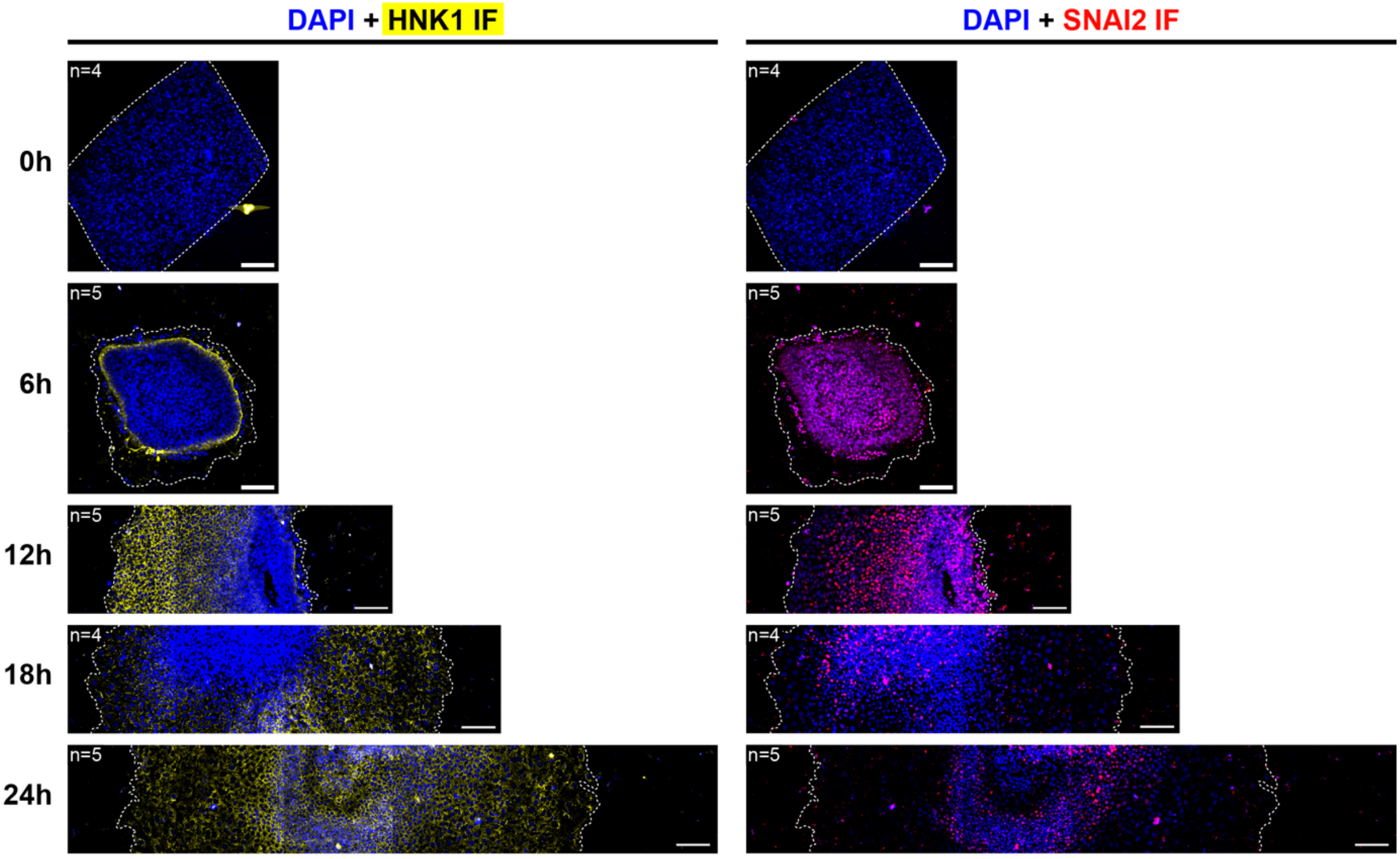

Appendix Figure S5. Pseudo time-course analysis of HNK1 and SNAI2 expression in PNP migrating explants. Representative confocal images of HNK1 and SNAI2 immunofluorescence and DAPI staining in PNP migrating explants on human fibronectin at 0, 6, 12, 18, and 24 hours of culture.

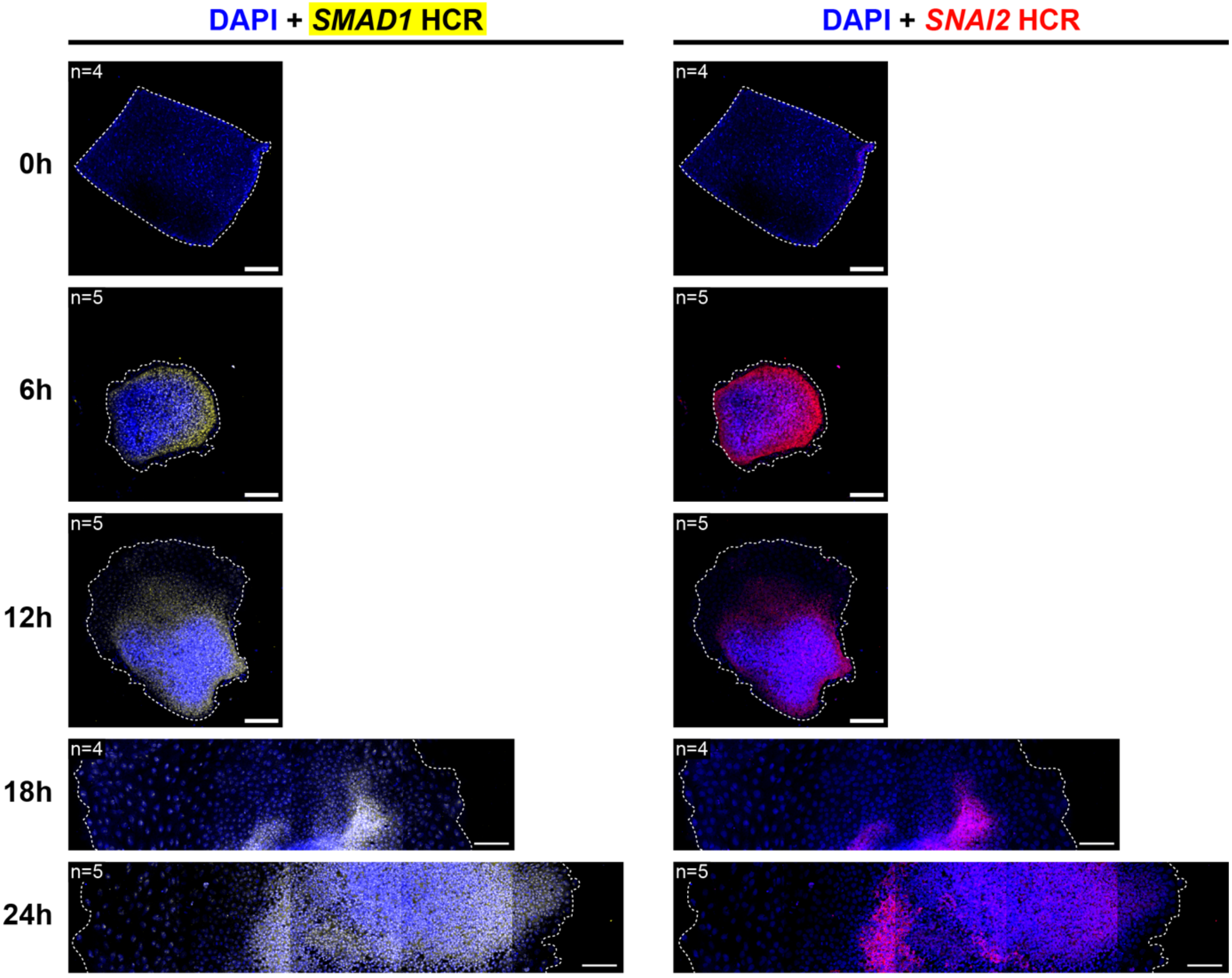

Appendix Figure S6. Pseudo time-course analysis of *SNAI2* and *SMAD1* expression in PNP migrating explants. Representative confocal images of *SNAI2* and *SMAD1* HCR and DAPI staining in PNP migrating explants on human fibronectin at 0, 6, 12, 18, and 24 hours of culture.

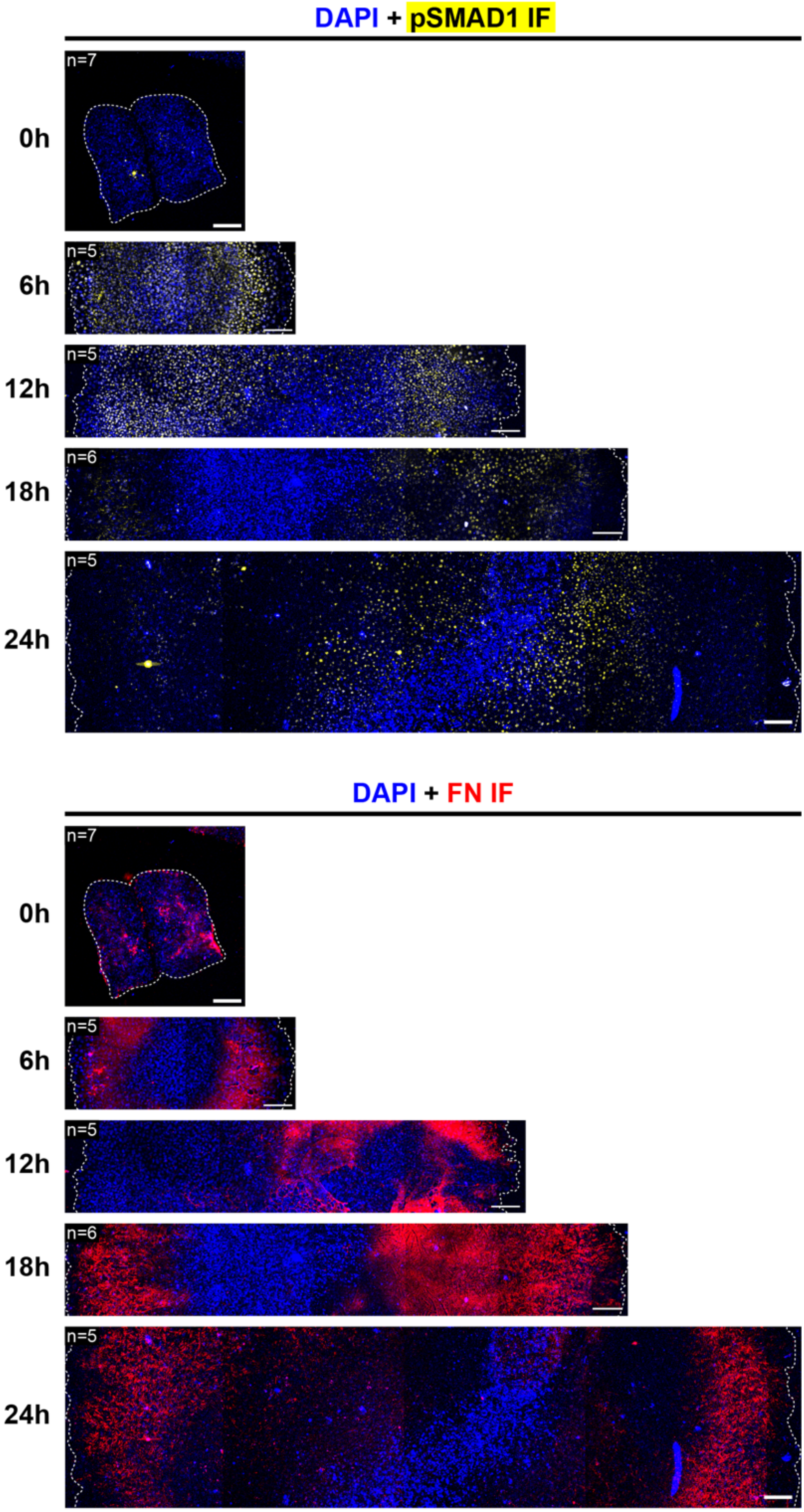
DAPI + pSMAD1 IF
0h
n=7
6h
n=5
12h
n=5
18h
n=6
24h
n=5
DAPI + FN IF
0h
n=7
6h
n=5
12h
n=5
18h
n=6
24h
n=5

Appendix Figure S7. Pseudo time-course analysis of FN and pSMAD1 expression in APS migrating explants. Representative confocal images of FN and pSMAD1 immunofluorescence and DAPI staining in APS migrating explants on human fibronectin at 0, 6, 12, 18, and 24 hours of culture.

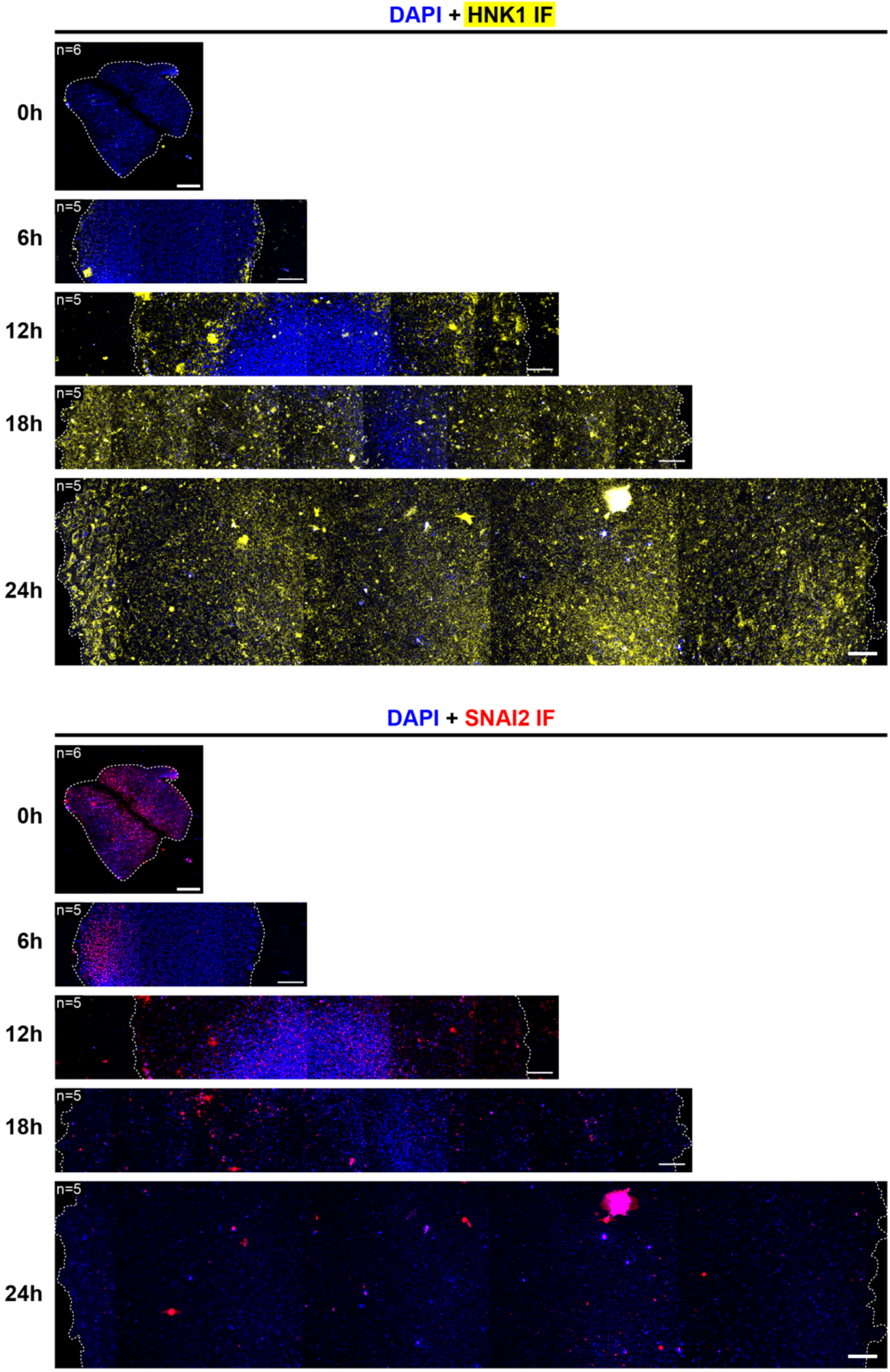
DAPI + HNK1 IF
0h
n=6
6h
n=5
12h
n=5
18h
n=5
24h
n=5
DAPI + SNAI2 IF
0h
n=6
6h
n=5
12h
n=5
18h
n=5
24h
n=5

Appendix Figure S8. Pseudo time-course analysis of HNK1 and SNAI2 expression in APS migrating explants. Representative confocal images of HNK1 and SNAI2 immunofluorescence and DAPI staining in APS migrating explants on human fibronectin at 0, 6, 12, 18, and 24 hours of culture.

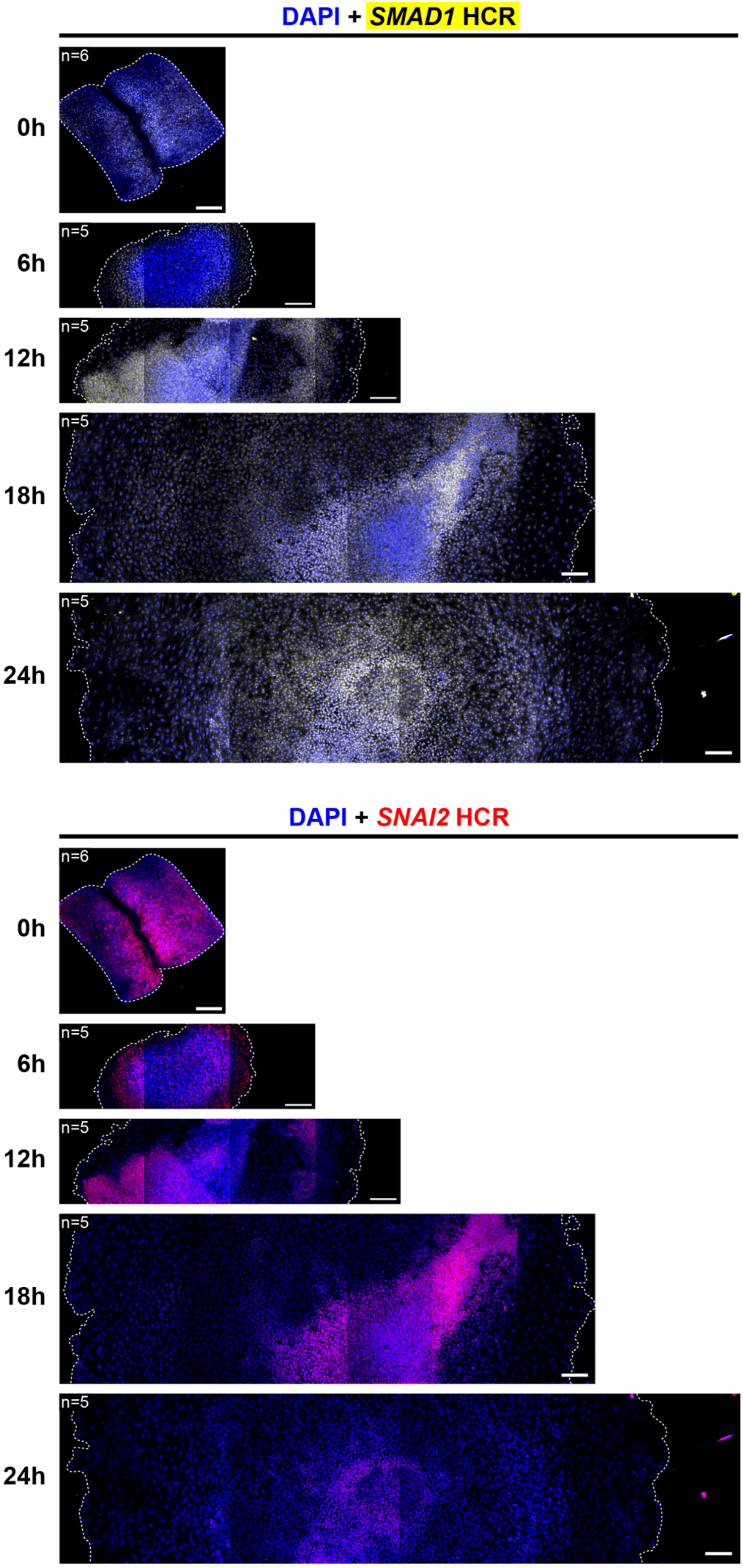
DAPI + SMAD1 HCR
0h
n=6
6h
n=5
12h
n=5
18h
n=5
24h
n=5
DAPI + SNAI2 HCR
0h
n=6
6h
n=5
12h
n=5
18h
n=5
24h
n=5

Appendix Figure S9. Pseudo time-course analysis of *SNAI2* and *SMAD1* expression in APS migrating explants. Representative confocal images of *SNAI2* and *SMAD1* HCR and DAPI staining in APS migrating explants on human fibronectin at 0, 6, 12, 18, and 24 hours of culture.

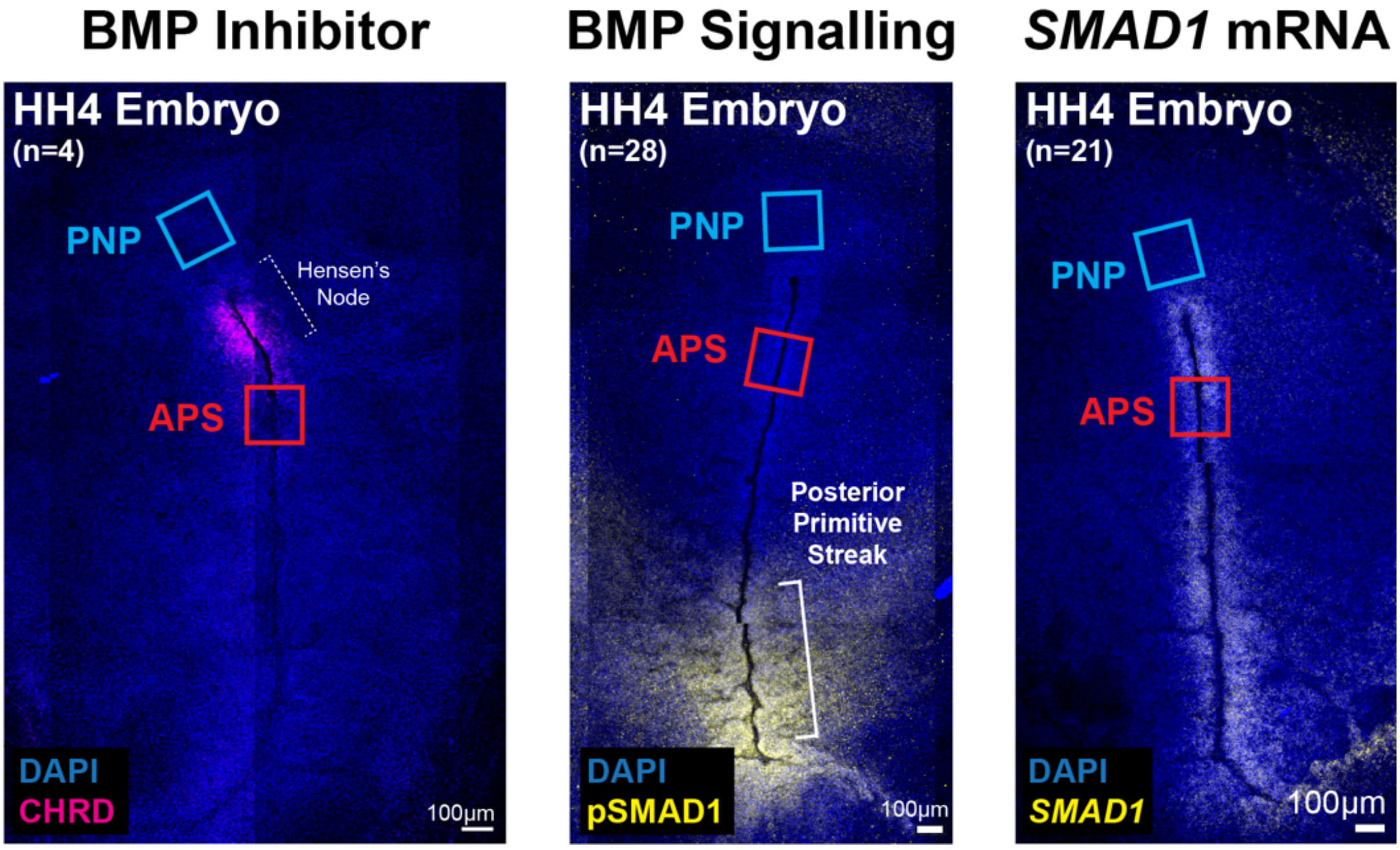

Appendix Figure S10. BMP signalling activation in PNP migrating explants is not due to lifting of BMP inhibition by Hensen's node. Representative confocal images of HH4 stage chick embryo *CHRD in situ* HCR staining (left), phospho-SMAD1 immunostaining (middle; re-display of image from Figure 1F), and *SMAD1 in situ* HCR staining (right; re-display of image from Figure 2E).

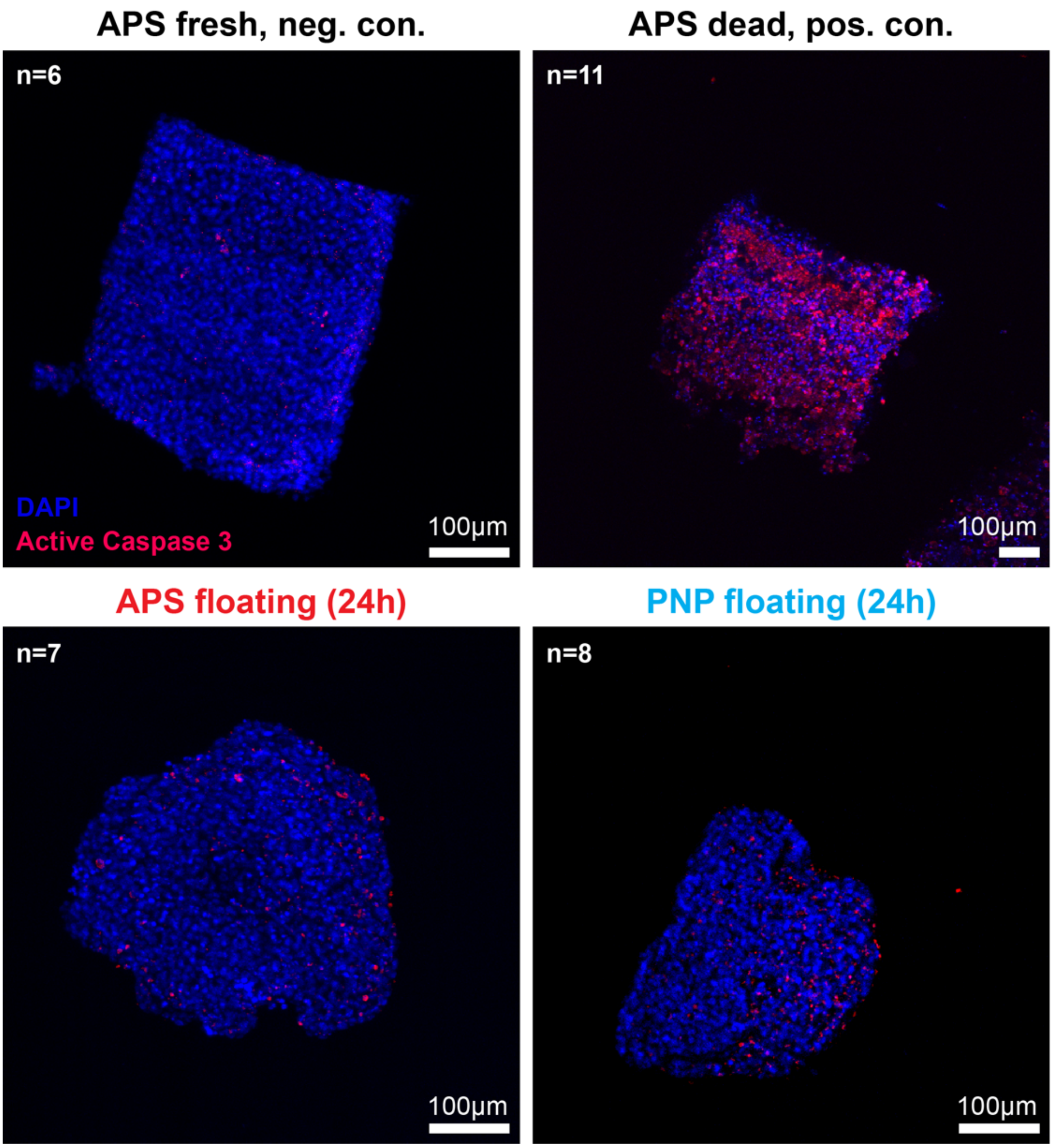

Appendix Figure S11. Floating PNP and APS explants have minimal Caspase 3 activity after 24 hours of incubation. Representative confocal images of PNP and APS floating explant active Caspase 3 immunostaining. Negative control: freshly dissected APS tissue. Positive control: APS explant incubated 24 hours in 100 µM LDN.

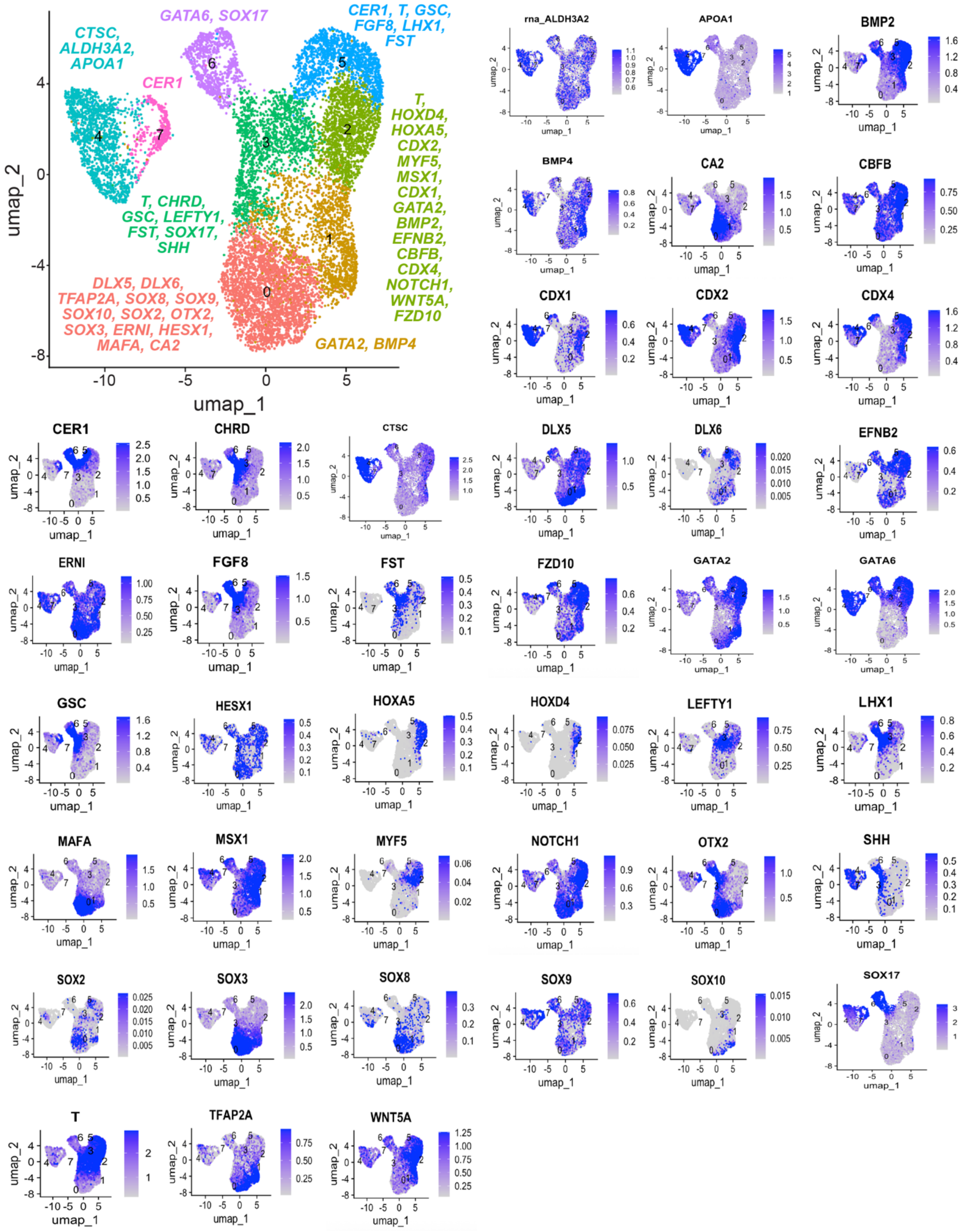

Appendix Figure S12. UMAP cluster annotations. A List of relevant top differentially expressed genes per cluster, and their feature maps in alphabetical order. Colour-coded UMAP re-displayed from Figure 2C.

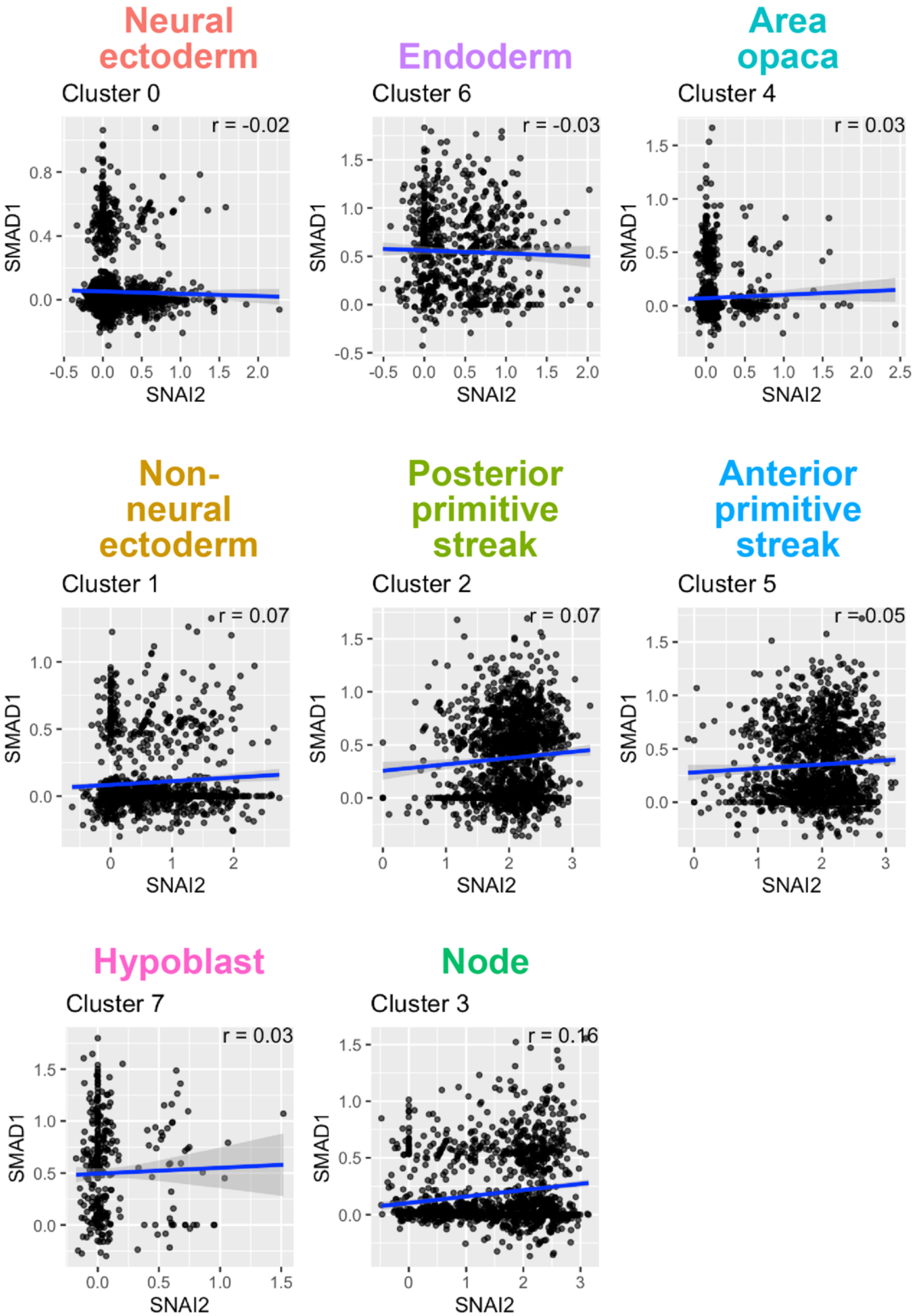

Appendix Figure S13. Correlation between *SNAI2* and *SMAD1* gene expression in individual clusters. Pearson's correlation between *SMAD1* and *SNAI2* expression.

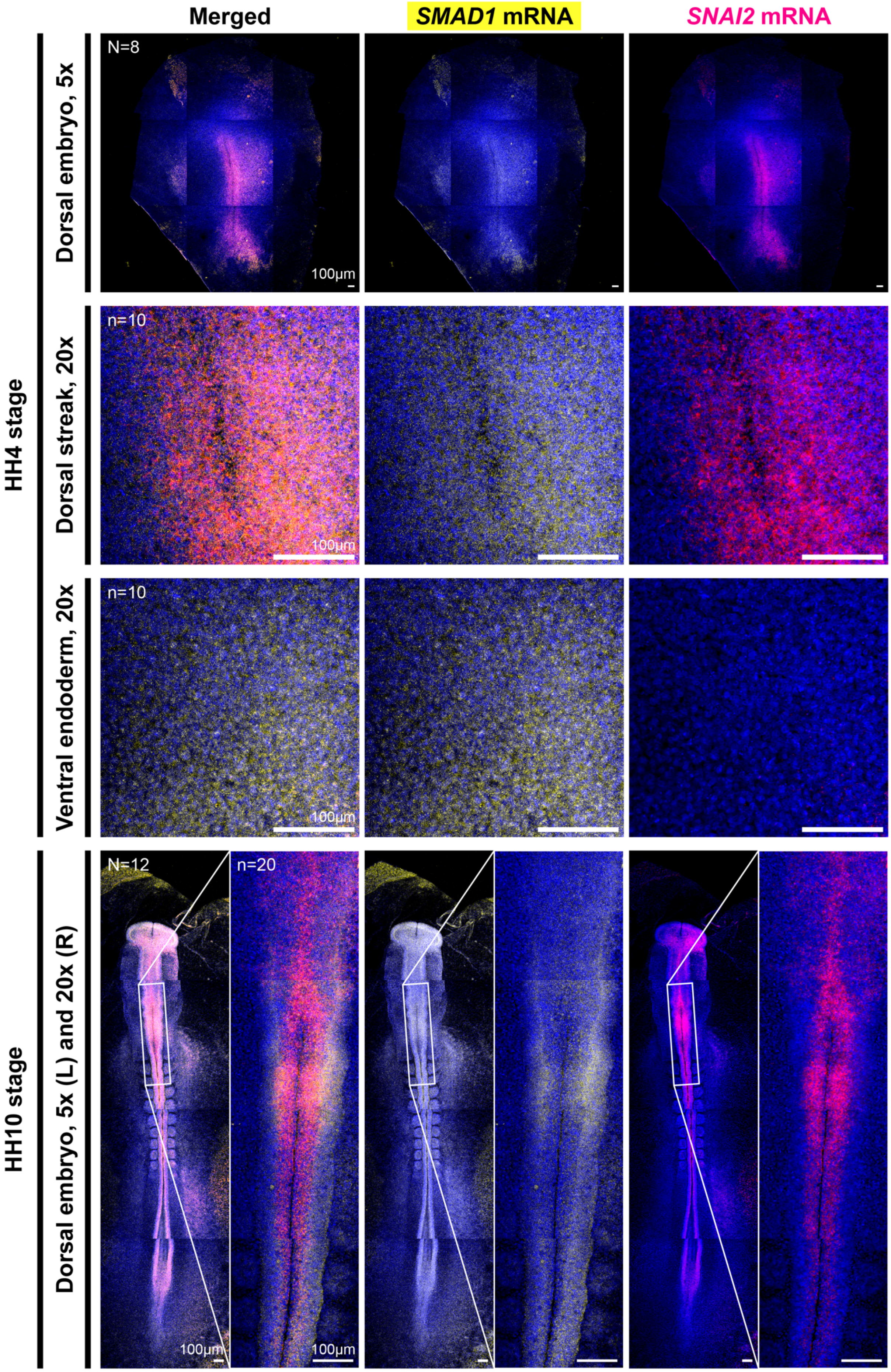

Merged
SMAD1 mRNA
SNAI2 mRNA
HH4 stage
Dorsal embryo, 5x
Dorsal streak, 20x
Ventral endoderm, 20x
HH10 stage
Dorsal embryo, 5x (L) and 20x (R)
N=8
n=10
n=10
N=12
n=20
100µm

Appendix Figure S14. Regions of *SNAI2* and *SMAD1* differential expression. Representative confocal images of *SNAI2* and *SMAD1* HCR staining in HH4 and HH10 embryos. In HH4 stage embryos, both the dorsal streak and ventral endoderm were imaged using the same microscope and FIJI image analysis parameters.

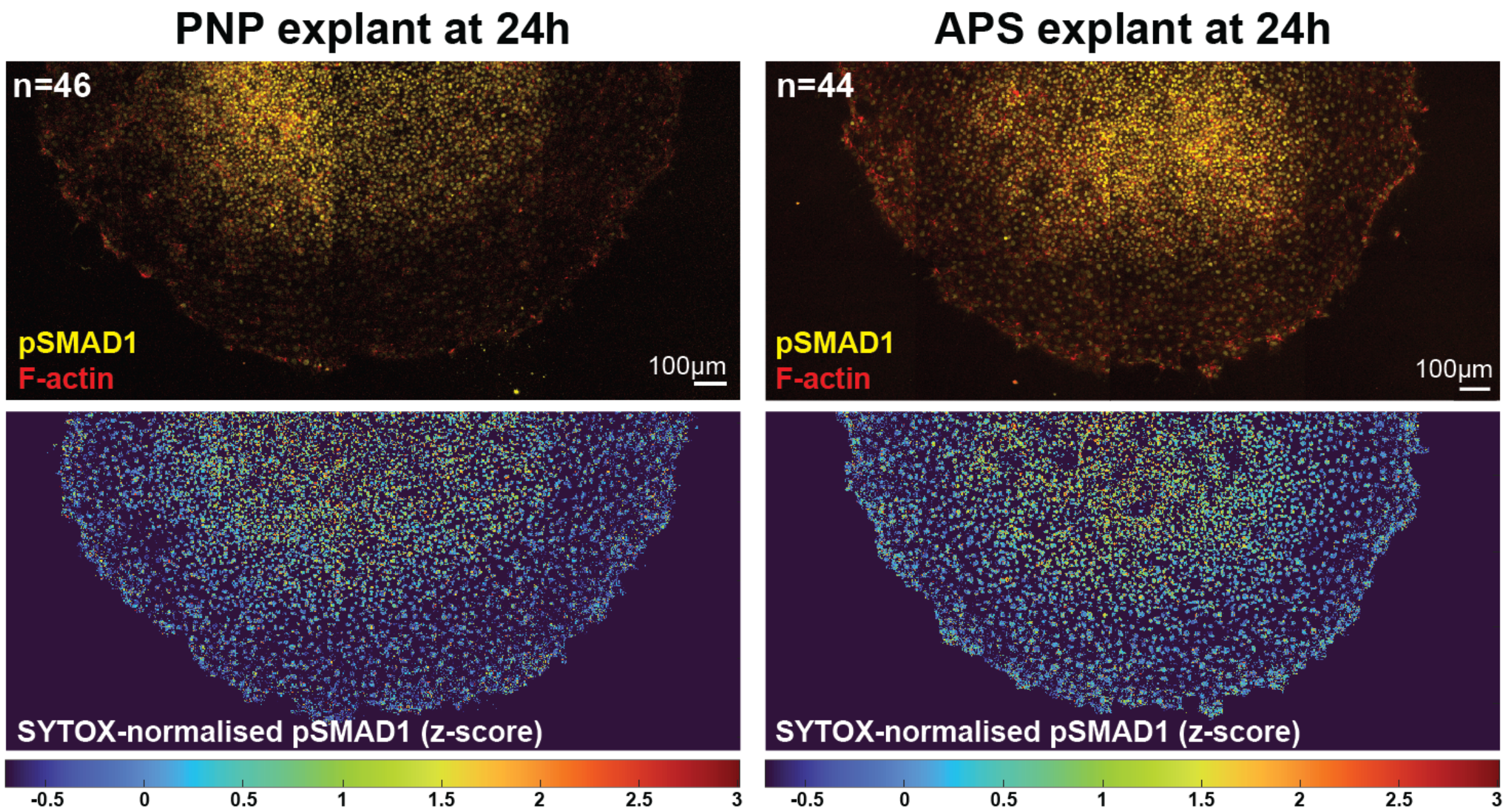

Appendix Figure S15. BMP signalling patterns in *ex vivo* explants. Top: Representative confocal images of pSMAD1 immunostaining and F-actin staining in PNP and APS explants on human fibronectin after 24h of incubation. Bottom: Z-scores of SYTOX-normalised pSMAD1 signal intensities. Z-scores tend to decrease towards the migrating edges.

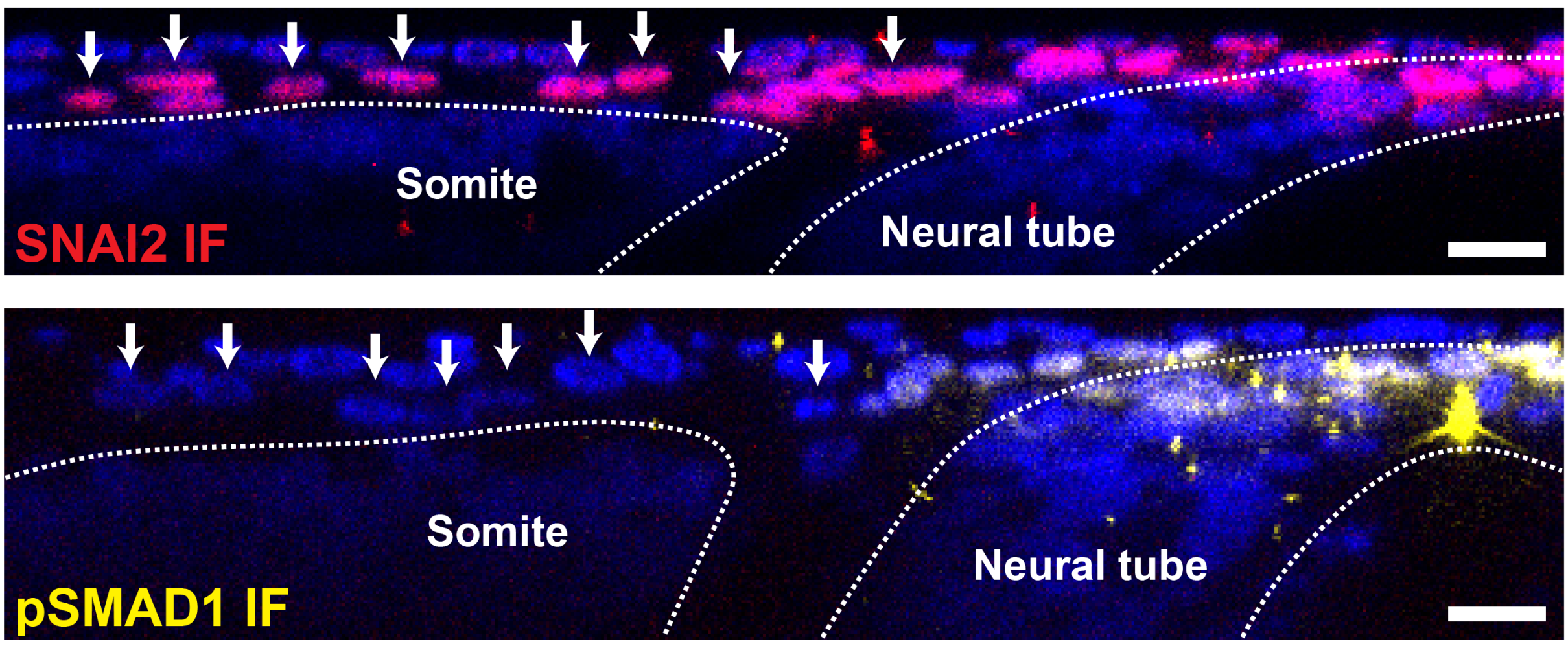

Appendix Figure S16. Reslices of dorsal neural tube and migrating neural crest cells. Top: Reslice of representative SNAI2 immunostaining. Bottom: Reslice of representative pSMAD1 immunostaining. White dotted lines show somite and neural tube boundaries.

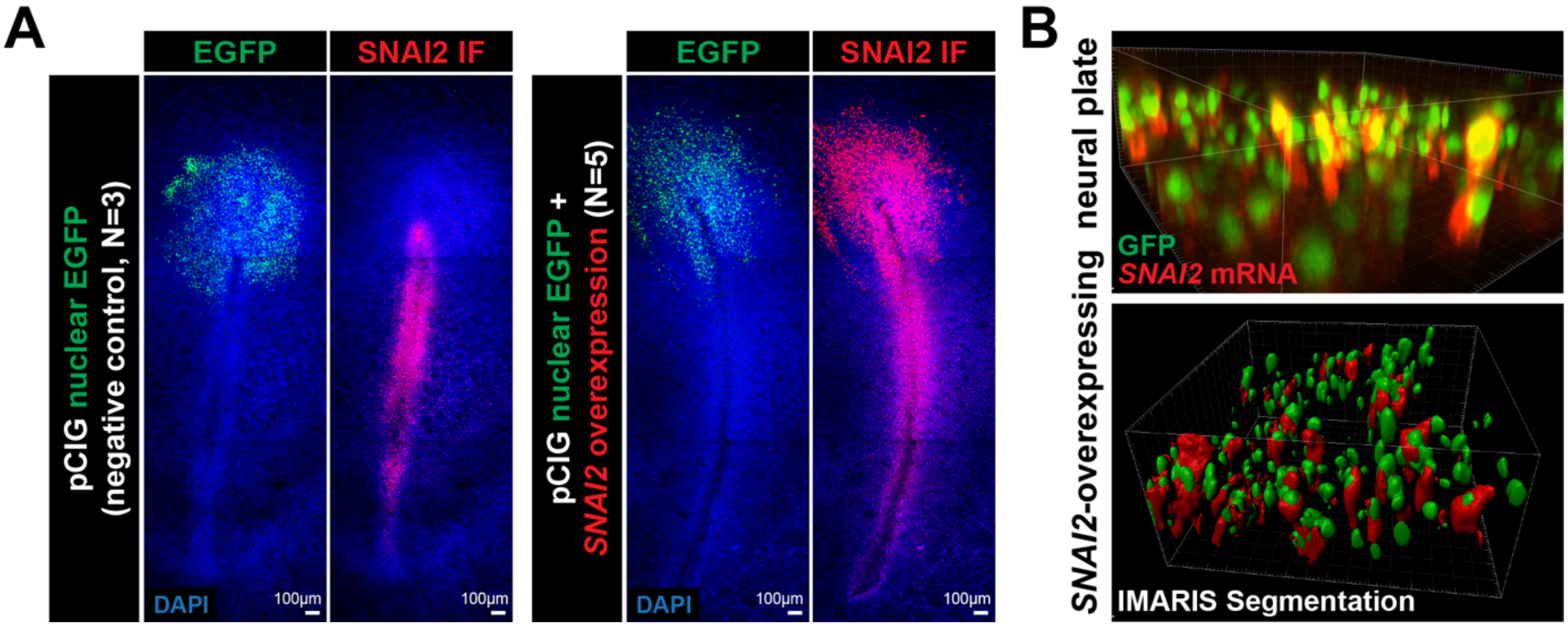

Appendix Figure S17. *SNAI2* overexpression validation. A) Representative SNAI2 immunofluorescence in pCIG vs pCIG-SNAI2 electroporated embryos. C) IMARIS 3D view snapshots of *SNAI2*-overexpressing PNP cells.

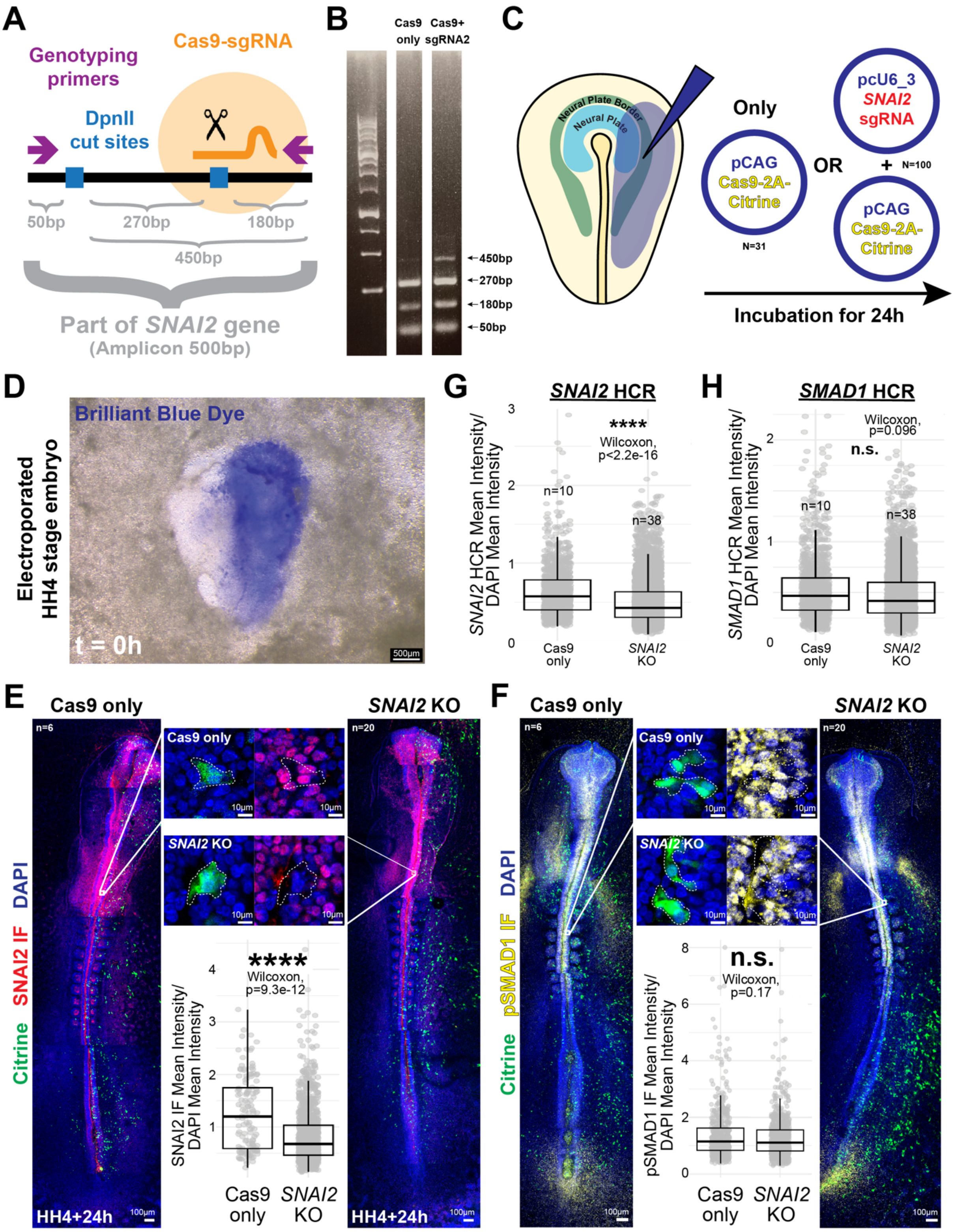

Appendix Figure S18. Insufficient knock out of *SNAI2* to test necessity hypothesis *in vivo*. A) Schematic of sgRNA design. B) Gel image showing an extra 450bp band in only the mosaic knockout lane. C) Schematic of CRISPR-Cas9 *SNAI2* knockout experiment in HH4 neural plate border. Embryos electroporated with pCAG Cas9-2A-Citrine (Cas9 expression vector) alone or with pcU6-3 SNAI2-sgRNA (*SNAI2* sgRNA expressing vector) and incubated for 24h. D) Representative brightfield image of an electroporated embryo at t=0h.

E) SNAI2 immunofluorescence in pCAG Cas9-2A-Citrine vs pCAG Cas9-2A-Citrine + pcU6-3 SNAI2-sgRNA electroporated embryos. IMARIS quantification of SNAI2 immunofluorescence signal intensities normalised by DAPI signal intensities in Citrine-positive cells. F) pSMAD1 immunofluorescence in pCAG Cas9-2A-Citrine vs pCAG Cas9-2A-Citrine + pcU6-3 SNAI2-sgRNA electroporated embryos. IMARIS quantification of pSMAD1 immunofluorescence signal intensities normalised by DAPI signal intensities in Citrine-positive cells. G) *SNAI2 in situ* HCR signal quantification in Citrine-positive cells. H) *SMAD1 in situ* HCR signal quantification in Citrine-positive cells.

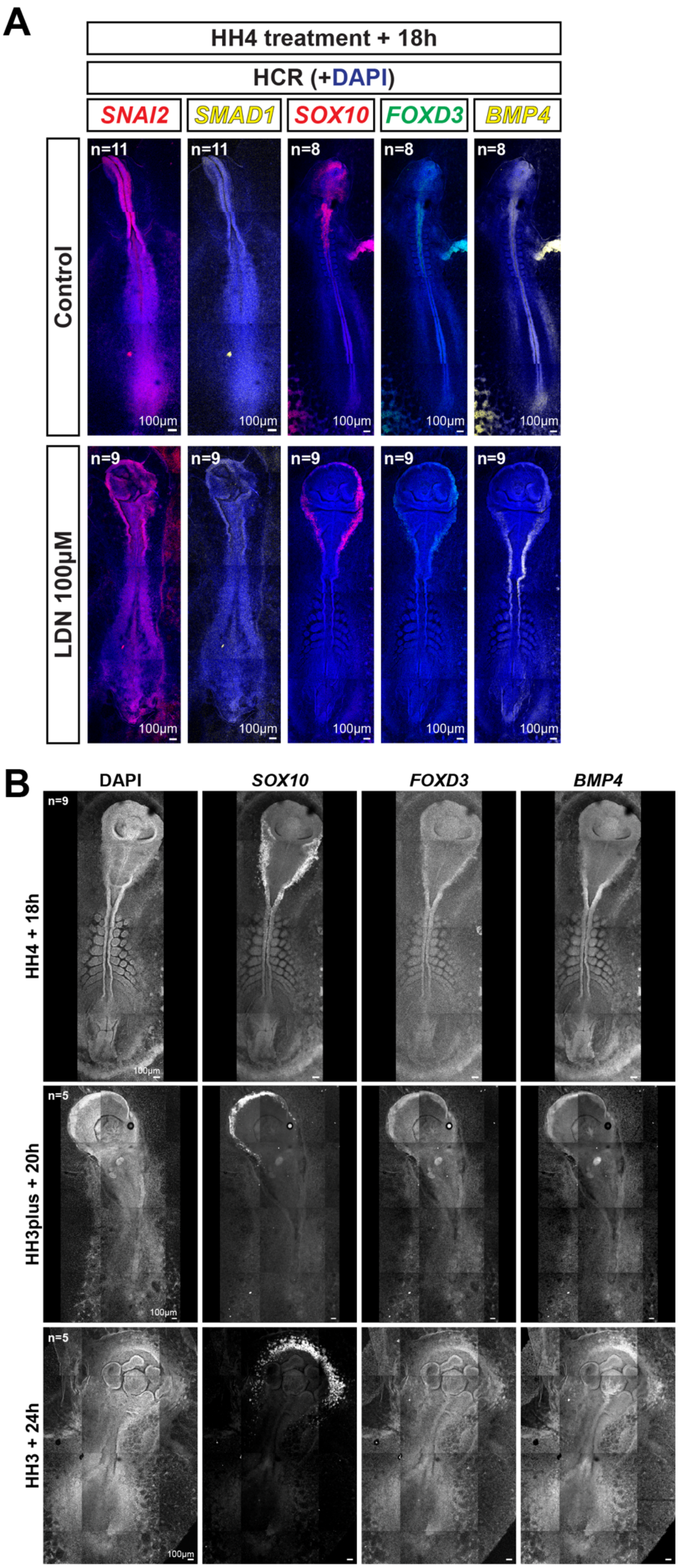
A
HH4 treatment + 18h
HCR (+DAPI)
SNAI2
SMAD1
SOX10
FOXD3
BMP4
Control
LDN 100µM
n=11
n=11
n=8
n=8
n=8
n=9
n=9
n=9
n=9
n=9
100µm
B
DAPI
SOX10
FOXD3
BMP4
HH4 + 18h
HH3plus + 20h
HH3 + 24h
n=9
n=5
n=5
100µm

Appendix Figure S19. Neural crest markers are still expressed in LDN-treated embryos. A) Representative confocal images of *SNAI2/SMAD1* and *SOX10/FOXD3/BMP4 in situ* HCR staining in control vs LDN-treated embryos. B) Representative greyscale confocal images of *SOX10/FOXD3/BMP4 in situ* HCR and DAPI staining of chick embryos treated with LDN from HH4 stage (top), HH3+ stage (middle), or HH3 stage (bottom).

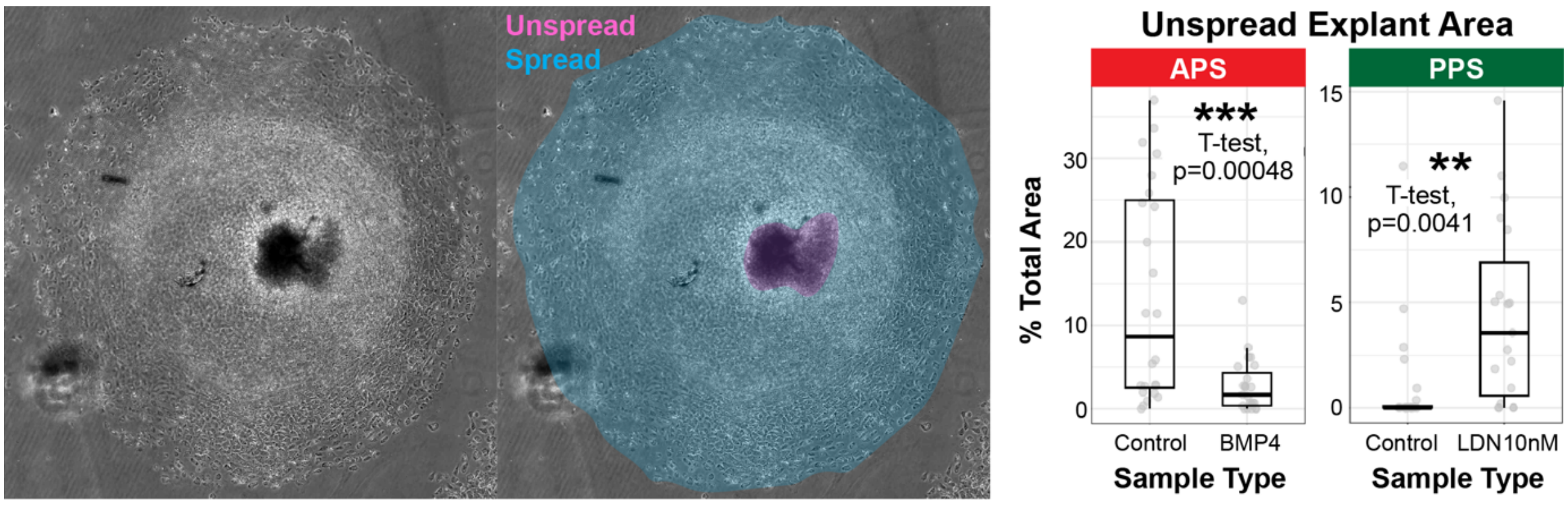

Appendix Figure S20. BMP signalling controls the extent of spreading in *ex vivo* explants. Left: Representative spread vs. unspread areas. Right: Quantification of explant area still left unspread at final time point.

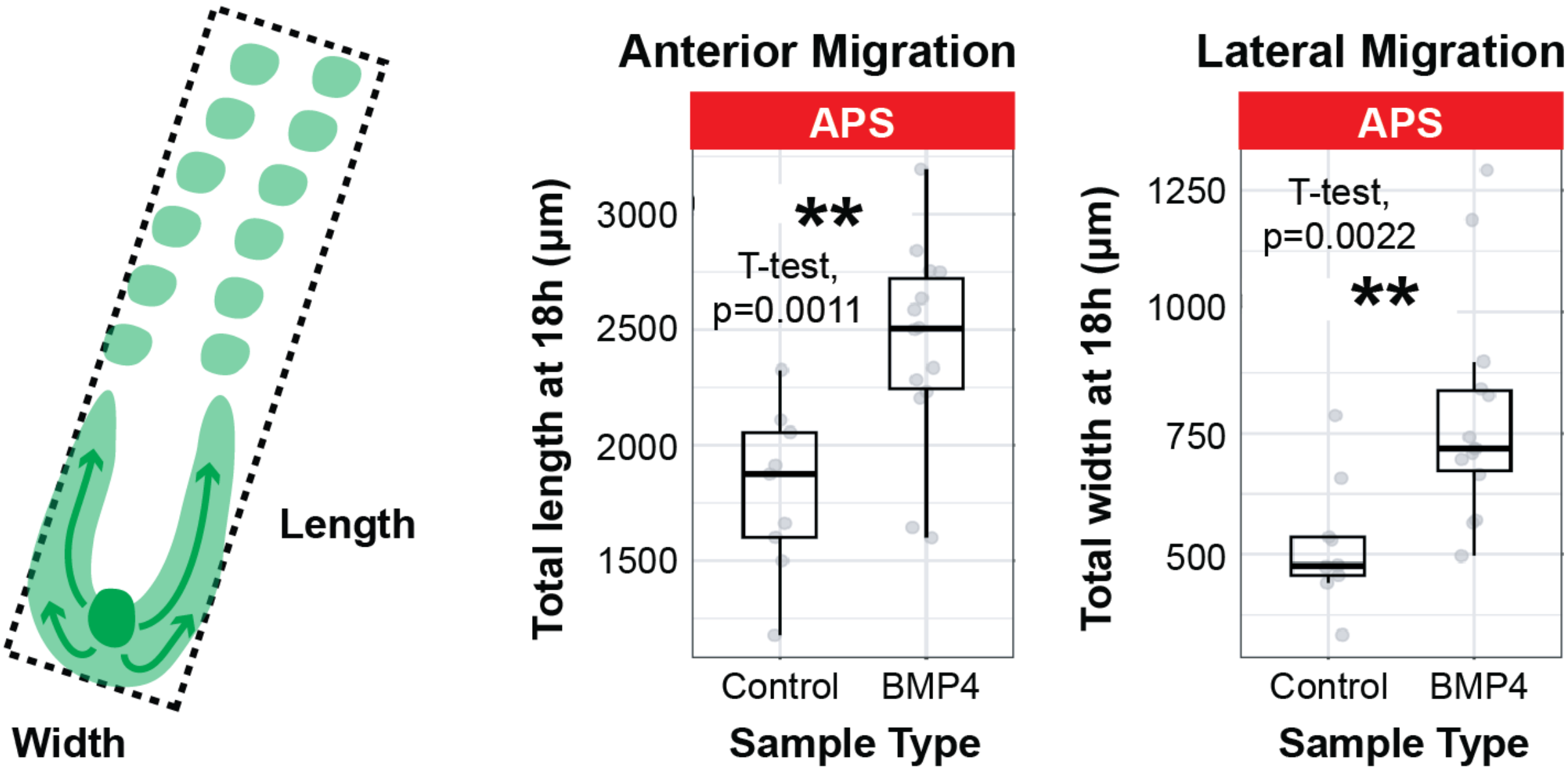

Appendix Figure S21. BMP4-treated APS grafts contribute more anteriorly and laterally compared to controls. Left: Schematic of graft length and width. Right: Quantification of APS graft anterior and lateral migration.
