# Supplementary material for: Reciprocal coupling between EMT and BMP morphogen signalling drives collective cell invasion
Source: EMBO J. 2026 Oct 7;45(21):7347–73. doi: 10.1038/s44318-026-00916-8 (PMC13728173; doi:10.1038/s44318-026-00916-8)
Supplement: Supplementary file 3 [file 44318_2026_916_MOESM2_ESM.pdf]

THE EMBO JOURNAL

Reciprocal coupling between EMT and BMP morphogen signalling drives collective cell invasion

Yuri Takahashi, Alexandra Neaverson, Lara Busby, Filip Twarowski, Carlos Camacho-Macorra, Guillermo Serrano Nájera, and Benjamin Steventon

Corresponding author: Benjamin Steventon (Bjs57@cam.ac.uk)

Review Timeline:

Submission Date:	17th Dec 25
Editorial Decision:	5th Mar 26
Revision Received:	25th Jun 26
Editorial Decision:	18th Aug 26
Revision Received:	28th Aug 26
Accepted:	9th Sep 26

Editor: Daniel Klimmeck

Transaction Report:

(Note: With the exception of the correction of typographical or spelling errors that could be a source of ambiguity, letters and reports are not edited. Depending on transfer agreements, referee reports obtained elsewhere may or may not be included in this compilation. Referee reports are anonymous unless the Referee chooses to sign their reports.)

Dear Dr Steventon,

Thank you again for the submission of your manuscript (EMBOJ-2025-123368-T) to The EMBO Journal, as well as for providing a preliminary response to the concerns raised by during peer-review. As mentioned earlier, your study was assessed by two reviewers with expertise in developmental biology, cell migration dynamics and related signaling, whose reports are enclosed below.

As you will see from their comments, the referees acknowledge the thorough ex vivo work on a relevant topic and potential interest of your findings on interdependence of EMT and BMP signaling during vertebrate developmental cell migration paradigms. However, they also express major concerns regarding overclaims and remaining conceptual gaps in the analysis, which dampen their enthusiasm. In more detail, reviewer #1 is concerned that definitive causal support for EMT as an upstream regulator of BMP signaling gradients via SNAI2 is not provided in his/her view (Ref#1, pt.1). This expert also states that the details of EMT-sensitive BMP signaling modalities and suggested feedback looping and likewise EMT initiation specifics both remain too prematurely explored (Ref#1, pts.2-4). Referee #2 agrees in that SNAI2-SMAD1 aligned functions and Snai2 causal roles in p-SMAD activation at EMT initiation zones is not sufficiently supported by the current data (Ref#2, pts.1,3,4). Further, the experts raise a number of issues related to the additional controls required, and statistics applied, that would need to be conclusively addressed to achieve the level of robustness and clarity needed for The EMBO Journal.

Given the overall interest stated and broader angle of your findings, we are able to invite you to revise your manuscript experimentally to address the referees' comments along the lines sketched in your response. However, please note that do require strong support from the referees on a revised version of the study in order to move on to publication of the work.

We generally allow three months as standard revision time. As a matter of policy, competing manuscripts published during this period will not negatively impact on our assessment of the conceptual advance presented by your study. However, we request that you contact the editor as soon as possible upon publication of any related work, to discuss how to proceed. Should you foresee a problem in meeting this three-month deadline, please let us know in advance and we may be able to grant an extension.

When submitting your revised manuscript, please carefully review the instructions below.

Please feel free to approach me any time should you have additional questions related to this.

Thank you for the opportunity to consider your work for publication.

I look forward to your revision.

Best regards,

Daniel Klimmeck

Daniel Klimmeck, PhD
Senior Editor
The EMBO Journal

Instruction for the preparation of your revised manuscript:

Read our guidance for manuscript revisions and related editorial policies: https://link.springer.com/journal/44318/submission-guidelines#cms-Revised-submissions
1) a .docx formatted version of the manuscript text (including legends for main figures, EV figures and tables). Please make sure

that the changes are highlighted to be clearly visible.

2) individual production quality figure files as .eps, .tif, .jpg (one file per figure).

3) a .docx formatted letter INCLUDING the reviewers' reports and your detailed point-by-point response to their comments. As part of the EMBO Press transparent editorial process, the point-by-point response is part of the Review Process File (RPF), which will be published alongside your paper.

4) a complete author checklist, which you can download from our author guidelines. Please insert information in the checklist that is also reflected in the manuscript. The completed author checklist will also be part of the RPF.

5) Please note that all corresponding authors are required to supply an ORCID ID for their name upon submission of a revised manuscript.

6) It is mandatory to include a 'Data Availability' section after the Materials and Methods. Before submitting your revision, primary datasets produced in this study need to be deposited in an appropriate public database, and the accession numbers and database listed under 'Data Availability'. Please remember to provide a reviewer password if the datasets are not yet public.
In case you have no data that requires deposition in a public database, please state so in this section. Note that the Data Availability Section is restricted to new primary data that are part of this study.
*** Note - All links should resolve to a page where the data can be accessed. ***

7) Our journal encourages inclusion of *data citations in the reference list* to directly cite datasets that were re-used and obtained from public databases. Data citations in the article text are distinct from normal bibliographical citations and should directly link to the database records from which the data can be accessed. In the main text, data citations are formatted as follows: "Data ref: Smith et al, 2001" or "Data ref: NCBI Sequence Read Archive PRJNA342805, 2017". In the Reference list, data citations must be labeled with "[DATASET]". A data reference must provide the database name, accession number/identifiers and a resolvable link to the landing page from which the data can be accessed at the end of the reference.

8) At EMBO Press we ask authors to provide source data for the main and EV figures. Our source data coordinator will contact you to discuss which figure panels we would need source data for and will also provide you with helpful tips on how to upload and organize the files.

Numerical data can be provided as individual .xls or .csv files (including a tab describing the data). For 'blots' or microscopy, uncropped images should be submitted (using a zip archive or a single pdf per main figure if multiple images need to be supplied for one panel.

9) We replaced Supplementary Information with Expanded View (EV) Figures and Tables that are collapsible/expandable online. A maximum of 5 EV Figures can be typeset. EV Figures should be cited as 'Figure EV1, Figure EV2" etc. in the text and their respective legends should be included in the main text after the legends of regular figures.

- For the figures that you do NOT wish to display as Expanded View figures, they should be bundled together with their legends in a single PDF file called *Appendix*, which should start with a short Table of Content. Appendix figures should be referred to in the main text as: "Appendix Figure S1, Appendix Figure S2" etc.

- Additional Tables/Datasets should be labelled and referred to as Table EV1, Dataset EV1, etc. Legends have to be provided in a separate tab in case of .xls files. Alternatively, the legend can be supplied as a separate text file (README) and zipped together with the Table/Dataset file.

10) When assembling figures, please refer to our figure preparation guideline in order to ensure proper formatting and readability in print as well as on screen:
https://media.springernature.com/original/springer-cms/rest/v1/content/27825798/data/v1

Please remember: Digital image enhancement is acceptable practice, as long as it accurately represents the original data and conforms to community standards. If a figure has been subjected to significant electronic manipulation, this must be noted in the figure legend or in the 'Materials and Methods' section. The editors reserve the right to request original versions of figures and the original images that were used to assemble the figure.

11) For data quantification: please specify the name of the statistical test used to generate error bars and P values, the number (n) of independent experiments (specify technical or biological replicates) underlying each data point and the test used to calculate p-values in each figure legend. The figure legends should contain a basic description of n, P and the test applied. Graphs must include a description of the bars and the error bars (s.d., s.e.m.).

Please remember: Digital image enhancement is acceptable practice, as long as it accurately represents the original data and conforms to community standards. If a figure has been subjected to significant electronic manipulation, this must be noted in the figure legend or in the 'Materials and Methods' section. The editors reserve the right to request original versions of figures and the original images that were used to assemble the figure.

Further information is available in our Guide For Authors: https://link.springer.com/journal/44318/submission-guidelines#cms-Revised-submissions

Please remember: Digital image enhancement is acceptable practice, as long as it accurately represents the original data and conforms to community standards. If a figure has been subjected to significant electronic manipulation, this must be noted in the figure legend or in the 'Materials and Methods' section. The editors reserve the right to request original versions of figures and the original images that were used to assemble the figure.

We realize that it is difficult to revise to a specific deadline. In the interest of protecting the conceptual advance provided by the work, we recommend a revision within 3 months (3rd Jun 2026). Please discuss the revision progress ahead of this time with the editor if you require more time to complete the revisions.

Referee #1:

This manuscript by Takahashi et al. addresses an important and timely question: how epithelial-mesenchymal transition (EMT), morphogen signalling, and collective invasion are coordinated during vertebrate development. By combining ex vivo explant systems with in vivo perturbations and grafting assays, the authors propose a conceptually appealing framework in which EMT initiation and BMP signalling are reciprocally coupled to coordinate cell fate specification and invasion dynamics. The experimental systems are appropriate, the datasets are extensive, and the downstream role of BMP signalling in regulating fate proportions and invasion dynamics is convincingly demonstrated. The study has clear potential to make a meaningful contribution to the EMT and developmental biology fields. However, several central conceptual claims-particularly regarding EMT as an upstream inducer of BMP signalling competence-would benefit from clarification or additional causal support.

Major Comments

1) A central conceptual issue concerns the interpretation of ECM-plated explants as a direct proxy for EMT initiation. Plating tissues on fibronectin or collagen induces broad migratory and mechanotransductive responses, in addition to EMT-associated transcriptional changes, making it difficult to attribute subsequent BMP pathway activation specifically to EMT rather than to ECM-induced migration or mechanical signalling. While SNAI2 induction indicates engagement of EMT-related programs, EMT is not experimentally isolated from other ECM-driven processes in this system.
The manuscript convincingly demonstrates that BMP signalling is dispensable for EMT initiation yet acts downstream to regulate cell fate proportions and invasion dynamics. This direction of the coupling is well supported. In contrast, evidence for the reverse direction-EMT initiation actively inducing or priming BMP signalling-is limited to correlation and SNAI2 gain-of-function experiments demonstrating sufficiency for SMAD1 transcription. Necessity is not tested: EMT is not experimentally attenuated, SNAI2 loss-of-function does not suppress BMP signalling, and mutant explants are not analysed for EMT progression or BMP responsiveness.
Overall, the data support a permissive association between EMT-associated programs and BMP signalling rather than a demonstrated reciprocal causal loop. Partial attenuation of EMT followed by assessment of BMP responsiveness would strengthen this conclusion; alternatively, moderating the language to describe EMT as associated with or permissive for BMP competence would better align the claims with the data.

2) The concept of "BMP pathway competence" is central to the manuscript but remains insufficiently operationally defined. It is unclear whether competence refers to increased SMAD1 expression, enhanced sensitivity to BMP ligands, altered receptor engagement, sustained nuclear pSMAD1 signalling, or increased transcriptional output. While SMAD1 upregulation is clearly demonstrated, functional responsiveness to defined BMP inputs is not directly tested.
Neither EMT induction nor SNAI2 overexpression is shown to activate pSMAD1 or canonical BMP target genes, nor is enhanced responsiveness to exogenous BMP demonstrated. Although BMP pathway activity is inferred from pSMAD1 staining and shown to be sensitive to BMP receptor inhibition, it remains unclear whether EMT initiation or SNAI2/SMAD1 upregulation alters ligand-receptor coupling or instead expands intracellular capacity to transduce BMP signals once receptors are activated. Moreover, SNAI2 overexpression is mosaic, yet BMP signalling is not assessed at single-cell resolution in SNAI2-positive cells, leaving unresolved whether SMAD1 induction translates into functional BMP pathway activation in the same cells. As such, BMP

competence is inferred rather than directly measured. A minimal functional assay-quantifying pSMAD1 or BMP target induction in response to a defined BMP4 pulse before and after EMT attenuation-would substantially strengthen the mechanistic interpretation.

3) A further concern relates to how EMT initiation is defined and staged in each of the PPS and APS explant model. Plating tissues on fibronectin or collagen elicits broad migratory and mechanotransductive responses, including integrin signalling, cytoskeletal remodelling, and cell spreading, in addition to EMT-associated transcriptional changes. EMT initiation is therefore inferred rather than directly demonstrated, and it remains unclear whether the explants represent a defined early EMT state or a heterogeneous mixture of epithelial, partial EMT, and post-EMT migratory cells.
Consistent with this, pSMAD1 activity is highest in central, epithelial regions of explants and decreases toward the migratory front (Fig. 1H, Fig. S5, Fig. S9), where cells display the most pronounced mesenchymal morphology. Thus, BMP signalling appears restricted to early or pre-migratory states rather than sustained in actively migrating cells, which is not fully reconciled with claims that EMT induction itself activates BMP signalling.
Early temporal and spatial characterization of EMT markers in APS and PNP explants, together with correlation of EMT stage and pSMAD1 localization, would strengthen the interpretation that EMT initiation-rather than ECM-induced migration per se-primes BMP signalling competence.

4) EMT initiation is primarily inferred from SNAI2 expression, loss of polarity markers, and early transcriptional changes. However, the depth, heterogeneity, and maturation of EMT states are not explored. Canonical mesenchymal markers (e.g. vimentin, FN1, integrins) are not clearly shown or discussed in the explant system, and migration is analysed largely at the level of bulk explant spreading. Given the extensive literature on partial and context-specific EMT states, it would be important to clarify whether APS and PNP explants undergo comparable or distinct EMT programs, and whether differences in cell migration dynamics reflect EMT state, fate specification, or tissue geometry; authors are also invited to discuss this in the discussion.

Specific Comments
1. SNAI2 expression at the 0 h explant time point is inferred from in vivo embryos and bulk RNA-seq but is not directly visualized in freshly dissected explants.
2. pSMAD1 is interpreted as nuclear, but nuclear counterstains and proper quantifications (e.g. nuc/cyto ratio) in situ are not shown. Reduced pSMAD1 at the explant periphery could reflect out-of-plane nuclei rather than true signalling differences.
3. While SNAI2 overexpression is sufficient to induce SMAD1 transcription, the authors do not demonstrate that SNAI2 overexpression induces EMT in this context, nor that EMT is required for SMAD1 phosphorylation or BMP pathway activation. Thus, the observed SMAD1 upregulation may reflect a direct transcriptional effect of SNAI2 rather than an EMT-dependent increase in BMP signalling competence.
4. More detailed molecular and dynamic characterization of the APS and PNP explant models would strengthen interpretation of EMT state, heterogeneity, and collective migration mechanisms underlying the reported BMP-dependent invasion phenotypes.-
5. Am important test would be SNAI2 overexpression {plus minus} LDN, followed by pSMAD1/BMP target readouts in EGFP-positive cells, to establish whether SNAI2-driven SMAD1 expression translates into BMP receptor-dependent signalling.
6. SMAD1 phosphorylation can occur downstream of non-BMP inputs (e.g. TGFβ). Additional pathway dissection would strengthen attribution to BMP signalling.
7. BMP inhibition relies on a single inhibitor (LDN193189). Confirmation with an independent inhibitor or ligand neutralization would strengthen specificity.
8. In the explant system, the EMT→BMP direction could be tested with a minimal BMP competence assay: partially attenuate EMT onset and quantify nuclear pSMAD1 and BMP target induction in response to a defined BMP4 pulse ({plus minus} LDN control).
9. While BMP signalling clearly modulates tissue-scale dispersal downstream of EMT, the underlying cell-level effects remain insufficiently resolved. It is unclear whether BMP acts directly on migratory behaviour or indirectly through fate allocation. This could be addressed by single-cell tracking combined with temporal BMP perturbations.
10. Terms such as "EMT initiation zones," "BMP competence," and "collective invasion dynamics" would benefit from explicit operational definitions or moderated phrasing.

Referee #2:

In this study, the authors take advantage of the chicken embryo and its accessibility to two models of collective cell migration-gastrulation and neural crest-to investigate the interplay between EMT initiation and morphogen signaling during morphogenesis. They show that the anterior primitive streak and the prospective neural plate, when cultured on fibronectin, spread and acquire a molecular signature of EMT. Interestingly, this process is accompanied by an upregulation of BMP members and signaling activity.
The authors then demonstrate a correlation between Smad1 and Snail2 expression and show that Snail2 is able to activate Smad1 when overexpressed. This identifies a molecular mechanism linking Smad signaling to the EMT process. Finally, they show that Smad1 signaling is active at embryonic EMT sites, but that it is not required for EMT initiation per se; rather, it plays a role in cell fate (as previously known) and in cell spreading and collective migration.

Overall, this paper is very interesting, as it identifies a new link between BMP/Smad signaling and the EMT process during embryogenesis. This work could represent a cornerstone for understanding how signaling pathways coordinate morphogenetic movements and fate decisions. The study is generally well performed, the data are convincing, and the technical aspects are well described. Although the manuscript is sometimes difficult to follow due to the transitions between different experimental systems, I believe it is very interesting and could be a good fit for EMBO Journal. However, several aspects need to be addressed before I can fully endorse its publication.

Major comments

1) I am troubled by the fact that HCR staining against Smad1 and SNAIL2 appears to be almost perfectly overlapping (Fig. 2D, Fig. S13A), including what looks like extraembryonic, potentially nonspecific staining. I understand that the authors have controlled for bleed-through, but could there be issues related to the amplification steps? Would it be possible to show some degree of differential expression in a wild-type context, as was done in the gain-of-function experiments (Fig. S11B)? According to the single-cell data, this should be feasible.
2) The role of Smad signaling in spreading and collective invasion is claimed for the primitive streak. Is this also the case for the other system (PNP/NC)? Based on Fig. S14, this is not clear. Could the authors provide quantification of neural crest migration in LDN-treated embryos and/or quantification of PNP explant spreading under LDN conditions?
3) A key result supporting the idea that EMT primes BMP pathway competence is that the PNP-where there is no Snail2, no Smad1, and no phospho-Smad1-undergoes EMT, Snail2 induction, and p-Smad1 activation when cultured on fibronectin, whereas , in contrast, when PNP is cultured in liquid (where EMT is presumably absent), Smad1 phosphorylation is not observed. In parallel APS explants in liquid show p-Smad1 activation, presumably because EMT factors such as Snail2 are already present and can upregulate Smad1 phosphorylation (likely due to local BMP4 signaling after isolation from BMP inhibitors).
First, I think these data (liquid culture) should be included in the main figures rather than the supplemental material. Second, I wonder whether PNP explants survive and/or differentiate as efficiently in liquid culture as APS explants or PNP explants on fibronectin. In Fig. S6B, the PNP explant appears smaller than the APS explant at 24 hours. This control seems important to support the conclusion that EMT itself primes BMP pathway activation.
4) From Fig. 3C, it is not entirely clear that Snail2 could induces Smad1 and subsequent p-Smad1 activation specifically at EMT initiation sites, as Snail2 expression does not appear particularly strong in the EMT initiation zone. Based on the proposed model, I would expect higher Snail2 expression in the epiblast. Could the authors provide higher-resolution imaging data to clarify this point?

Minor comments
• The statement that Snail2 expression precedes Smad1 expression at the end of the fifth paragraph of the Results section is not clearly supported by in vivo data.
• Most of Figure 4 (expect panel D and E) mainly appear as control experiments that could be moved to the supplementary material if needed.
• Some supplementary figures contain a large amount of data that is difficult to read. Several could be simplified by showing fewer representative examples or more synthetic presentations (e.g., Figs. S4, S7, S11).
• In the graphical abstract, I suggest using an arrow to indicate temporal progression rather than the distinction between "mechanism" and "response," which does not add much to the overall message.
• One potentially confusing aspect is that the APS, although it does not show p-Smad1 signaling, undergoes massive EMT to form paraxial mesoderm. Even though its spreading behavior appears weaker than that of PPS and can be enhanced by BMP4 (Fig. 6B), this raises the question of what drives collective behavior in the anterior primitive streak if not BMP signaling-perhaps FGF or Wnt pathways? I think this point should be addressed or discussed.

Response to reviewers highlighted in BLUE
Changes implemented in ORANGE

Referee #1:

This manuscript by Takahashi et al. addresses an important and timely question: how epithelial-mesenchymal transition (EMT), morphogen signalling, and collective invasion are coordinated during vertebrate development. By combining ex vivo explant systems with in vivo perturbations and grafting assays, the authors propose a conceptually appealing framework in which EMT initiation and BMP signalling are reciprocally coupled to coordinate cell fate specification and invasion dynamics. The experimental systems are appropriate, the datasets are extensive, and the downstream role of BMP signalling in regulating fate proportions and invasion dynamics is convincingly demonstrated. The study has clear potential to make a meaningful contribution to the EMT and developmental biology fields. However, several central conceptual claims-particularly regarding EMT as an upstream inducer of BMP signalling competence-would benefit from clarification or additional causal support.

Major Comments
1) A central conceptual issue concerns the interpretation of ECM-plated explants as a direct proxy for EMT initiation. Plating tissues on fibronectin or collagen induces broad migratory and mechanotransductive responses, in addition to EMT-associated transcriptional changes, making it difficult to attribute subsequent BMP pathway activation specifically to EMT rather than to ECM-induced migration or mechanical signalling. While SNAI2 induction indicates engagement of EMT-related programs, EMT is not experimentally isolated from other ECM-driven processes in this system.

We agree with the reviewer here and have modified the text make it clear that the assay has been used to induce cell migration rather than EMT specifically, through which we then uncover mechanistic links between SNAI2 and BMP signalling, via SMAD1 regulation.

Key changes made to concluding statements:

Highlight 1: "*Substrate-induced cell migration* activates BMP signalling induces migratory cell states".

Abstract: "Using substrate-induced *migration* in ex vivo explants, we demonstrate..

Introduction, 3rd paragraph: "To investigate potential reciprocal coupling between EMT initiation and morphogen signalling, we employed an experimental system to induce *cell migration* independently of developmental context..".

Figure 1 title: "Ex vivo *cell migration* assay reveals BMP activation and BMP-induced cell fate changes."

Results sub-section 1 title: "Ex vivo induction of *cell migration* in chick tissue explants"

Results, first paragraph: “This experimental system therefore provides a platform to determine the transcriptional events as cells *initiate cell migration* independently of other developmental cues”.

Results sub-section 2 title: “Ex vivo induction of *cell migration* drives convergent cell fate shifts toward migratory *cell states”*

Results sub-section 2, first paragraph summary: “..suggesting that while *induced cell migration* drives a shared programme, distinct lineage identities continued to specify and diverge”.

Results sub-section 2, final sentence: “Together, these findings demonstrate that substrate-induced *cell migration* triggers cell fate responses across germ layers”.

Results sub-section 3 title: “Ex vivo induction of *cell migration* triggers BMP pathway activation”
Results sub-section 3 conclusion: “Together, these results demonstrate that *substrate-induced cell migration* triggers both BMP pathway component expression and signalling activity, consistent with the observed cell fate changes toward migratory lineages. *Furthermore, the rapid upregulation of SNAI2 expression supports a role for this transcription factor as an upstream activator of this switch*”.

The manuscript convincingly demonstrates that BMP signalling is dispensable for EMT initiation yet acts downstream to regulate cell fate proportions and invasion dynamics. This direction of the coupling is well supported. In contrast, evidence for the reverse direction-EMT initiation actively inducing or priming BMP signalling-is limited to correlation and SNAI2 gain-of-function experiments demonstrating sufficiency for SMAD1 transcription. Necessity is not tested: EMT is not experimentally attenuated, SNAI2 loss-of-function does not suppress BMP signalling, and mutant explants are not analysed for EMT progression or BMP responsiveness.
Overall, the data support a permissive association between EMT-associated programs and BMP signalling rather than a demonstrated reciprocal causal loop. Partial attenuation of EMT followed by assessment of BMP responsiveness would strengthen this conclusion; alternatively, moderating the language to describe EMT as associated with or permissive for BMP competence would better align the claims with the data.

We thank the reviewer for this suggestion, as we feel that has helped clarify the overall message of the paper regarding the mechanistic connection between SNAI2 and SMAD1. Our previous data showed that SNAI2 loss-of-function in vivo did not lead to significant changes in smad1 expression, or pSMAD1 activity within single cells (Fig. S22). However, we reasoned that this may be due to SNAI2 acting redundantly with additional signals to modulate BMP signalling in vivo. As suggested by the reviewer, we analysed mutant explants for smad1 expression and pSMAD1 activity- in the presence and absence of BMP4. Importantly, we observed a downregulation of their expression levels when SNAI2 is reduced, irrespective of whether or not BMP4 is present (see revised Fig. 4). This allows us to confirm a functional requirement of SNAI2 expression for smad1 expression and BMP signalling. However, this has also led us to revise our conclusion about BMP competence, and to conclude directly about an impact of BMP activity instead. Therefore, we have altered our conclusions relating to ‘BMP competence’, to ‘BMP activity’ throughout the manuscript.

Key changes made to concluding statements:

Highlights 2: “SNAI2 is both sufficient *and required* for SMAD1 expression and BMP pathway *activity”*.

Abstract:
Ex vivo CRISPR-Cas9 experiments further demonstrate that SNAI2 is required for SMAD1 expression and BMP signalling *activity*.

Final conclusion “This coupling provides a mechanism for synchronising cell fate specification with invasion progression during axis elongation, positioning EMT as a process that actively modulates BMP activity to coordinate tissue-level cell behaviours during collective cell invasion”.

Introduction: “In vivo and ex vivo functional studies demonstrated that SNAI2 overexpression is sufficient and required for SMAD1 expression and BMP signalling activity”.

Sub-section 4 title: “SMAD1 upregulation couples EMT initiation to BMP signalling *activity”.*

New results section title:"SNAI2 expression is required for SMAD1 expression and BMP signalling activity ex vivo”

New results section text:

“…

To test this in a more controlled setting, we dissected APS explants from SNAI2 CRISPR and control embryos and cultured these in either free-floating or fibronectin-plated conditions, treating each with or without exogenous BMP4 protein (Fig. 4D). This ex vivo approach allowed us to assess BMP response directly while controlling for the complexity of the intact embryonic environment. In both conditions, SNAI2 CRISPR explants showed significantly reduced SNAI2 transcript levels compared to controls, confirming effective editing (Fig. 4E,H).

SNAI2 CRISPR cells also showed significantly reduced SMAD1 transcript levels in both free-floating (Fig. 4F) and spreading (Fig. 4I) conditions, with spreading CRISPR+BMP4 cells additionally showing a significantly higher proportion of cells at low SMAD1 expression levels. At the level of BMP pathway activation, SNAI2 CRISPR cells showed significantly reduced pSMAD1 protein levels in migrating explants (Fig. 4J). In free-floating explants, where substrate contact is absent, SNAI2 CRISPR cells showed a significantly lower high-pSMAD1 peak in BMP4-treated cells compared to controls (Fig. 4G). Notably, the reduction in pSMAD1 caused by SNAI2 loss persisted even in the presence of exogenous BMP4 (Fig. 4G,J), indicating that SNAI2 is required for BMP pathway activation regardless of BMP ligand availability. Together, these results demonstrate that SNAI2 is required for SMAD1 expression and BMP signalling activity ex vivo.”

New discussion text and revised heading in section “EMT initiation triggers BMP pathway *activity* to coordinate collective cell invasion”: “Ex vivo loss-of-function experiments further demonstrate that

SNAI2 is required for SMAD1 expression and BMP signalling, with SNAI2 CRISPR cells showing significantly reduced SMAD1 transcript levels and a dampened pSMAD1 response to exogenous BMP4 in both free-floating and fibronectin-plated conditions. We note that in vivo CRISPR experiments did not resolve this necessity relationship, likely reflecting the role of additional mechanisms impacting BMP activity in vivo.

2) The concept of "BMP pathway competence" is central to the manuscript but remains insufficiently operationally defined. It is unclear whether competence refers to increased SMAD1 expression, enhanced sensitivity to BMP ligands, altered receptor engagement, sustained nuclear pSMAD1 signalling, or increased transcriptional output. While SMAD1 upregulation is clearly demonstrated, functional responsiveness to defined BMP inputs is not directly tested.
Neither EMT induction nor SNAI2 overexpression is shown to activate pSMAD1 or canonical BMP target genes, nor is enhanced responsiveness to exogenous BMP demonstrated. Although BMP pathway activity is inferred from pSMAD1 staining and shown to be sensitive to BMP receptor inhibition, it remains unclear whether EMT initiation or SNAI2/SMAD1 upregulation alters ligand-receptor coupling or instead expands intracellular capacity to transduce BMP signals once receptors are activated.

As mentioned above, we observe a reduction in p-SMAD1 whether in response to SNAI2 loss-of-function whether or not BMP4 is present, demonstrating a direct requirement for SNAI2 for BMP signalling activity, rather than BMP competence per se. As such, we have adjusted these conclusions throughout the manuscript. We don’t feel that this has limited the impact of the work, which is centred around the concept of reciprocal interactions enabling collective cell invasion. Instead, the new data added in response to experiment suggested by this reviewer has strengthened the reverse causal connection between SNAI2 (typically seen as acting downstream of BMP controlled cell fate regulation) and BMP signalling activity, via Smad1 expression. It has also allowed us to clarify our conclusions in this regard.

Moreover, SNAI2 overexpression is mosaic, yet BMP signalling is not assessed at single-cell resolution in SNAI2-positive cells, leaving unresolved whether SMAD1 induction translates into functional BMP pathway activation in the same cells. As such, BMP competence is inferred rather than directly measured. A minimal functional assay-quantifying pSMAD1 or BMP target induction in response to a defined BMP4 pulse before and after EMT attenuation-would substantially strengthen the mechanistic interpretation.

For the SNAI2 overexpression experiment in figure 4, we performed single cell segmentation and quantification of expression levels for SNAI2 and SMAD1 mRNA, as our primary goal here was to assess whether cells that have access SNAI2 also result in higher levels of SMAD1 transcription. This was observed for a large proportion (Fig 4C; yellow bar; n=308 cells), however a significant number showed overexpression of SNAI2 only (Fig 4C; red bar; n=651 cells). This is likely due to the inherent variability in the expression levels of electroporated plasmids that is unavoidable in our system. Nevertheless, this is a significant increase in the number of SMAD1+/SNAI2+ cells in controls, which is zero when the expression plasmid alone is introduced.

For the new SNAI2 loss-of-function experiments, similar single-cell quantifications have been performed and demonstrate a significant reduction in both SMAD1 expression and p-SMAD1 staining (as a read-out of BMP activity) when cells receive both Cas9 and SNAI2 guide RNAs.

3) A further concern relates to how EMT initiation is defined and staged in each of the PPS and APS explant model. Plating tissues on fibronectin or collagen elicits broad migratory and mechanotransductive responses, including integrin signalling, cytoskeletal remodelling, and cell spreading, in addition to EMT-associated transcriptional changes. EMT initiation is therefore inferred rather than directly demonstrated, and it remains unclear whether the explants represent a defined early EMT state or a heterogeneous mixture of epithelial, partial EMT, and post-EMT migratory cells.

Consistent with this, pSMAD1 activity is highest in central, epithelial regions of explants and decreases toward the migratory front (Fig. 1H, Fig. S5, Fig. S9), where cells display the most pronounced mesenchymal morphology. Thus, BMP signalling appears restricted to early or pre-migratory states rather than sustained in actively migrating cells, which is not fully reconciled with claims that EMT induction itself activates BMP signalling.
Early temporal and spatial characterization of EMT markers in APS and PNP explants, together with correlation of EMT stage and pSMAD1 localization, would strengthen the interpretation that EMT initiation-rather than ECM-induced migration per se-primes BMP signalling competence.

Our interpretation is that the higher levels of pSMAD1 activity within the central region of the explants marks sites where EMT is being initiated, reflecting the co-incidence of these processes in the embryo (Figures 3, S9, S10). As cells spread outwards, BMP signalling levels reduce, again reflecting the lower levels of pSMAD1 staining in migratory cells *in vivo* (Figures 3, S9, S10). To validate these interpretations, we have performed a pseudo time-course analysis of SNAI2, *SMAD1* expression and pSMAD1 activity from 0 hours plating up to 24 hours. These results are now included as a series of new supplementary figures (Figs. S6-S11), and summarise in Fig. S12, that is reproduced here:

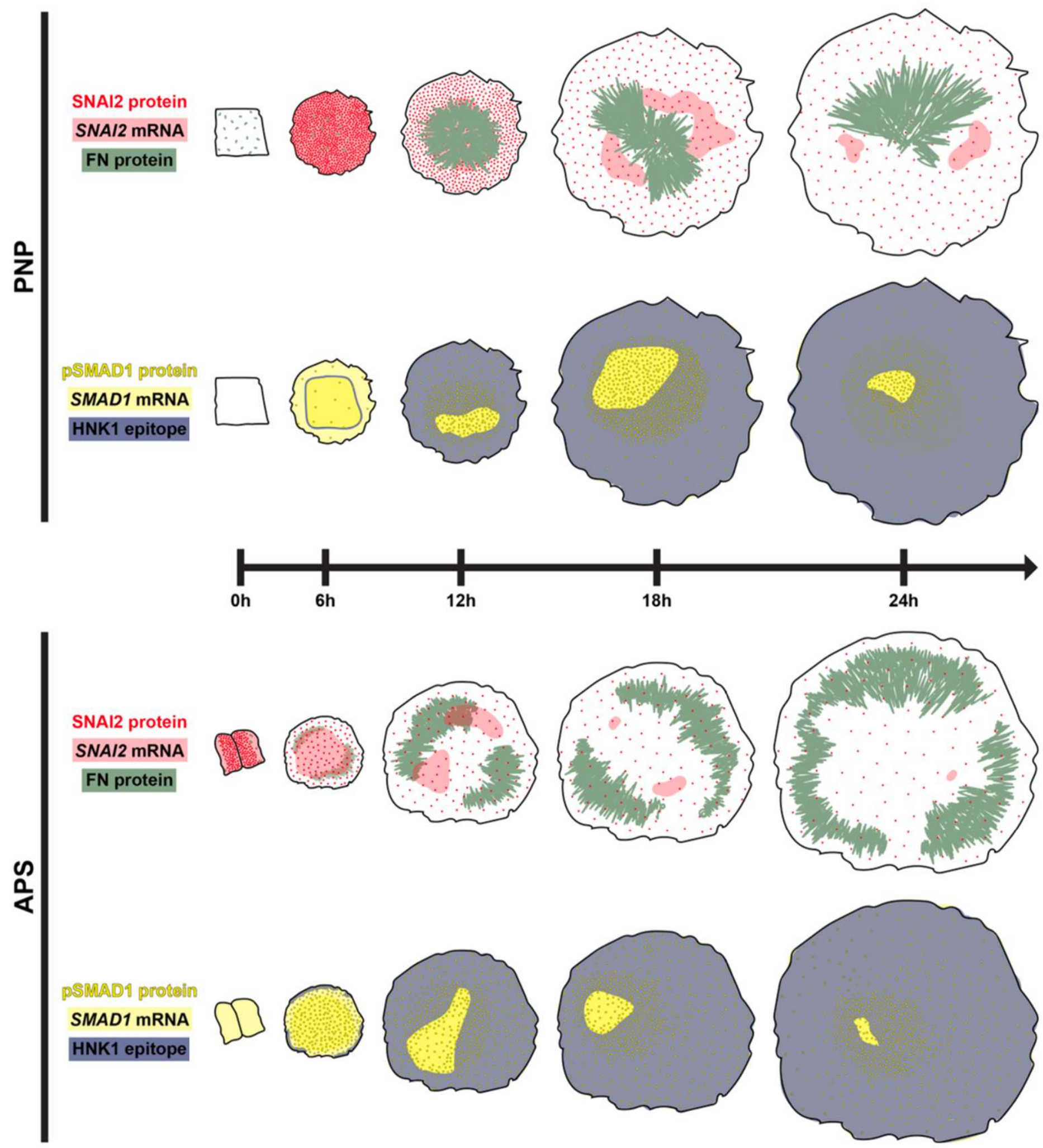

Fig. S12. Summary of pseudo time-course analysis of markers. Schematic of observed expression of SNAI2 protein, *SNAI2* mRNA, FN protein, pSMAD1 protein, *SMAD1* mRNA, and HNK1 epitope in PNP and APS migrating explants at multiple timepoints.

This additional data has allowed us to better clarify the spatial and temporal progression of events as described in the following text addition:

1) In "Ex vivo induction of cell migration triggers BMP pathway activation":

To directly characterise the temporal upregulation of SNAI2 and its and their relationship to BMP pathway activation in this system, we performed pseudo timecourse analyses of PNP and APS explants fixed at 0, 6, 12, 18, and 24 hours of culture on fibronectin and stained for SNAI2 mRNA, SNAI2 protein, pSMAD1 protein, fibronectin (FN) protein, and the carbohydrate HNK1 epitope (Fig. S6-12). In PNP explants, both SNAI2 mRNA and SNAI2 protein were absent at 0 hours (Fig. S8 & S7), thus confirming the pre-EMT state of freshly dissected PNP tissue. SNAI2 mRNA appeared broadly across the explant by 6 hours of culture, before becoming progressively restricted to patches of expression as cells spread outward over sub sequent timepoints (Fig. S8). SNAI2 protein also appeared robustly by 6 hours of culture (Fig. S7). Together, this demonstrated the rapid induction of

SNAI2 expression upon substrate contact. As for BMP signalling, pSMAD1 protein was absent at 0 hours, sparse at 6 hours, and enriched from 12 hours onwards in central regions of PNP explants (Fig. S6). The mesenchymal markers FN protein and HNK1 epitope appeared from 12 hours onwards (Fig. S6 & S7). In APS explants, both SNAI2 mRNA and SNAI2 protein were present at 0 hours (Fig. S10 & S11), consistent with the endogenous EMT state of APS tissue. Similar to PNP explants, SNAI2 mRNA expression became progressively restricted to patches with subsequent incubation in APS explants (Fig. S11). pSMAD1 protein was absent at 0 hours, robust at 6 hours, and enriched from 12 hours onwards in central regions of APS explants (Fig. S9). Both FN protein and HNK1 epitope appeared from 6 hours onwards (Fig. S9 & S10). A summary of the observed patterns of protein and mRNA expression in PNP and APS explants at different time points can be seen in Supplementary Figure 12 (Fig. S12).

2) New results text in section "SMAD1 upregulation couples EMT initiation to BMP signalling activity":

Pseudo-timecourse analysis of SMAD1 mRNA expression in PNP explants further confirmed this: SMAD1 mRNA was absent at 0 hours in PNP explants and appeared from 6 hours onwards, thus coinciding with the onset of SNAI2 expression (Fig. S8). …This was further confirmed using pseudo-timecourse analysis of SMAD1 mRNA expression in APS explants (Fig. S11).

4) EMT initiation is primarily inferred from SNAI2 expression, loss of polarity markers, and early transcriptional changes. However, the depth, heterogeneity, and maturation of EMT states are not explored. Canonical mesenchymal markers (e.g. vimentin, FN1, integrins) are not clearly shown or discussed in the explant system, and migration is analysed largely at the level of bulk explant spreading. Given the extensive literature on partial and context-specific EMT states, it would be important to clarify whether APS and PNP explants undergo comparable or distinct EMT programs, and whether differences in cell migration dynamics reflect EMT state, fate specification, or tissue geometry; authors are also invited to discuss this in the discussion.

We agree with the reviewer that EMT is a highly plastic process that is likely to be modulated over time within the explants, and to occur differently depending on their tissue of origin. We feel that a full characterisation of these differences would be beyond the scope of this article, though feel that we have establishing a strong framework for doing so in future work. We have pointed to this in revised discussion text:

Section "EMT initiation triggers BMP pathway activation to coordinate collective cell invasion":
While APS and PNP explants share a common substrate-induced EMT programme, our pseudo-timecourse analysis reveals the progressive maturation of EMT states across both tissues, allowing us to begin to disentangle whether differences in collective invasion dynamics reflect EMT state, fate specification, or tissue geometry. These questions will be important to resolve fully in future work, particularly given the extensive literature demonstrating that partial and context-specific EMT states can produce distinct migratory behaviours (reviewed in Campbell and Casanova, 2016).

Specific Comments

1. SNAI2 expression at the 0 h explant time point is inferred from in vivo embryos and bulk RNA-seq but is not directly visualized in freshly dissected explants.

This is now shown in Fig. S10.

2. pSMAD1 is interpreted as nuclear, but nuclear counterstains and proper quantifications (e.g. nuc/cyto ratio) in situ are not shown. Reduced pSMAD1 at the explant periphery could reflect out-of-plane nuclei rather than true signalling differences.

We agree that rigorous quantification of pSMAD1 localisation is essential for confident interpretation, and we would like to clarify an aspect of our analysis that address this concern. In our *ex vivo* explant system, pSMAD1 signal was normalised to SYTOX nuclear counterstain intensity (Figure S9). This normalisation directly accounts for potential confounds arising from out-of-plane nuclei or tissue depth variation, since any such effects would proportionally affect both the pSMAD1 and SYTOX signals.

3. While SNAI2 overexpression is sufficient to induce SMAD1 transcription, the authors do not demonstrate that SNAI2 overexpression induces EMT in this context, nor that EMT is required for SMAD1 phosphorylation or BMP pathway activation. Thus, the observed SMAD1 upregulation may reflect a direct transcriptional effect of SNAI2 rather than an EMT-dependent increase in BMP signalling competence.

We now conclude that SNAI2 is sufficient to induce SMAD1 expression and is required for it ex vivo within our explant system. As SNAI2 is a key part of the gene-regulatory network known to drive EMT, we then discuss how this mechanism can enable the reciprocal coupling of the two processes within a revised first paragraph of the discussion.

4. More detailed molecular and dynamic characterization of the APS and PNP explant models would strengthen interpretation of EMT state, heterogeneity, and collective migration mechanisms underlying the reported BMP-dependent invasion phenotypes.-

This has now been added in Figs. S6-S12 (see response to point 3 above).

5. Am important test would be SNAI2 overexpression {plus minus} LDN, followed by pSMAD1/BMP target readouts in EGFP-positive cells, to establish whether SNAI2-driven SMAD1 expression translates into BMP receptor-dependent signalling.

We opted to assay for changes in competence to BMP4 exposure in the context of SNAI2 loss-of-function to address this point (see response to point 2 above). As we observe a direct impact on p-SMAD independently of BMP4 exposure, we have modified our conclusions to clarify an impact on BMP signalling activity, rather than competence.

6. SMAD1 phosphorylation can occur downstream of non-BMP inputs (e.g. TGFβ). Additional pathway dissection would strengthen attribution to BMP signalling.

We have acknowledged that there are likely other signalling pathways being modulated as part of the induced-cell migration assay, but have elected to focus on BMP due to its known roles in mediating the concomitant cell fate changes we observe in the data. This has been clarified in a revised text:

Results text in section "Ex vivo induction of EMT triggers BMP pathway activation":
We focused specifically on BMP signalling due to the cell fate changes observed in both PNP and APS migrating explants (i.e. toward neural crest and posterior primitive streak fates respectively) being consistent with known roles of BMP signalling rather than TGFbeta signalling during chick development. We note that TGFbeta signalling may also be active in this context, but its role in coordinating collective cell behaviours and fate specification falls outside the scope of the present study.

7. BMP inhibition relies on a single inhibitor (LDN193189). Confirmation with an independent inhibitor or ligand neutralization would strengthen specificity.

We see expected changes associated with BMP pathway inhibition that include alterations to pSMAD1 intensity, and expected cell fate changes in both the primitive streak and ectoderm. The question of whether a regulation of specifically BMP competence has been addressed in response to point 2 above.

8. In the explant system, the EMT→BMP direction could be tested with a minimal BMP competence assay: partially attenuate EMT onset and quantify nuclear pSMAD1 and BMP target induction in response to a defined BMP4 pulse ({plus minus} LDN control).

These experiments have been performed as described above.

9. While BMP signalling clearly modulates tissue-scale dispersal downstream of EMT, the underlying cell-level effects remain insufficiently resolved. It is unclear whether BMP acts directly on migratory behaviour or indirectly through fate allocation. This could be addressed by single-cell tracking combined with temporal BMP perturbations.

This is an excellent question, but one that is potentially very difficult to resolve. Given what we have observed, we think it likely that BMP is acting on both processes simultaneously. Part of the impact of this work is to more clearly advocate away from the interpretation of signalling acting within a linear set of causal relationships: in the context of collective invasion, it appears important to associate cell fate decision-making, EMT and migration in a manner that drives a collective response. We have now made this point clearer in a revised discussion of the manuscript.

Discussion text in section "EMT initiation triggers BMP pathway competence to coordinate collective cell invasion":

While it remains unclear whether BMP signalling acts directly on migratory behaviour or indirectly through fate allocation (or both simultaneously), we suggest that attempting to resolve these as separable sequential events may itself be misleading. In the context of collective invasion, cell fate decision-making, EMT, and migration appear to be deeply coupled processes that together drive a coordinated tissue-level response, rather than components of a linear causal chain.

10. Terms such as "EMT initiation zones," "BMP competence," and "collective invasion dynamics" would benefit from explicit operational definitions or moderated phrasing.

We have removed statements relating to BMP competence throughout the text in light of our new data. Additional clarifications are as follows:

Results text in section “Identification of SNAI2 as a candidate regulator of SMAD1 expression”:
...the regions of EMT initiation, defined here as embryonic territories where expression of key EMT transcriptional drivers have started to become enriched relative to surrounding tissues.

Results text in section “BMP signalling modulates cell invasion dynamics”:
By dynamics, we specifically mean the directionality of migration, the rate at which tissues spread, and the rate at which precursor regions are depleted.

Referee #2:

In this study, the authors take advantage of the chicken embryo and its accessibility to two models of collective cell migration-gastrulation and neural crest-to investigate the interplay between EMT initiation and morphogen signaling during morphogenesis. They show that the anterior primitive streak and the prospective neural plate, when cultured on fibronectin, spread and acquire a molecular signature of EMT. Interestingly, this process is accompanied by an upregulation of BMP members and signaling activity.
The authors then demonstrate a correlation between Smad1 and Snail2 expression and show that Snail2 is able to activate Smad1 when overexpressed. This identifies a molecular mechanism linking Smad signaling to the EMT process. Finally, they show that Smad1 signaling is active at embryonic EMT sites, but that it is not required for EMT initiation per se; rather, it plays a role in cell fate (as previously known) and in cell spreading and collective migration.
Overall, this paper is very interesting, as it identifies a new link between BMP/Smad signaling and the EMT process during embryogenesis. This work could represent a cornerstone for understanding how signaling pathways coordinate morphogenetic movements and fate decisions. The study is generally well performed, the data are convincing, and the technical aspects are well described. Although the manuscript is sometimes difficult to follow due to the transitions between different experimental systems, I believe it is very interesting and could be a good fit for EMBO Journal. However, several aspects need to be addressed before I can fully endorse its publication.

Major comments

• I am troubled by the fact that HCR staining against Smad1 and SNAIL2 appears to be almost

perfectly overlapping (Fig. 2D, Fig. S13A), including what looks like extraembryonic, potentially nonspecific staining. I understand that the authors have controlled for bleed-through, but could there be issues related to the amplification steps? Would it be possible to show some degree of differential expression in a wild-type context, as was done in the gain-of-function experiments (Fig. S11B)? According to the single-cell data, this should be feasible.

We have now performed HCR stains with the same probes in HH4-stage embryos with *SNAI2* and *SMAD1* and imaged them from both the dorsal and ventral side to show *SMAD1* expression (but not *SNAI2* expression) in the hypoblast and endoderm cells.

See supplementary figure S17.

• The role of Smad signaling in spreading and collective invasion is claimed for the primitive streak. Is this also the case for the other system (PNP/NC)? Based on Fig. S14, this is not clear. Could the authors provide quantification of neural crest migration in LDN-treated embryos and/or quantification of PNP explant spreading under LDN conditions?

We focussed on the primitive streak for these experiments as we are able to easily transplant size-matched tissue grafts into stage-matched host embryos for the comparison of how BMP modulation impacts collective invasion. Unfortunately, similar experiments between trunk and cranial neural crest regions would be difficult to perform as the cells migrate at very different stages of development, and into different environments. For these technical reasons, we decided to restrict this part of the study to the primitive streak.

• A key result supporting the idea that EMT primes BMP pathway competence is that the PNP-where there is no Snail2, no Smad1, and no phospho-Smad1-undergoes EMT, Snail2 induction, and p-Smad1 activation when cultured on fibronectin, whereas , in contrast, when PNP is cultured in liquid (where EMT is presumably absent), Smad1 phosphorylation is not observed. In parallel APS explants in liquid show p-Smad1 activation, presumably because EMT factors such as Snail2 are already present and can upregulate Smad1 phosphorylation (likely due to local BMP4 signaling after isolation from BMP inhibitors).
First, I think these data (liquid culture) should be included in the main figures rather than the supplemental material. Second, I wonder whether PNP explants survive and/or differentiate as efficiently in liquid culture as APS explants or PNP explants on fibronectin. In Fig. S6B, the PNP explant appears smaller than the APS explant at 24 hours. This control seems important to support the conclusion that EMT itself primes BMP pathway activation.

We thank the reviewer for this suggestion; we have moved these results to the main figure (Fig. 2 B,B’). The viability of these floating explants was confirmed using active Caspase 3 immunostaining after 24 hours of incubation (Fig. S14).

• From Fig. 3C, it is not entirely clear that Snail2 could induces Smad1 and subsequent p-Smad1 activation specifically at EMT initiation sites, as Snail2 expression does not appear particularly strong in the EMT initiation zone. Based on the proposed model, I would expect higher Snail2 expression in the epiblast. Could the authors provide higher-resolution imaging data to clarify this point?

In the embryo context, where EMT onset is a continual process as cells continually enter and exit this primitive streak, a clear spatial-temporal order of events would be difficult to ascertain, even at higher resolution as it would require tracking gene expression dynamics within live cells. However, we can observe this in our explant culture as cell propagate from the site of ECM contact during induced cell migration (see new supplementary figures S6-S12 describing this).

What we have observed in vivo is that SNAI2 protein signal is clearly present in the primitive streak in Fig. 3C, which is the EMT initiation zone in this context, and this is consistent with the extensive literature establishing SNAI2 as a key driver of mesoderm EMT in the chick primitive streak. SNAI2 is also expressed in migrating mesoderm cells that have already completed ingression, as is clearly visible in Fig. 3C and consistent with its known role in maintaining the mesenchymal state. The relevant prediction of our model is that SNAI2 expression in the primitive streak precedes and drives SMAD1 upregulation at that site, which is supported by our HCR co-expression data (Fig. 2D) and our gain-of-function experiments (Fig. 4).

____

Minor comments

• The statement that Snail2 expression precedes Smad1 expression at the end of the fifth paragraph of the Results section is not clearly supported by in vivo data.

We have modified the following parts of the text to clarify this point:

Results text in section “Identification of SNAI2 as a candidate regulator of SMAD1 expression”:
This bimodal distribution of SMAD1 expression in SNAI2-expressing cells is consistent with a model in which SNAI2 and SMAD1 are co-expressed in cells undergoing EMT initiation, with a subset of SNAI2-expressing cells showing high SMAD1 expression.
…Together, these findings demonstrate that SNAI2 and SMAD1 are co-expressed during development within regions of EMT initiation, consistent with SNAI2 acting as a candidate upstream regulator of SMAD1 expression.

Discussion text in section “EMT initiation triggers BMP pathway competence to coordinate collective cell invasion”:
...with a bimodal distribution in which a subset of SNAI2-expressing cells co-express high levels of SMAD1.

• Most of Figure 4 (expect panel D and E) mainly appear as control experiments that could be moved to the supplementary material if needed.

These have been moved to the supplementary figures (Fig. S20).

• Some supplementary figures contain a large amount of data that is difficult to read. Several could be simplified by showing fewer representative examples or more synthetic presentations (e.g., Figs. S4, S7, S11).

The following figures have been revised accordingly:

Supplementary figure S4. Only top 10 terms shown.
Supplementary figure S11. Reduced number of representative images.

• In the graphical abstract, I suggest using an arrow to indicate temporal progression rather than the distinction between "mechanism" and "response," which does not add much to the overall message.

This is an excellent suggestion that we have now implemented.

• One potentially confusing aspect is that the APS, although it does not show p-Smad1 signaling, undergoes massive EMT to form paraxial mesoderm. Even though its spreading behavior appears weaker than that of PPS and can be enhanced by BMP4 (Fig. 6B), this raises the question of what drives collective behavior in the anterior primitive streak if not BMP signaling-perhaps FGF or Wnt pathways? I think this point should be addressed or discussed.

Yes, this is an interesting point that we have now discussed in the revised manuscript. Interestingly, these cells are beginning to delay their ingression rates as has been previously described by us and others:

Discussion text in section "Coordinating precursor region dynamics during chick axis elongation": Notably, the APS undergoes extensive EMT to contribute to paraxial mesoderm despite lacking endogenous BMP signalling, raising the question of what drives its collective behaviour in this context. Interestingly, APS cells at this stage are beginning to slow their ingression rates, consistent with previously described dynamics of precursor region maintenance in the anterior streak (Iimura and Pourquié, 2006; Guillot et al., 2021; Busby et al., 2024). Alternative signalling pathways, including FGF and Wnt, which are known to be active in the primitive streak at this stage (reviewed in Chuai and Weijer, 2009), are strong candidates for coordinating collective cell behaviour in the absence of BMP signalling activity, and represent an interesting avenue for future investigation.

Dear Dr Steventon,

Thank you again for sending us your amended manuscript (EMBOJ-2025-123368R) for reconsideration by The EMBO Journal, together with a point-by-point response to the issues raised during the earlier peer-review. Please accept my apologies for the unusual protraction in getting back to you due to delayed expert input and detailed discussion in the editorial team.

Your revised study was sent back to the referees for their scientific reassessment, and we have received detailed re-reports from both, which I enclose below. As you will see, the reviewers state that the work has been satisfactorily enhanced by the revisions and they can now support publication at the EMBO Journal, pending minor revision.

Thus, we are pleased to inform you that your manuscript has been accepted in principle for publication in The EMBO Journal.

Please carefully consider the remaining minor points still raised by the referees by adjusting the discussion of the results where appropriate.

Also, we now need you to take care of a number of minor issues related to formatting and data annotation, as detailed below.

Please submit a revised version of the manuscript using the link enclosed below.

Thank you again for giving us the chance to consider your manuscript for The EMBO Journal, I look forward to hearing from you and receiving your final revised version of the manuscript.

Best regards,

Daniel Klimmeck

Daniel Klimmeck PhD
Senior Editor
The EMBO Journal

******

Formatting changes required for the revised version of the manuscript:

>> Author Contributions: Remove the author contributions information from the manuscript text. Note that CRediT has replaced the traditional author contributions section as of now because it offers a systematic machine-readable author contributions format that allows for more effective research assessment. and use the free text boxes beneath each contributing author's name to add specific details on the author's contribution.

More information is available in our guide to authors.
https://www.embopress.org/page/journal/14602075/authorguide

>> Please provide the main manuscript text as .docx file with a size below 20MB.

>> Update the headings and the order of the manuscript sections to: Abstract / Introduction / Results / Discussion / Methods / Data Availability / Acknowledgements / Disclosure and Competing Interests Statement / References / Figure Legends / Expanded View Figure Legends

>> Remove graphical abstract and highlights from the manuscript text.

>> Funding information: please add 'Herchel Smith PhD Studentship' and 'Research Support grant to Y.T.' to the list of funders in our online system.

>> References: please adjust reference format to EMBO Journal format, 10 authors et al.; In the text of the manuscript, a reference should be cited by author and year of publication; no more than two authors may be cited per reference; 'et al' should be used if there are more than two authors (i.e. Smith & Jones, 2003; Smith et al, 2000). In the reference list, citations should be listed in alphabetical order and then chronologically, with the authors' surnames and initials inverted; where there are more than 10 authors on a paper, 10 will be listed, followed by 'et al.'.

>> Figures in separate files: Remove the main figures and EV figures from the manuscript text and move the figure legends to the end of the manuscript and correct the nomenclature to "Figure EV1" etc. Remove the appendix from the manuscript text.

>>Appendix file with ToC: please move the figure legends underneath the corresponding figures and add a table of contents, including page numbers. Please correct the nomenclature to "Appendix Figure S1" etc. .

>> Data availability section: Please remove the "Resource availability", "Lead Contact and "Materials availability" from the manuscript text. Please also remove "Microscopy data reported in this paper will be shared by the lead contact upon reasonable request." and "Any additional information required to reanalyse the data reported in this paper is available from the lead contact upon reasonable request.". Change wording from 'discussed' to 'generated' datasets. Add URLs for the data deposited in the GEO database.

>> Add a separate 'Statistical Analysis' section to the Methods part, detailing the algorithms and statistical tests applied.

>> Please indicate redisplay of data from Fig 1F, 2E in the figure legend of Appendix Fig S9, between Fig 5F and Fig 3A, Fig 3B and Fig 5A, Fig 3A and Fig 1F, respectively.

>> Consider additional changes and comments from our production team as indicated below:

Please note that the specific URLs for datasets (GSE312350, GSE314634) are not provided in the data availability statement.
- Figure legends:
1. Please note that the specific URLs for datasets (GSE312350, GSE314634) are not provided in the data availability statement.
2. Please note that the box plots need to be defined in terms of minima, maxima, centre, bounds of box and whiskers, and percentile in the legends of figures 5B,B'; 6F-I.
3. Please note that information related to n is missing in the legends of figures 1E,E'; 2A,B'; 5B,B'; 6F-I.
4. Please note that scale bar and its definition are missing for figure EV-4C.

Further information is available in our Guide For Authors: https://link.springer.com/journal/44318/submission-

guidelines

Referee #1:

I thank the authors for their careful revision; they have addressed the major conceptual and experimental concerns raised in the original review through new experiments, improved characterization of the explant model, and a more balanced interpretation of the data. I believe the manuscript is now suitable for publication. Below are only a few minor suggestions that may further strengthen the conceptual clarity and broader impact of the study.
Minor comments
1) To further highlight the conceptual advance, it would be helpful to more explicitly explain why the prevailing linear BMP→EMT model is incomplete in explaining the coordination between EMT and fate specification, and how the proposed SNAI2-SMAD1-BMP regulatory axis extends current understanding rather than simply adding another regulatory interaction.
2) Overall, the revised data support a model in which SNAI2 links an early EMT-associated cell state to SMAD1 expression and BMP pathway activity. I encourage the authors to formulate their conclusions primarily around this SNAI2-SMAD1 regulatory axis and present EMT as the broader biological context in which this mechanism operates. This framing more closely reflects the experimental evidence while preserving the conceptual significance of the proposed model.
3) One interesting implication of the proposed model concerns its temporal organization. It is not entirely clear how the reader should interpret the sequence of events proposed by the authors. The manuscript proposes that EMT initiation primes BMP pathway activity through the SNAI2-SMAD1 axis, yet BMP pathway activity subsequently becomes restricted to EMT initiation zones despite persistent SNAI2 expression in migrating cells. The revised data therefore define an early SNAI2-SMAD1-BMP regulatory module, whereas later mesenchymal features, such as FN expression, emerge during EMT progression. This raises the question of how the authors envisage the transition from this early signalling programme to the later migratory EMT programme. More specifically, what molecular programmes are proposed to sustain the migratory state once the transient BMP signalling phase has subsided? It would therefore be valuable if the authors could briefly discuss the conceptual relationship between the early SNAI2-BMP signalling module and the later EMT programme. For example, do they envisage continued SNAI2 activity, downstream EMT effector programmes, ECM remodelling (illustrated here by FN expression?), cell-ECM interactions, or cytoskeletal remodelling as contributing to the maintenance of migration? Such discussion would clarify the temporal logic of the proposed model and further strengthen its broader implications.

Referee #2:

The authors have successfully addressed most of my previous comments. I am still very enthusiastic about the manuscript and even more so since the additional experiments, in particular the temporal analysis of the in vitro in terms of Snail2/Smad activation events and the new Snail loss-of-function data, substantially strengthen the manuscript and increase its overall value. Nevertheless, I still have a few remaining minor concerns that I believe need to be addressed to further improve the clarity and readability of the manuscript before publication.
First, I am now convinced that the SNAIL2/SMAD1 HCR co-staining is genuine rather than an artefact, as the new images clearly show specific staining.

I understand why the authors restricted their in vivo analyses to the primitive streak, as extending these experiments to the neural crest would likely be technically challenging. However, I do not understand why similar pharmacological inhibition of SMAD signalling could not be performed on PNP explants, given that the culture of these explants is already shown by the authors in Figure 1B. Measuring the effect of SMAD inhibition on explant spreading would provide an additional functional test of the proposed mechanism.

I appreciate that the authors moved the liquid-culture explant experiment into the main figures, as I had suggested, and that they included an apoptosis analysis (Supplementary Figures). In my opinion, this remains one of the strongest pieces of evidence supporting the conclusion that cell migration is required for pSMAD1 upregulation. Otherwise, one could argue that BMP signalling activation simply reflects the progression of the developmental programme following explant culture, independently of migration. For this reason, I think the conclusions drawn in the text before this experiment should be phrased somewhat more cautiously. Statements such as "Together, these findings suggest or indicate that substrate-induced cell migration triggers cell fate responses across germ layers" would be more appropriate than "demonstrate."

Concerning SNAIL2 expression in the mesoderm, I appreciate the authors' explanation that Snail2 is likely required for the maintenance of the mesenchymal state, and I agree that this is a plausible interpretation. I can now appreciate the weak Snail2 staining in the epiblast. However, pSMAD1 is also present in the epiblast and appears to extend beyond the Snail2-positive domain, which likely contributed to my initial confusion. A brief discussion of this point could help readers. I also acknowledge

that the new summary figures (Supplementary Figures) greatly help in understanding the dynamics of the process. One point where I still disagree with the interpretation concerns the statement that "The mesenchymal markers FN protein and HNK1 epitope appeared from 12 hours onwards (Fig. A4 & A5)." From the figures, both markers appear to be already detectable at 6 hours, although perhaps at lower levels. This statement should therefore be reconsidered.

Finally, as mentioned, the new Snail knockdown experiments represent a significant improvement to the manuscript. Because these experiments now reveal a phenotype, I examined them more carefully than in the previous version and have a few questions. First, unless I have overlooked it, I wonder whether the appropriate CRISPR control would be a non-targeting (scrambled) sgRNA cloned into the same pCU6.3 vector together with PCAG-Cas9, rather than the PCAG-Cas9-only control, as was done in the Williams et al. (2018) study cited by the authors. Second, I found Figures 6E-J difficult to interpret. It would be helpful if the authors could explain the quantification procedure more clearly. From the figure legend, I infer that Citrine-positive cells were segmented and used as a mask within which the HCR/immunostaining signal was quantified, but this is not entirely clear. What exactly does the y-axis represent? n= number of cells I assume; what are the sample sizes (number of embryos and independent experiments)? Were these measurements performed on embryos processed by double HCR, or were they obtained independently? I could not find (maybe I missed it) these methodological details in either the main text or the supplementary methods.
I found the distinction between the different axes of these quantitative analyses (Figures 6E-J) somewhat confusing. If I understand correctly, some analyses reflect changes in overall expression levels (horizontal axis), whereas others quantify the proportion of cells (vertical axis). For example, Figure 6H seems to indicate lower HCR signal per cell in fewer cells, whereas Figure 6I appears to show reduced immunostaining signal in a larger proportion of cells. It is not entirely clear how these measurements relate to one another. A more explicit explanation of what each analysis measures, and why multiple metrics were used, would greatly improve the readability of this section. More generally, I feel that the manuscript currently devotes considerable space to technical details that are not essential to the main narrative (for example, some aspects of the validation of the CRISPR approach, which could easily be moved to the supplementary methods without detracting from the message), while other key methodological aspects underlying the quantitative analyses (such as those in Figures 6H-J) would benefit from a more detailed explanation.

Referee #1:

I thank the authors for their careful revision; they have addressed the major conceptual and experimental concerns raised in the original review through new experiments, improved characterization of the explant model, and a more balanced interpretation of the data. I believe the manuscript is now suitable for publication. Below are only a few minor suggestions that may further strengthen the conceptual clarity and broader impact of the study.
Minor comments

1) To further highlight the conceptual advance, it would be helpful to more explicitly explain why the prevailing linear BMP→EMT model is incomplete in explaining the coordination between EMT and fate specification, and how the proposed SNAI2-SMAD1-BMP regulatory axis extends current understanding rather than simply adding another regulatory interaction.

We have now included a revised conclusion statement to make this point clearer, in reference to a summary diagram of our model (Figure 7).

2) Overall, the revised data support a model in which SNAI2 links an early EMT-associated cell state to SMAD1 expression and BMP pathway activity. I encourage the authors to formulate their conclusions primarily around this SNAI2-SMAD1 regulatory axis and present EMT as the broader biological context in which this mechanism operates. This framing more closely reflects the experimental evidence while preserving the conceptual significance of the proposed model.

We have now adjusted our conclusion statements throughout the manuscript as suggested by the reviewer.

3) One interesting implication of the proposed model concerns its temporal organization. It is not entirely clear how the reader should interpret the sequence of events proposed by the authors. The manuscript proposes that EMT initiation primes BMP pathway activity through the SNAI2-SMAD1 axis, yet BMP pathway activity subsequently becomes restricted to EMT initiation zones despite persistent SNAI2 expression in migrating cells. The revised data therefore define an early SNAI2-SMAD1-BMP regulatory module, whereas later mesenchymal features, such as FN expression, emerge during EMT progression. This raises the question of how the authors envisage the transition from this early signalling programme to the later migratory EMT programme. More specifically, what molecular programmes are proposed to sustain the migratory state once the transient BMP signalling phase has subsided? It would therefore be valuable if the authors could briefly discuss the conceptual relationship between the early SNAI2-BMP signalling module and the later EMT programme. For example, do they envisage continued SNAI2 activity, downstream EMT effector programmes, ECM remodelling (illustrated here by FN expression?), cell-ECM interactions, or cytoskeletal remodelling as contributing to the maintenance of migration? Such discussion would clarify the temporal logic of the proposed model and further strengthen its broader implications.

We have observed that BMP is required in vivo to tune the dynamics of collective cell invasion, leading to faster dispersal of cells from the primitive streak. However, inhibition of BMP in our in vitro assay does not inhibit the onset of migration for either PNP or APS explants. The mechanism by which BMP modulates collective behaviour is not known, and will be important to determine in future work. What we can say is that this elicited by the pathway by setting their state at the onset of migration, as pSMAD1 is decreased upon cell migration in vivo and in vitro. These points have been made clearer as part of the revised conclusions paragraph of the paper.

Referee #2:

The authors have successfully addressed most of my previous comments. I am still very enthusiastic about the manuscript and even more so since the additional experiments, in particular the temporal analysis of the in vitro in terms of Snail2/Smad activation events and the new Snail loss-of-function data, substantially strengthen the manuscript and increase its overall value. Nevertheless, I still have a few remaining minor concerns that I believe need to be addressed to further improve the clarity and readability of the manuscript before publication.
First, I am now convinced that the SNAIL2/SMAD1 HCR co-staining is genuine rather than an artefact, as the new images clearly show specific staining.

I understand why the authors restricted their in vivo analyses to the primitive streak, as extending these experiments to the neural crest would likely be technically challenging. However, I do not understand why similar pharmacological inhibition of SMAD signalling could not be performed on PNP explants, given that the culture of these explants is already shown by the authors in Figure 1B. Measuring the effect of SMAD inhibition on explant spreading would provide an additional functional test of the proposed mechanism.

This is an important point by the reviewer, PNP explants treated with LDN are shown in Figure EV5 where we see similar results to the inhibition of BMP signalling on APS explants.

I appreciate that the authors moved the liquid-culture explant experiment into the main figures, as I had suggested, and that they included an apoptosis analysis (Supplementary Figures). In my opinion, this remains one of the strongest pieces of evidence supporting the conclusion that cell migration is required for pSMAD1 upregulation. Otherwise, one could argue that BMP signalling activation simply reflects the progression of the developmental programme following explant culture, independently of migration. For this reason, I think the conclusions drawn in the text before this experiment should be phrased somewhat more cautiously. Statements such as "Together, these findings suggest or indicate that substrate-

induced cell migration triggers cell fate responses across germ layers" would be more appropriate than "demonstrate."

We have now edited the wording of this conclusion as suggested.

Concerning SNAIL2 expression in the mesoderm, I appreciate the authors' explanation that Snail2 is likely required for the maintenance of the mesenchymal state, and I agree that this is a plausible interpretation. I can now appreciate the weak Snail2 staining in the epiblast. However, pSMAD1 is also present in the epiblast and appears to extend beyond the Snail2-positive domain, which likely contributed to my initial confusion. A brief discussion of this point could help readers. I also acknowledge that the new summary figures (Supplementary Figures) greatly help in understanding the dynamics of the process.

The reviewer is correct that this suggests that additional mechanisms of Smad1 expression operate in vivo to drive its expression in a broader region of epiblast than SNAI2. We have now made a point of this in the context of our CRISPR experiments.

One point where I still disagree with the interpretation concerns the statement that "The mesenchymal markers FN protein and HNK1 epitope appeared from 12 hours onwards (Fig. A4 & A5)." From the figures, both markers appear to be already detectable at 6 hours, although perhaps at lower levels. This statement should therefore be reconsidered.

We have now modified this statement.

Finally, as mentioned, the new Snail knockdown experiments represent a significant improvement to the manuscript. Because these experiments now reveal a phenotype, I examined them more carefully than in the previous version and have a few questions. First, unless I have overlooked it, I wonder whether the appropriate CRISPR control would be a non-targeting (scrambled) sgRNA cloned into the same pCU6.3 vector together with PCAG-Cas9, rather than the PCAG-Cas9-only control, as was done in the Williams et al. (2018) study cited by the authors.

We agree that using a scrambled sgRNA control would have been an acceptable alternative control for this experiment. However, we elected to use a Cas9 only negative control, as we were concerned about any reported off-target effects of a using scrambled sgRNA in other contexts (https://www.nature.com/articles/srep34524#Sec2).

Second, I found Figures 6E-J difficult to interpret. It would be helpful if the authors could explain the quantification procedure more clearly. From the figure legend, I infer that Citrine-positive cells were segmented and used as a mask within which the HCR/immunostaining signal was quantified, but this is not entirely clear. What exactly does the y-axis represent?

n= number of cells I assume; what are the sample sizes (number of embryos and independent experiments)? Were these measurements performed on embryos processed by double HCR, or were they obtained independently? I could not find (maybe I missed it) these methodological details in either the main text or the supplementary methods.

I found the distinction between the different axes of these quantitative analyses (Figures 6E-J) somewhat confusing. If I understand correctly, some analyses reflect changes in overall expression levels (horizontal axis), whereas others quantify the proportion of cells (vertical axis). For example, Figure 6H seems to indicate lower HCR signal per cell in fewer cells, whereas Figure 6I appears to show reduced immunostaining signal in a larger proportion of cells. It is not entirely clear how these measurements relate to one another. A more explicit explanation of what each analysis measures, and why multiple metrics were used, would greatly improve the readability of this section. More generally, I feel that the manuscript currently devotes considerable space to technical details that are not essential to the main narrative (for example, some aspects of the validation of the CRISPR approach, which could easily be moved to the supplementary methods without detracting from the message), while other key methodological aspects underlying the quantitative analyses (such as those in Figures 6H-J) would benefit from a more detailed explanation.

We have now provided additional explanations of this experiment in the main text and figure legends.

Dear Dr Steventon,

Thank you for submitting the revised version of your manuscript. I have now evaluated your amended manuscript and concluded that the remaining minor concerns have been sufficiently addressed.

I am thus pleased to inform you that your manuscript has been accepted for publication in the EMBO Journal.

Your manuscript will be processed for publication by EMBO Press. It will be copy edited and you will receive page proofs prior to publication. Please note that you will be contacted by Springer Nature Author Services to complete licensing and payment information.

Please note that it is The EMBO Journal policy for the transcript of the editorial process (containing referee reports and your response letter) to be published as an online supplement to each paper. If you do NOT want this, you will need to inform the Editorial Office via email immediately. More information is available here: https://www.embopress.org/transparent-process#Review_Process

You may qualify for financial assistance for your publication charges - either via a Springer Nature fully open access agreement or an EMBO initiative. Check your eligibility: https://www.embopress.org/page/journal/14602075/authorguide#chargesguide

Should you be planning a Press Release on your article, please notify the production team via this form in order to coordinate publication and release dates: https://forms.cloud.microsoft/e/eH4A8JVP6g

If you have any questions, please do not hesitate to contact the Editorial Office. Thank you for your contribution to The EMBO Journal.

Thank you again for this contribution to The EMBO Journal and congratulations on a successful publication! Please consider us again in the future for your most exciting work.

Best regards,

Daniel Klimmeck

Daniel Klimmeck, PhD
Senior Editor
The EMBO Journal
EMBO
Postfach 1022-40
Meyerhofstrasse 1
D-69117 Heidelberg
contact@embojournal.org

*****
Please note that it is The EMBO Journal policy for the transcript of the editorial process (containing referee reports and your response letters) to be published as an online supplement to each paper. If you should prefer removal of any referee-only figures included in the point-by-point response(s), e.g. because they may still be used for future publication or because they have been reproduced from published work by others, please do let us know immediately via response email.
More information is available here: https://link.springer.com/partners/embo-press/editorial-policies#Peer%20review

EMBO Press Author Checklist

Corresponding Author Name:	
Journal Submitted to:	
Manuscript Number:	

USEFUL LINKS FOR COMPLETING THIS FORM
The EMBO Journal - Author Guidelines
EMBO Reports - Author Guidelines
Molecular Systems Biology - Author Guidelines
EMBO Molecular Medicine - Author Guidelines

Reporting Checklist for Life Science Articles (updated January
This checklist is adapted from Materials Design Analysis Reporting (MDAR) Checklist for Authors. MDAR establishes a minimum set of requirements in transparent reporting in the life sciences (see Statement of Task: 10.31222/osf.io/9sm4x). Please follow the journal's guidelines in preparing your
Please note that a copy of this checklist will be published alongside your article.

Abridged guidelines for figures
1. Data
The data shown in figures should satisfy the following conditions:
- the data were obtained and processed according to the field's best practice and are presented to reflect the results of the experiments in an accurate and unbiased manner.
- ideally, figure panels should include only measurements that are directly comparable to each other and obtained with the same assay.
- plots include clearly labeled error bars for independent experiments and sample sizes. Unless justified, error bars should not be shown for technical
- if n<5, the individual data points from each experiment should be plotted. Any statistical test employed should be justified.
- Source Data should be included to report the data underlying figures according to the guidelines set out in the authorship guidelines on Data

2. Captions
Each figure caption should contain the following information, for each panel where they are relevant:
- a specification of the experimental system investigated (eg cell line, species name).
- the assay(s) and method(s) used to carry out the reported observations and measurements.
- an explicit mention of the biological and chemical entity(ies) that are being measured.
- an explicit mention of the biological and chemical entity(ies) that are altered/varied/perturbed in a controlled manner.
- the exact sample size (n) for each experimental group/condition, given as a number, not a range;
- a description of the sample collection allowing the reader to understand whether the samples represent technical or biological replicates (including how many animals, litters, cultures, etc.).
- a statement of how many times the experiment shown was independently replicated in the laboratory.
- definitions of statistical methods and measures:
  - common tests, such as t-test (please specify whether paired vs. unpaired), simple χ2 tests, Wilcoxon and Mann-Whitney tests, can be unambiguously identified by name only, but more complex techniques should be described in the methods section;
  - are tests one-sided or two-sided?
  - are there adjustments for multiple comparisons?
  - exact statistical test results, e.g., P values = x but not P values < x;
  - definition of 'center values' as median or average;
  - definition of error bars as s.d. or s.e.m.

Please complete ALL of the questions below.
Select "Not Applicable" only when the requested information is not relevant for your study.

Materials

Newly Created Materials	Information included in the manuscript?	In which section is the information available? (Reagents and Tools Table, Materials and Methods, Figures, Data Availability Section)
New materials and reagents need to be available; do any restrictions apply?	Not Applicable	

Antibodies	Information included in the manuscript?	In which section is the information available? (Reagents and Tools Table, Materials and Methods, Figures, Data Availability Section)
For **antibodies** provide the following information:<br>- Commercial antibodies: RRID (if possible) or supplier name, catalogue number and or/clone number<br>- Non-commercial: RRID or citation	Yes	Reagents and tools table and material and methods

DNA and RNA sequences	Information included in the manuscript?	In which section is the information available? (Reagents and Tools Table, Materials and Methods, Figures, Data Availability Section)
**Short novel DNA or RNA including primers, probes**: provide the sequences.	Not Applicable	

Cell materials	Information included in the manuscript?	In which section is the information available? (Reagents and Tools Table, Materials and Methods, Figures, Data Availability Section)
**Cell lines:** Provide species information, strain. Provide accession number in repository **OR** supplier name, catalog number, clone number, and/**OR** RRID.	Not Applicable	
**Primary cultures:** Provide species, strain, sex of origin, genetic modification status.	Not Applicable	
Report if the cell lines were recently **authenticated** (e.g., by STR profiling) and tested for mycoplasma contamination.	Not Applicable	

Experimental animals	Information included in the manuscript?	In which section is the information available? (Reagents and Tools Table, Materials and Methods, Figures, Data Availability Section)
**Laboratory animals or Model organisms:** Provide species, strain, sex, age, genetic modification status. Provide accession number in repository **OR** supplier name, catalog number, clone number, **OR** RRID.	Yes	Materials and methods
**Animal observed in or captured from the field:** Provide species, sex, and age where possible.	Not Applicable	
Please detail **housing and husbandry conditions**.	Not Applicable	

Plants and microbes	Information included in the manuscript?	In which section is the information available? (Reagents and Tools Table, Materials and Methods, Figures, Data Availability Section)
**Plants:** provide species and strain, ecotype and cultivar where relevant, unique accession number if available, and source (including location for collected wild specimens).	Not Applicable	
**Microbes:** provide species and strain, unique accession number if available, and source.	Not Applicable	

Human research participants	Information included in the manuscript?	In which section is the information available? (Reagents and Tools Table, Materials and Methods, Figures, Data Availability Section)
If collected and within the bounds of privacy constraints report on age, sex and gender or ethnicity for all study participants.	Not Applicable	

Core facilities	Information included in the manuscript?	In which section is the information available? (Reagents and Tools Table, Materials and Methods, Figures, Data Availability Section)
If your work benefited from core facilities, was their service mentioned in the acknowledgments section?	Yes	Acknowledgements section

Design

Study protocol	Information included in the manuscript?	In which section is the information available? (Reagents and Tools Table, Materials and Methods, Figures, Data Availability Section)
If study protocol has been **pre-registered, provide DOI in the manuscript**. For clinical trials, provide the trial registration number **OR** cite DOI.	Not Applicable	
Report the **clinical trial registration number** (at ClinicalTrials.gov or equivalent), where applicable.	Not Applicable	

Laboratory protocol	Information included in the manuscript?	In which section is the information available? (Reagents and Tools Table, Materials and Methods, Figures, Data Availability Section)
Provide DOI OR other citation details if **external detailed step-by-step protocols** are available.	Not Applicable	

Experimental study design and statistics	Information included in the manuscript?	In which section is the information available? (Reagents and Tools Table, Materials and Methods, Figures, Data Availability Section)
Include a statement about **sample size** estimate even if no statistical methods were used.	Yes	In figure legends and main text
Were any steps taken to minimize the effects of subjective bias when allocating animals/samples to treatment (e.g. **randomization procedure**)? If yes, have they been described?	Not Applicable	
Include a statement about **blinding** even if no blinding was done.	Yes	In methods
Describe **inclusion/exclusion criteria** if samples or animals were excluded from the analysis. Were the criteria pre-established?<br><br>If sample or data points were omitted from analysis, report if this was due to attrition or intentional exclusion and provide justification.	Not Applicable	
For every figure, are **statistical tests** justified as appropriate? Do the data meet the assumptions of the tests (e.g., normal distribution)? Describe any methods used to assess it. Is there an estimate of variation within each group of data? Is the variance similar between the groups that are being statistically compared?	Yes	In main text and figure legends

Sample definition and in-laboratory replication	Information included in the manuscript?	In which section is the information available? (Reagents and Tools Table, Materials and Methods, Figures, Data Availability Section)
In the figure legends: state number of times the experiment was **replicated** in laboratory.	Yes	
In the figure legends: define whether data describe **technical or biological replicates**.	Yes	

Ethics

Ethics	Information included in the manuscript?	In which section is the information available? (Reagents and Tools Table, Materials and Methods, Figures, Data Availability Section)
Studies involving **human participants**: State details of **authority granting ethics approval** (IRB or equivalent committee(s), provide reference number for approval.	Not Applicable	
Studies involving **human participants**: Include a statement confirming that **informed consent** was obtained from all subjects and that the experiments conformed to the principles set out in the WMA Declaration of Helsinki and the Department of Health and Human Services Belmont Report.	Not Applicable	
Studies involving **human participants:** For publication of **patient photos**, include a statement confirming that consent to publish was obtained.	Not Applicable	
Studies involving experimental **animals**: State details of **authority granting ethics approval** (IRB or equivalent committee(s), provide reference number for approval. Include a statement of compliance with ethical regulations.	Not Applicable	
Studies involving **specimen and field samples:** State if relevant **permits** obtained, provide details of authority approving study; if none were required, explain why.	Not Applicable	

Dual Use Research of Concern (DURC)	Information included in the manuscript?	In which section is the information available? (Reagents and Tools Table, Materials and Methods, Figures, Data Availability Section)
Could your study fall under dual use research restrictions? Please check biosecurity documents and list of **select agents and toxins** (CDC): https://www.selectagents.gov/sat/list.htm	Not Applicable	
If you used a select agent, is the security level of the lab appropriate and reported in the manuscript?	Not Applicable	
If a study is subject to dual use research of concern regulations, is the name of the **authority granting approval and reference number** for the regulatory approval provided in the manuscript?	Not Applicable	

Reporting

The MDAR framework recommends adoption of discipline-specific guidelines, established and endorsed through community initiatives. Journals have their own policy about requiring specific guidelines and recommendations to complement MDAR.

Adherence to community standards	Information included in the manuscript?	In which section is the information available? (Reagents and Tools Table, Materials and Methods, Figures, Data Availability Section)
State if relevant guidelines or checklists (e.g., **ICMJE, MIBBI, ARRIVE, PRISMA**) have been followed or provided.	Not Applicable	
For **tumor marker prognostic studies,** we recommend that you follow the **REMARK** reporting guidelines (see link list at top right). See author guidelines, under 'Reporting Guidelines'. Please confirm you have followed these guidelines.	Not Applicable	
For **phase II and III randomized controlled trials**, please refer to the **CONSORT** flow diagram (see link list at top right) and submit the CONSORT checklist (see link list at top right) with your submission. See author guidelines, under 'Reporting Guidelines'. Please confirm you have submitted this list.	Not Applicable	

Data Availability

Data availability	Information included in the manuscript?	In which section is the information available? (Reagents and Tools Table, Materials and Methods, Figures, Data Availability Section)
Have **primary datasets** been deposited according to the journal's guidelines (see 'Data Deposition' section) and the respective accession numbers provided in the Data Availability Section?	Yes	Data Availability section
Were **human clinical and genomic datasets** deposited in a public access-controlled repository in accordance to ethical obligations to the patients and to the applicable consent agreement?	Not Applicable	
Are **computational models** that are central and integral to a study available without restrictions in a machine-readable form? Were the relevant accession numbers or links provided?	Not Applicable	
If publicly available data were reused, provide the respective **data citations in the reference list**.	Not Applicable	
