# Supplementary material for: Reciprocal coupling between EMT and BMP morphogen signalling drives collective cell invasion
Source: EMBO J. 2026 Oct 7;45(21):7347–73. doi: 10.1038/s44318-026-00916-8 (PMC13728173; doi:10.1038/s44318-026-00916-8)
Supplement: Supplementary file 7 [file 44318_2026_916_MOESM4_ESM.zip › EMBOJ-2025-123368R1-Movie_EV2/MovieEV2 legend.rtf]

Movie EV2. Widefield imaging of control and treated APS and PPS explants migrating on human fibronectin-coated glass. Left: APS explants. Second from left: APS explants treated with exogenous BMP4. Second from right: PPS explants treated with LDN. Right: PPS explants.
