# Supplementary material for: Reciprocal coupling between EMT and BMP morphogen signalling drives collective cell invasion
Source: EMBO J. 2026 Oct 7;45(21):7347–73. doi: 10.1038/s44318-026-00916-8 (PMC13728173; doi:10.1038/s44318-026-00916-8)
Supplement: Supplementary file 8 [file 44318_2026_916_MOESM5_ESM.zip › Datasets/Dataset EV1.zip!/Dataset EV1/Legend.rtf]

Dataset EV1. StringTie-derived gene counts from bulk RNA sequencing. Gene-level read counts generated using StringTie from bulk RNA sequencing data. Sample names were changed for clarity: “MSP” samples are the APS-0h samples, “MSPex” samples are the APS-24h samples, “NP” samples are the PNP-0h samples, and “NPex” samples are the PNP-24h samples.
